# Clinical development of targeted and immune based anti-cancer therapies

**DOI:** 10.1186/s13046-019-1094-2

**Published:** 2019-04-11

**Authors:** N. A. Seebacher, A. E. Stacy, G. M. Porter, A. M. Merlot

**Affiliations:** 1 Faculty of Medicine, The University of Sydney, Camperdown, New South Wales 2006 Australia; 2 Faculty of Medicine, The University of Notre Dame, Darlinghurst, New South Wales 2010 Australia; 3 Children’s Cancer Institute, Lowy Cancer Research Centre, University of New South Wales, Kensington, New South Wales 2031 Australia; 4 School of Women’s and Children’s Health, Faculty of Medicine, University of New South Wales, Kensington, New South Wales 2031 Australia; 5 UNSW Centre for Childhood Cancer Research, Faculty of Medicine, University of New South Wales, Kensington, New South Wales 2031 Australia

**Keywords:** Targeted therapies, Small molecule inhibitors, Monoclonal antibodies, Immunotherapies, Clinical trials

## Abstract

Cancer is currently the second leading cause of death globally and is expected to be responsible for approximately 9.6 million deaths in 2018. With an unprecedented understanding of the molecular pathways that drive the development and progression of human cancers, novel targeted therapies have become an exciting new development for anti-cancer medicine. These targeted therapies, also known as biologic therapies, have become a major modality of medical treatment, by acting to block the growth of cancer cells by specifically targeting molecules required for cell growth and tumorigenesis. Due to their specificity, these new therapies are expected to have better efficacy and limited adverse side effects when compared with other treatment options, including hormonal and cytotoxic therapies. In this review, we explore the clinical development, successes and challenges facing targeted anti-cancer therapies, including both small molecule inhibitors and antibody targeted therapies. Herein, we introduce targeted therapies to epidermal growth factor receptor (EGFR), vascular endothelial growth factor (VEGF), human epidermal growth factor receptor 2 (HER2), anaplastic lymphoma kinase (ALK), BRAF, and the inhibitors of the T-cell mediated immune response, cytotoxic T-lymphocyte-associated protein 4 (CTLA-4) and programmed cell death protein-1 (PD-1)/ PD-1 ligand (PD-1 L).

## Background

Globally, around 1 in 6 deaths are attributed to cancer, making it the second leading cause of death [1]. In 2018, it is estimated that cancer will account for 9.6 million deaths [1]. The current mainstays of cancer therapy, which includes radiation therapy, surgery, and systemic chemotherapy, have several drawbacks that limits their efficacy in the clinic. For example, radiation therapy frequently causes indirect damage to surrounding tissues leading to wound complications and poor healing; surgery may miss microscopic and metastatic disease; and chemotherapy often results in systemic toxicities and the development of drug resistance [2–6]. Therefore, there have been efforts to develop better clinical agents with more targeted actions and fewer drawbacks, including reduced side effects. This has led to the development of agents that more specifically target tumorigenic pathways and, more recently, those that control immune checkpoints.

Most anti-cancer therapies to date have been designed to interfere with the molecular drivers of tumorigenesis, i.e., the molecules necessary for tumor growth and progression. Traditional cytotoxic chemotherapies usually target rapidly proliferating cancer cells by interfering with cell division [7]. However, this also non-specifically targets rapidly-dividing healthy cells, such as bone marrow and hair cells, producing the well-recognized side effects of chemotherapy [7]. Therefore, a primary goal of targeted therapies is to act with greater precision to reduce these side effects. Targeted anti-cancer agents are broadly classified into small-molecule inhibitors and monoclonal antibodies (mAbs).

Small-molecule inhibitors, which end with the stem “-ib”, are usually ≤500 Da in size, allowing translocation through the plasma membrane to interact with the cytoplasmic domain of cell-surface receptors or intracellular signaling molecules [8]. Therefore, in principle, these agents can be developed to target any cellular molecule, regardless of its cellular location. To date, most small-molecule inhibitors have been designed to interfere with enzymes, most notably the receptor tyrosine kinases (RTKs) [9]. Extensive research into small-molecule inhibitors over the last few decades has resulted in several agents receiving Food and Drug Administration (FDA) approval for the treatment of cancer. Some examples, which are discussed in this review, include inhibitors of the tyrosine kinases, human epidermal growth factor receptor 2 (HER2), epidermal growth factor receptor (EGFR) and vascular endothelial growth factor (VEGF), and inhibitors of the serine/threonine kinases, BRAF and Akt. Non-receptor tyrosine kinases (nRTKs) have also been explored as anti-cancer agents. One of the greatest therapeutic success stories to date was the development of the BCR-Abl inhibitor, imatinib, which received FDA approval in 2001 for the treatment of chronic myelogenous leukemia [10]. Imatinib has shown complete hematologic responses in 98% of patients after 60 months of treatment [11]. Other small molecule targets include the ubiquitin proteasome pathway, matrix metalloproteinases (MPPs), heat-shock proteins (HSPs), and the apoptotic proteins p53 and Bcl-2 [12]. To date, the FDA has approved more than 20 small-molecule inhibitors for clinical use in the treatment of cancer.

mAbs are used in the treatment of many diseases, including autoimmune diseases and cancer. These can be recognized by the stem “-mab”, with a further sub-stem designating the source of the compound, e.g., “-mumab” for fully human antibodies. There are several types of mAbs, including naked, conjugated, and biphasic [13, 14]. The most common of these are the naked-mAbs, which do not have an attached drug or radioactive agent. These utilize several different mechanisms, some of which include: targeting the immune system, e.g., alemtuzumab (Campath®, Sanofi, France), which binds CD52 inducing an immune response; targeting antigens on cancer cells that are involved in cell growth and proliferation, e.g., trastuzumab (Herceptin®, Genentech, USA) for HER2; and immune check-point inhibitors, e.g., ipilimumab (Yervoy®, Bristol-Myers Squibb, USA) for cytotoxic T-lymphocyte–associated antigen 4 (CTLA-4). In contrast to this, the conjugated-mAbs have chemotherapy or radioactive particles attached, thereby delivering the toxic substance to the targeted location. Examples include the radiolabeled mAb, ibritumomab tiuxetan (Zevalin®, Biogen, USA) targeted to CD20, which has been used for the treatment of non-Hodgkin lymphoma [15]. The chemo-labeled-mAbs include the anti-CD30 mAb, brentuximab (Adcetris®, Seattle Genetics and Takada), and the anti-HER2 protein attached to the cytotoxic agent DM1, ado-trastuzumab emtasine (TDM-1; Kadcyla®, Genentech) [16, 17]. Lastly, the bispecific-mAbs have two different proteins attached, such as blinatumomab (Blincyto®, Amgen, USA), which binds both CD19 and CD3 [18]. Currently, the FDA has approved over 65 mAbs for cancer treatment, and many more are being studied in clinical trials either alone or in combination with other treatments [19]. In this review, we have discussed some of the more notable mAbs, including those targeting HER2 (Trastuzumab, Pertuzumab), VEGF (cetuximab, bevacizumab), and EGFR (Panitumumab).

One of the hallmarks of cancer is its ability to escape eradication by the immune system [20]. Importantly, there exist two immune checkpoints that are negative regulators of T-cell immune function, these are CTLA-4 and programmed death 1 (PD-1) [21]. New immunotherapies that act to inhibit these checkpoints, resulting in increased activation of the immune system, are now available for the treatment of various cancer types, including melanoma and non–small cell lung cancer (NSCLC). In addition to antagonists of the CTLA-4 and PD-1 pathways, there are other immune checkpoint inhibitors under development that may enhance cytotoxic T-cell activity by antagonizing regulatory pathways that suppress T-cell function [22].

Therefore, there has been significant progress to date in the development of more targeted therapies with the aim of providing greater anti-cancer activity and fewer undesirable side effects. Herein, we discuss the landmark events in the clinical development of these agents.

### Epidermal Growth Factor Receptor (EGFR)

#### Background of targeted therapies to EGFR

**Fig. 1:**
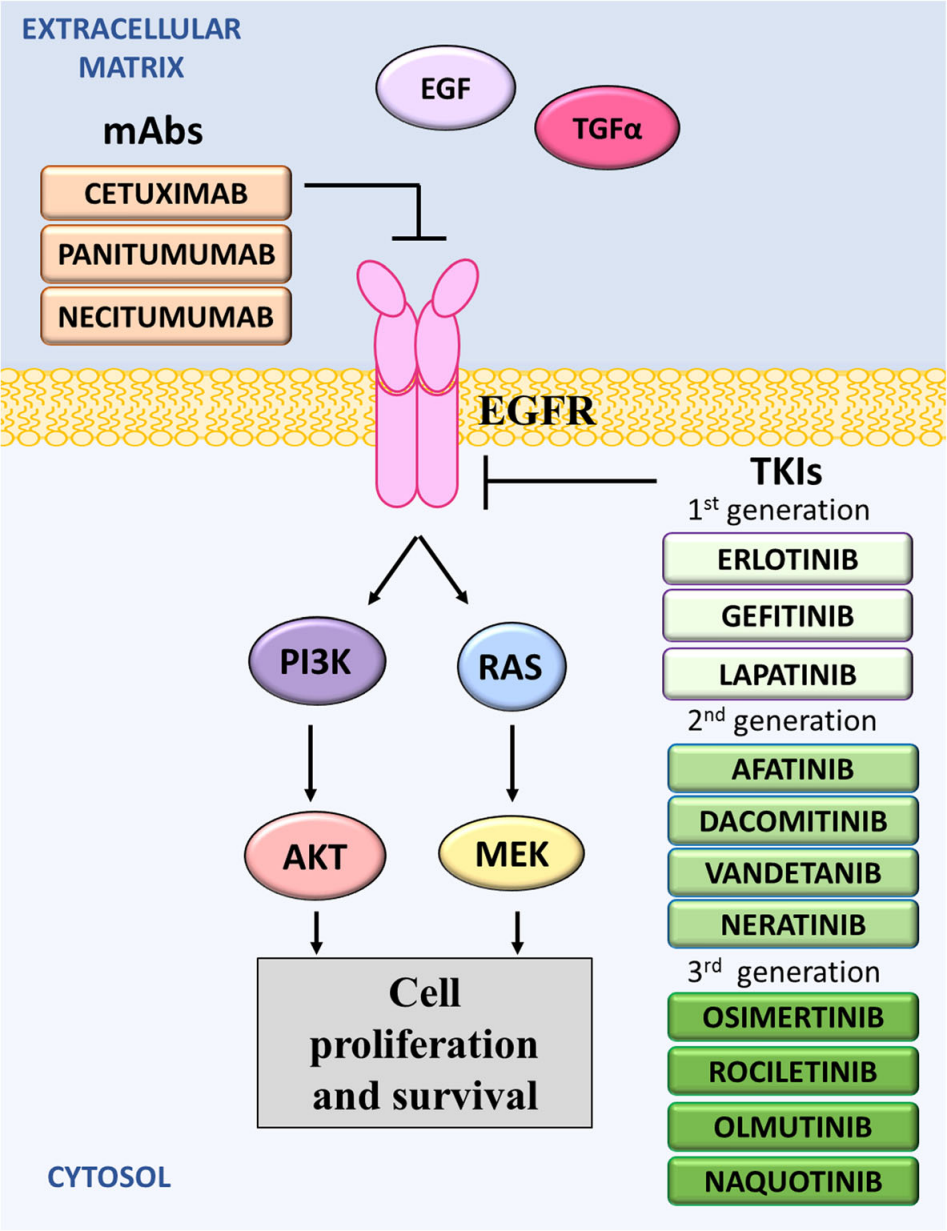
Mechanism of action of anti-EGFR drugs. The activation of EGFR has been implicated in the development of several cancers. There are three generations of tyrosine kinase inhibitors that target the tyrosine kinase of EGFR. Recently the monoclonal antibodies, cetuximab, panitumumab and necitumumab, were developed to target EGFR and thereby prevent the downstream signaling resulting in the proliferation and survival of cancer cells

EGFR is a transmembrane glycoprotein and a member of the ErbB receptor family of tyrosine kinases, which also includes HER2/neu, HER3, and HER4 [23, 24]. Activation of the EGFR receptor occurs following the binding of a specific epidermal growth factor (EGF) ligand, such as EGF or transforming growth factor α (TGFα), which causes a structural change that results in the dimerization of two receptors (Fig. 1) [25–27]. This induces tyrosine phosphorylation by the kinase domains, leading to enhanced, uncontrolled proliferation through downstream signaling.

The EGFR family has been implicated in the development and progression of many cancers, notably NSCLCs, glioblastomas, colorectal cancers (CRCs), breast cancers, and ovarian tumors, through specific driver mutations [28–32]. Most mutations promote receptor dimerization without ligand binding, thereby constitutively activating kinase activity. Notably, kinase domain hotspot mutations, which are often found in NSCLC patients of Eastern Asian origin, frequently have the L858R point mutation [33–36]. In addition to this, EGFR gene amplifications are also common, with studies showing that up to 50% of CRCs and NSCLCs demonstrate a marked increase in EGFR gene copy number [37, 38]. Consequently, these mutations tend to confer inappropriate activation of the downstream, anti-apoptotic Ras signaling cascade, leading to uncontrolled cell proliferation.

Due to the frequent involvement of the ErbB family in cancer, several anti-EGFR therapies have been developed and extensively investigated. These include both the tyrosine kinase inhibitors (TKI), and more recently, monoclonal anti-receptor antibodies. The small-molecule EGFR TKIs compete with Adenosine 5′ triphosphate (ATP) to bind to the intracellular catalytic domain of the EGFR tyrosine kinase, thereby inhibiting EGFR auto-phosphorylation and downstream signaling [39]. In contrast, anti-EGFR mAbs block ligand-induced EGFR tyrosine kinase activation by binding to the extracellular domain of EGFR, thereby competing with ligands for receptor binding [40, 41].

#### Clinical development of small-molecule EGFR tyrosine kinase inhibitors

The first generation of TKIs, gefitinib (ZD1839; Iressa®, AstraZeneca, UK), erlotinib (Tarceva®, Genentech), and lapatinib (TYKERB®, GlaxoSmithKline, UK), are synthetic, low molecular weight anilinoquinazolines (Fig. 1) [42]. Positive results from pre-clinical studies prompted extensive clinical studies in NSCLC patients, which have demonstrated anti-cancer activity against EGFR mutated cancers [43–45].

Gefitinib was the first commercially available inhibitor of the EGFR tyrosine kinase domain. Since its initial introduction into the Japanese market in 2002, gefitinib has since been FDA approved as a first-line treatment for metastatic, EGFR-mutated (exon 19 deletions or exon 21 L858R substitutions) NSCLC [46, 47]. This was based on data from the ‘IPASS’ clinical trials and the follow-up ‘IFUM’ studies, in which gefitinib improved median overall survival (OS; 18.6 vs. 17.3 months), median progression-free survival (PFS; 24.9 vs. 6.7%; *p < 0.001*) and objective response rates (ORR; 43.0 vs. 32.2%; *p < 0.001*), when compared with standard treatment of carboplatin and paclitaxel (Table 1) [48–50]. In fact, results showed that tumors shrank in almost half of all patients after treatment and this effect lasted an average of six months [47]. To date, approval for gefitinib has been granted in over 90 countries. While the anti-tumor activity of gefitinib remains to be fully characterized, it is reported to competitively bind to the intracellular ATP-binding domain of EGFR, thereby inhibiting tyrosine kinase activity [51, 52]. While gefitinib treatment has demonstrated impressive and durable responses in some patients with NSCLC, only very limited activity, if any, has been shown in clinical studies of other cancers expressing high levels of EGFR, including prostate, breast, head and neck, CRC, mesothelioma, brain, kidney, gastric and ovarian cancers [53]. These clinical trials have revealed that, in addition to the common side effects of diarrhea and skin reactions, gefitinib can cause more serious adverse effects, including interstitial lung disease, liver damage, gastrointestinal perforation, severe diarrhea and ocular disorders [54, 55].

**Table 1:** Landmark clinical trials in the development of small-molecule EGFR TKIs

Drug Name	Clinical Trial ID	Trial Name	Population	Comparator	Year	Sponsor	Phase	N	Median OS (months)	Median PFS (months)
Small-molecule EGFR TKIs										
1st Generation EGFR TKI										
Gefitinib (Iressa®/ZD1839)										
Gefitinib (250 mg/d)	NCT00322452	IPASS	NSCLC	Chemotherapy	2006–2010	AstraZeneca	III	1329	18.6 vs 17.3	5.7 vs 5.8/24.9 vs 6.7%
Gefitinib (250 mg/d)	NCT01203917	IFUM	NSCLC (EGFR+)	None	2010–2013	AstraZeneca	IV	1060	19.2	7.0
Erlotinib (Tarceva®)										
Erlotinib (150 mg/d)	NCT00036647	BR.21	NSCLC	Placebo	2001–2004	OSI Pharmaceuticals	III	731	6.7 vs 4.7	2.2 vs 1.8
Erlotinib (150 mg/d)	NCT00556712	SATURN	NSCLC	Placebo	2010–2013	Hoffmann-La Roche	Obs	289	12.4 vs 11.0	12.3 vs 11.1
Erlotinib (150 mg/d)	NCT01328951	IUNO	NSCLC	Placebo	2011–2016	Hoffmann-La Roche	III	643	9.7 vs 9.5	3.0 vs 2.8
Erlotinib (100 mg/d) + Gemcitabine (1000 mg/m^2^/w)	NCT02694536		Pancreatic cancer	None	2006–2009	Hoffmann-La Roche	III	80	7.5	4.9
Lapatinib (Tykerb®)										
Lapatinib (1250 mg/d) + capecitabine (2000 mg/m^2^)	NCT00078572		Breast (HER2+)	Capecitabine	2004–2006	GSK	III	408	17.3 vs 14.9	7.2 vs 4.3
Lapatinib (1500 mg/d) + letrozole (2.5 mg/d)	NCT00073528		Breast (ER/PR +)	Letrozole	2003–2018	Norvatis	III	1285	33.3 vs 32.3	8.1 vs 3.0
Lapatinib (1500 mg/d)	NCT00374322	TEACH	Breast (HER2+)	Placebo	2006–2013	GSK	III	3166	7.3 vs 8.0%	13.3 vs 15.8%
2nd Generation EGFR TKI										
Afatinib (BIBW 2992/Gilotrif®)										
Afatinib (50 mg/d)	NCT00525148	LUX-Lung 2	NSCLC	None	2007–2015	Boehringer Ingelheim	II	129	26.8	10.2
Afatinib (40 mg/d)	NCT00949650	LUX-Lung 3	NSCLC, Adenocarcinoma	Pemetrexed + cisplatin	2009–2017	Boehringer Ingelheim	III	345	28.2 vs 28.2	11.2 vs 6.9
Afatinib (40 mg/d)	NCT01121393	LUX-Lung 6	NSCLC, Adenocarcinoma	Gemcitabine + cisplatin	2010–2017	Boehringer Ingelheim	III	364	23.1 vs 23.5	11.0 vs 5.6
Afatinib (40-50 mg/d)	NCT01523587	LUX-Lung 8	NSCLC	Erlotinib	2012–2017	Boehringer Ingelheim	III	795	NR	2.4 vs 1.9
Afatinib (40 mg/d) + vinorelbine (25 mg/m^2^)	NCT01125566	LUX-Breast 1	Breast (HER2+)	Trastuzumab + vinorelbine	2010–2018	Boehringer Ingelheim	III	508	19.6 vs 28.6	5.5 vs 5.6
Afatinib (40 mg/d)	NCT01271725	LUX-Breast 2	Breast (HER2+)	Afatinib + vinorelbine + paclitaxel	2011–2017	Boehringer Ingelheim	II	74	NR	NR
Afatinib (40 mg/d)	NCT01441596	LUX-Breast 3	Breast (HER2+)	Investigator’s choice	2011–2015	Boehringer Ingelheim	II	121	13.3 vs 12.0	2.7 vs 4.2
Dacomitinib (Vizimpro®)										
Dacomitinib (45 mg/d)	NCT01774721	ARCHER 1050	NSCLC (EGFR mutant)	Gefitinib	2013–2016	SFJ Pharmaceuticals	III	440	16.9 vs 11.9	14.7 vs 9.2
Vandetanib (Caprelsa®)										
Vandetanib (300 mg/d)	NCT00410761	ZETA	Thyroid	Placebo	2006–2019	Sanofi	III	437	13.9 vs 16.0%	30.5 vs 19.2
Vandetanib (300 mg/d)	NCT00409968 NCT00411671 NCT00411632 NCT00410059 NCT00410189	BATTLE Program	NSCLC	Erlotinib, erlotinib + bexarotene, sorafenib	2006–2018	United States Department of Defense	II	255	33.0%	1.8
Neratinib (Nerlynx®)										
Neratinib (240 mg/d)	NCT00878709	ExteNET	Breast Cancer	Placebo	2009–2020 (active)	Puma Biotechnology, Inc.	III	2840	4.7 vs 8.0 (DFS)	NR
3rd Generation EGFR TKI										
Osimertinib (Tagrisso®)										
Osimertinib (80 mg/d)	NCT01802632	AURA extension	NSCLC (EGFR-T790 M)	None	2013–2018	AstraZeneca	I/II	201 [603]	NR	9.7
Osimertinib (80 mg/d)	NCT02094261	AURA 2	NSCLC (EGFR-T790 M)	None	2014–2019	AstraZeneca	II	210	NR	8.6
Osimertinib (80 mg/d)	NCT02151981	AURA 3	NSCLC	Chemotherapy	2014–2018 (active)	AstraZeneca	III	419	NR	10.1 vs 4.4
Rociletinib										
Rociletinib (500–750 mg BD)	NCT01526928		NSCLC	None	2012–2019	Clovis Oncology, Inc.	I/II	605		13.1
Naquotinib										
Naquotinib (dose NR)	NCT02588261	SOLAR	NSCLC	Erlotinib or gefitinib	2016–2017 (terminated)	Astellas Pharma Inc	III	530	NR	NR

Erlotinib**,** like gefitinib, reversibly binds to the ATP-binding site of the EGFR receptor to prevent its activation [56]. Following results of the pivotal Phase III trial ‘BR.21’, erlotinib was first FDA-approved in 2004 for the treatment of locally advanced or metastatic NSCLC following standard treatment failure (Table 1) [57]. In this trial of 731 participants, the median OS was significantly longer in the erlotinib group compared with the placebo group (6.7 vs. 4.7 months; *p < 0.001*) [58]. In 2010, after the ‘SATURN’ Phase III trials, the FDA approved erlotinib as a maintenance treatment for patients with locally advanced or metastatic NSCLC where the disease had not progressed after platinum therapy (Table 1). The ‘SATURN’ trial showed that erlotinib significantly extended median OS (12.4 vs. 11.0 months; *p < 0.01*) and PFS (12.3 vs. 11.1 weeks; *p < 0.0001*) in a broad patient population, including squamous and non-squamous histology, compared with the placebo (Table 1) [59]. Later in 2016, results of the Phase III ‘IUNO’ clinical trial demonstrated that median OS following treatment with erlotinib was no better than the placebo administered as maintenance in patients with metastatic NSCLC tumors not harboring EGFR-activating mutations (Table 1). This led to modification of the indication for erlotinib, limiting treatment to metastatic NSCLC that have specific EGFR mutants, and as a maintenance therapy if there is no progression after platinum based first-line treatment. Erlotinib has also been approved, in combination with gemcitabine, for locally advanced, unresectable, or metastatic, pancreatic cancer based on the median OS, PFS and ORR reported in the Phase III clinical trial, NCT02694536 (Table 1). Erlotinib has a similar side-effect profile to gefitinib, including skin toxicities that typically present as a papulopustular, follicular, or acneiform rash [60].

Lapatinib is slightly different to gefitinib and erlotinib, as it uses a dual mechanism of blocking both the EGFR and HER2/neu pathways [61]. In 2007, success of the Phase III clinical trial, NCT00078572, led to the FDA approval of lapatinib in combination with capecitabine for treatment-naïve, ER+/EGFR+/HER2+ breast cancers (Table 1) [62]. Trial data reported a significant improvement in the median time to disease progression (TTP; 31.3 vs. 18.6 weeks) with the combination of lapatinib and capecitabine compared to capecitabine alone (*p* < 0.001) [62]. Lapatinib has since been FDA approved as a combination treatment with letrozole in HER2+, advanced breast cancer patients that have failed standard chemotherapeutic treatment. This indication was based on clinical trial data where women treated with lapatinib and letrozole experienced a significant 5.2 month increase in median PFS compared to letrozole treatment alone (*p* < 0.05, NCT00073528; Table 1). Similar adverse effects were observed to gefitinib and erlotinib.

However, the success of the first generation TKIs has been limited by acquired resistance, developing at around 12–16 months, mediated mostly by a T790 M missense mutation on exon 20 of EGFR [48, 63, 64]. To overcome resistance to the first generation TKIs, a second generation of EGFR TKIs were developed (Fig. 1) [65, 66]. These include afatinib (Gilotrif®, Boehringer Ingelheim, Germany), dacomitinib (Vizimpro®, Pfizer), vandetanib (ZD6474; Caprelsa®, Sanofi), neratinib (Nerlynx™, Puma Biotechnology, USA), pelitinib (EKB-569) and canertinib (CI-1033). These agents act by irreversibly binding to the EGFR tyrosine kinase [67–76]. Despite promising pre-clinical data, minimal improvement in clinical activity has been found in these agents, with the exception of afatinib and dacomitinib [67, 77–81].

Afatinib is also an anilinequinazoline derivate that binds in a non-competitive, covalent manner with the ATP-binding site of the kinase domain, irreversibly inhibiting EGFR and HER2 [82–84]. Compared with the first generation TKIs, afatinib has demonstrated 100-fold greater binding to T790 M-mutant EGFR cancer cells [82, 85, 86]. Phase III clinical trials in NSCLC patients have demonstrated improvement in ORR and PFS, but not OS compared with placebo or standard chemotherapy treatment [87–90]. These treatment benefits were greatest in EGFR-mutant patients. The FDA has approved afatinib as a first-line treatment for metastatic NSCLC EGFR-mutant cancers, as well as for advanced squamous cell carcinoma of the lung following failure of platinum-based chemotherapy. Approval was based on the clinical trials, ‘LUX-Lung 2’, ‘LUX-Lung 3’, and ‘LUX-Lung 6’, in NSCLC harboring non-resistant EGFR mutations (S768I, L861Q, and/or G719X) and the ‘LUX-Lung 8’ in patients with advanced squamous cell carcinomas of the lung (Table 1). The adverse events arising from afatinib treatment, including rash and diarrhea, appear to be predictable and manageable. Due to its activity against HER2, afatinib has also been investigated in clinical trials for the treatment of HER2+ breast cancers, but has not yet shown any marked improvement in median OS or PFS over other standard treatments (LUX-Breast 1, LUX-Breast 2, and LUX-Breast 3; Table 1) [91].

Dacomitinib is also a selective and irreversible EGFR/HER2 inhibitor [92]. In vitro studies in HER2-amplified breast cancer cell lines and EGFR mutant NSCLC cell lines have demonstrated the strong anti-proliferative activity of dacomitinib, providing a rational for its progression into clinical testing against HER2 positive and EGFR mutant cancers [71, 92]. In September 2018, dacomitinib received its first FDA approval as a first-line treatment of patients with metastatic NSCLC with EGFR exon 19 deletion or exon 21 L858R substitution mutations. This approval was based on data from the ‘ARCHER 1050’ Phase III trial of 440 participants, which reported that dacomitinib, when compared with gefitinib, significantly improved PFS (14.7 vs. 9.2 months) in the first-line treatment of EGFR-mutant NSCLC patients *(p < 0.0001)* [93]. However, this occurred at the cost of greater toxicity to the patients with serious events occurring in 27% of patients (Table 1) [93]. Early phase clinical trials are currently underway to assess dacomitinib for the treatment of skin cancer, HER2+ gastric cancer, head and neck cancer, glioblastomas, and esophageal cancer.

Vandetanib, which targets both EGFR and VEGF, has been FDA approved for the treatment of medullary thyroid cancers in patients with unresectable, locally advanced, or metastatic disease [75]. This occurred following the ‘ZETA’ Phase III clinical trial data demonstrating improvement in PFS (30.5 vs. 19.2 months) compared with the placebo treated controls (Table 1) [94]. The same results have not been seen in clinical trials against small cell lung cancer, metastatic breast cancer, or multiple myeloma. While the ‘BATTLE’ phase II studies have shown that vandetanib prolongs PFS in NSCLC patients, it has not been demonstrated to have improved efficacy compared with erlotinib (Table 1) [95, 96]. A Risk Evaluation and Mitigation Strategy is required for vandetanib due to the risks of QT prolongation, torsades de pointes and sudden death.

Like afatinib and dacomitinib, neratinib is a dual inhibitor of HER2 and EGFR tyrosine kinases [97]. In the large-scale, ‘ExteNET’, Phase III trial of 2840 women with HER2+ breast cancer, neratinib significantly improved 2-year invasive disease-free survival when compared with the placebo treatment *(p < 0.01,* Table 1) [98]. In 2017, neratinib was FDA approved for patients with early-stage HER2+ breast cancer who have finished at least 1 year of post-surgery trastuzumab (Herceptin®, Genentech) therapy. Neratinib has also been assessed in Phase I/II trials as a monotherapy for the treatment of NSCLC patients, but has shown limited benefit [99].

Although these 2nd generation of EGFR TKIs have demonstrated anti-T790 M-EGFR activity, they also irreversibly inhibit wild-type EGFR, causing more severe toxic side-effects [67, 71]. Therefore, a 3rd generation of EGFR-TKIs are in active clinical development to target EGFR-T790 M specifically, while sparing wild-type EGFR (Fig. 1) [100]. The specific targeting of EGFR-T790 M by these agents has limited the toxic side effects of these drugs. These agents include osimertinib (AZD9291/ Tagrisso®; AstraZeneca; formerly mereletinib), rociletinib (CO-1686; Clovis Oncology, USA), olmutinib (HM61713; Hanmi Pharmaceutical, South Korea), naquotinib (ASP8273; Astellas Pharma Inc., Japan), tesevatinib (XL647/KD019; Kadmon Corporation, USA), nazartinib (Novartis, Switzerland; EGF816), and PF-06747775. Trials of these 3rd generation compounds are showing encouraging results, most notably in patients with EGFR-T790 M tumors.

Osimertinib is an irreversible T790 M-EGFR mutant-selective TKI that is also able to bind irreversibly to EGFR that hold a L858R mutation or an exon 19 deletion [101]. More than 50% of NSCLC patients that are EGFR mutation-positive and who have experienced disease progression following EGFR-TKI treatment, have developed a T790 M resistance mutation, for which there has been few treatment options [65, 102]. Following the results of the Phase II ‘AURA2’ and the Phase III ‘AURA3’ clinical trials, in 2015, the FDA accelerated approval of osimertinib for the treatment of EGFR-T790 M mutant NSCLC patients following EGFR-TKI therapy (Table 1). The AURA3 study demonstrated a significant improvement in median PFS (10.1 vs. 4.4 months) with osimertinib compared to the chemotherapy arm *(p < 0.001)*. However, disease progression arises after 10 months of treatment due to the development of resistance mechanisms, including additional mutations in EGFR and activation of alternative kinases [103]. Currently, there are 9 Phase III clinical trials underway to assess osimertinib activity in NSCLC patients.

Rociletinib is also an irreversible mutant-selective inhibitor of commonly mutated forms of EGFR (exon 19 deletion, L858R, and T790 M) that has been assessed in early Phase I-II clinical trials [104]. In these studies, rociletinib was associated with tumor responses and sustained disease control among patients with heavily pretreated EGFR-mutated NSCLC (NCT01526928; Table 1) [105]. Due to its mutation-specific selectivity, rociletinib did not cause the syndrome of rash, stomatitis, and paronychia that is associated with inhibition of non-mutant EGFR. In 2016, following lower response rates than previously reported, the clinical development of rociletinib for the treatment of EGFR-T790 M NSCLC was stopped and all trial enrolments terminated.

Olmutinib is another third generation EGFR TKI that was approved in 2015 as second-line treatment for NSCLC patients in South Korea [106]. However, in 2016, following a case of fatal toxic epidermal necrolysis and Stevens-Johnson Syndrome, Boehringer Ingelheim ended their exclusive licensing deal for olmutinib. It is currently undergoing phase II trials for the treatment of NSCLC in South Korea [106]. Naquotinib has also been assessed for activity against NSCLC with EGFR mutations in the phase III ‘SOLAR’ trial. However, in May 2017, Astellas Pharma discontinued the naquotinib treatment arm following a recommendation by the trial’s Independent Data Monitoring Committee (IDMC; Table 1).

Tesevatinib, nazartinib and PF-06747775 are currently in phase II/III trials to assess their activity against NSCLCs.

#### Clinical development of monoclonal antibodies targeting EGFR

To date, three anti-EGFR mAbs, cetuximab (Erbitux®, Bristol-Myers Squibb/Merck KGaA), panitumumab (ABX-EGF/ Vectibix®, Amgen), and necitumumab (Portrazza®, Eli Lilly and Company, USA), are currently in widespread use in cancer treatment, most notably for CRC. Preclinical assessment of these agents revealed marked anti-tumor activity against EGFR+ cancer cell lines and xenograft models, which prompted their acceleration into clinical trials [107–112].

Cetuximab is a human-murine chimeric anti-EGFR IgG mAb that is currently in use for the treatment of metastatic CRC, metastatic NSCLC, and head and neck cancer. It acts via a number of mechanisms to inhibit EGFR signaling, including; competitively binding the EGF ligand-binding site, thereby preventing dimerization; inducing receptor internalization, downregulation and degradation; inhibiting cell cycle progression through the G_0_/G_1_ phase; and increasing expression of pro-apoptotic proteins [113, 114]. Cetuximab has been evaluated in several phase III clinical trials, including the ‘FLEX’ and ‘ASPECCT’ trials, which have shown a significant median OS and ORR benefit in NSCLCs and CRCs, respectively; although PFS data have been conflicting (Table 2). Cetuximab was first FDA-approved in 2004 for the treatment of advanced metastatic CRC, in combination with irinotecan, in patients who have not responded to irinotecan-based therapy. In 2011, cetuximab was granted approval for the first-line treatment of metastatic head and neck squamous cell carcinomas in combination with cisplatin or carboplatin and 5-fluorouracil. This was based on data from the ‘EXTREME’ clinical trial of cetuximab treatment in head and neck cancer patients, where patients receiving the cetuximab combination therapy had a significantly longer median OS (10.1 vs. 7.4 months; *p < 0.05*) and PFS (5.6 vs. 3.3 months*; p < 0.0001*) compared to those receiving chemotherapy only (Table 2) [115]. In 2012, cetuximab was approved for use in combination with folinic acid, fluorouracil and irinotecan (FOLFIRI) for first the hyphenate all first-line treatment of patients with wild-type Kirsten rat sarcoma viral oncogene homolog (KRAS), EGFR+ metastatic CRC, following results of the large Phase III ‘CRYSTAL’ clinical trial (Table 2). The ‘CRYSTAL’ and ‘OPUS’ clinical trials have highlighted that cetuximab efficacy is limited to patients with wild-type KRAS tumors [116–118]. KRAS is a small G-protein that lies downstream of EGFR and is an essential part of the EGFR signaling cascade [119]. Cancers may acquire activating mutations in exon 2 of KRAS, thus isolating the signaling pathway from the effect of upstream EGFR2, rendering the EGFR inhibitors ineffective. Indeed, the mutation status of KRAS in CRCs is predictive of the patient’s response to therapy [120]. Therefore, it is essential that KRAS status is considered when selecting candidates for cetuximab therapy.

**Table 2:** Landmark clinical trials in the development of monoclonal antibodies targeting EGFR

Drug Name	Clinical Trial ID	Trial Name	Population	Comparator	Year	Sponsor	Phase	N	Median OS (months)	Median PFS (months)
Monoclonal antibodies to EGFR										
Cetuximab (Erbitux®)										
Cetuximab (400 mg/m^2^ initial + 250 mg/m^2^/week) + cisplatin + vinorelbine	NCT00148798	FLEX	NSCLC	Cisplatin + vinorelbine	2005–2014	Merck KGaA	III	1861	11.3 vs 10.1	4.8 vs 4.8
Cetuximab (400 mg/m^2^ initial + 250 mg/m^2^/week)	NCT01001377	ASPECCT	Metastatic CRC	Panitumumab	2010–2017	Amgen	III	1010	10.0 vs 10.4	4.4 vs 4.1
Cetuximab [400/250 mg/m^2^ (initial/weekly)] + Chemotherapy	NCT00122460	EXTREME	H&N Cancer	Chemotherapy	2004–2011	Merck KGaA	III	442	10.1 vs 7.4	5.6 vs 3.3
Cetuximab [400/200 mg/m^2^ (initial/weekly)] + FOLFIRI	NCT00154102	CRYSTAL	Metastatic CRC (KRAS WT)	FOLFIRI	2004–2011	Merck KGaA	III	1221	23.5 vs 20.0	9.9 vs 8.4
Cetuximab + 5-FU/FA + oxaliplatin (FOLFOX-4)	NCT00125034	OPUS	Metastatic CRC (KRAS WT)	5-FU/FA + oxaliplatin	2005–2010	Merck KGaA	II	344	22.8 vs 18.5	8.3 vs 7.2
Panitumumab (Vectibix®)										
Panitumumab (6 mg/kg/2w) + FOLFOX	NCT00364013	PRIME	Metastatic CRC (WT KRAS)	FOLFOX	2006–2013	Amgen	III	1183	23.9 vs 19.7	9.6 vs 8.0
Panitumumab (6 mg/kg/2w) + FOLFOX	NCT00364013	PRIME	Metastatic CRC (Mutant KRAS)	FOLFOX	2006–2013	Amgen	III	1183	15.5 vs 19.3	7.3 vs 8.8
Panitumumab (6 mg/kg/2w) + BSC	NCT01412957	‘0007	Metastatic CRC (WT RAS)	BSC	2011–2017	Amgen	III	377	10.0 vs 6.9	5.2 vs 1.7
Necitumumab (Portrazza®)										
Necitumumab (800 mg/ m^2^/3w) + gemcitabine + cisplatin	NCT00981058	SQUIRE	NSCLC	Gemcitabine + cisplatin	2010–2018	Eli Lilly and Company	III	1093	11.5 vs 9.9	5.7 vs 5.5
Necitumumab (500 mg/m^2^/3w) + Chemotherapy	NCT00982111	INSPIRE	NSCLC	Chemotherapy	2009–2018	Eli Lilly and Company	III	633	11.3 vs 11.5	5.6 vs 5.6

Panitumumab, a fully human monoclonal IgG2 antibody, first gained FDA approval in 2006 for the treatment of EGFR+ metastatic CRC following fluoropyrimidine, oxaliplatin, and irinotecan treatment failure [121]. This approval was based on the success of the ‘PRIME’ Phase III trials, which reported a significant benefit in median PFS (9.6 vs. 8.0 months; *p < 0.05*). Later in 2014, the improvement in the median PRS and OS from panitumumab treatment in the ‘PRIME’ and ‘ASPECCT’ Phase III trials, led to the FDA approval of panitumumab for the first-line treatment of patients with wild-type KRAS (exon 2) metastatic CRC, in combination with oxaliplatin (Table 2). In 2017, panitumumab was also approved for the treatment of patients with wild-type Ras metastatic CRC, as a first-line therapy in combination with folinic acid, fluorouracil, oxaliplatin (FOLFOX), and as a monotherapy following failure of fluoropyrimidine, oxaliplatin, and irinotecan-containing chemotherapy. This approval was based on a retrospective analysis of the ‘PRIME’ study and the Phase III ‘0007 study, which showed a statistically significant improvement in median OS (10.0 vs. 6.9 months; *p < 0.05*) and PFS (5.2 vs. 1.7; *p < 0.0001*) in patients with wild-type-RAS CRC (Table 2). Therefore, like cetuximab, panitumumab monotherapy efficacy in mutant CRC is limited to patients with wild-type KRAS tumors [118].

Necitumumab is a recombinant human IgG1 mAb, which received FDA approval in 2015, for use with gemcitabine and cisplatin against previously untreated, advanced metastatic squamous NSCLC. This approval was based on data from the ‘SQUIRE’ clinical trial, which demonstrated that necitumumab, in combination with gemcitabine and cisplatin, significantly increases median OS (11.5 and 9.9; *p < 0.05*) and PFS (5.7 vs. 5.5; *p < 0.05)* compared with chemotherapy alone (Table 2). The most common side effects reported are rashes and hypomagnesemia, of which the latter can be potentially fatal [122]. Another Phase III clinical trial, ‘INSPIRE’, which assessed necitumumab in combination with pemetrexed and cisplatin for the treatment of non-squamous NSCLC in 633 participants, did not demonstrate any clinical benefit compared with pemetrexed and cisplatin alone (Table 2) [123]. Therefore, necitumumab is currently not indicated for the treatment of non-squamous NSCLC.

## Conclusion

The development of small-molecule inhibitors and mAbs for the targeted treatment of EGFR+ cancers has been an exciting area of research in recent years. Their specificity and toxicity have improved the prognosis of patients with NSCLC, CRC, pancreatic cancer, breast cancers and squamous cell carcinoma of the head and neck. Indeed, we have seen a number of these agents become standard of care for cancer treatment e.g., cetuximab. Over the next few decades, we can expect to see further optimization of antibody structures and more effective treatments with the implementation of newer genotype-targeted personalized therapies. Gaining the full benefits of anti-EGFR strategies requires further investigations to identify if there are other specific mutations, in addition to the T790 M mutation, which can be targeted.

### Vascular Endothelial Growth Factor (VEGF)

#### Background of targeted therapies to VEGF

VEGF is a glycoprotein that is a widely-known regulator of angiogenesis [124–127]. It is required for the cellular process of wound healing, embryonic vasculogenesis and vascular permeability [124]. The VEGF family consists of 5 members: VEGFA, VEGFB, VEGFC, VEGFD and placenta growth factor 1 (PGF1) [128]. All members of the VEGF family are involved in vessel angiogenesis [128–130].

VEGF is important for tumor growth as solid tumors rely on angiogenesis for the supply of oxygen and nutrients to aid growth, and as a route for invasion and metastasis [124]. In fact, without adequate vasculature, many solid tumors will not grow beyond 2 mm^3^ [131, 132].

Overexpression of VEGF has been correlated with advanced tumor stages and invasiveness and is, therefore, a target for cancer therapeutics [125]. Mutations in oncogenes, such as *ras* or *p53,* and the inhibition of several tumor suppressor genes, such as *PTEN* or *WT1*, can result in the up-regulation of VEGF [126, 133–135].

#### Clinical development of VEGF inhibitors

**Fig. 2:**
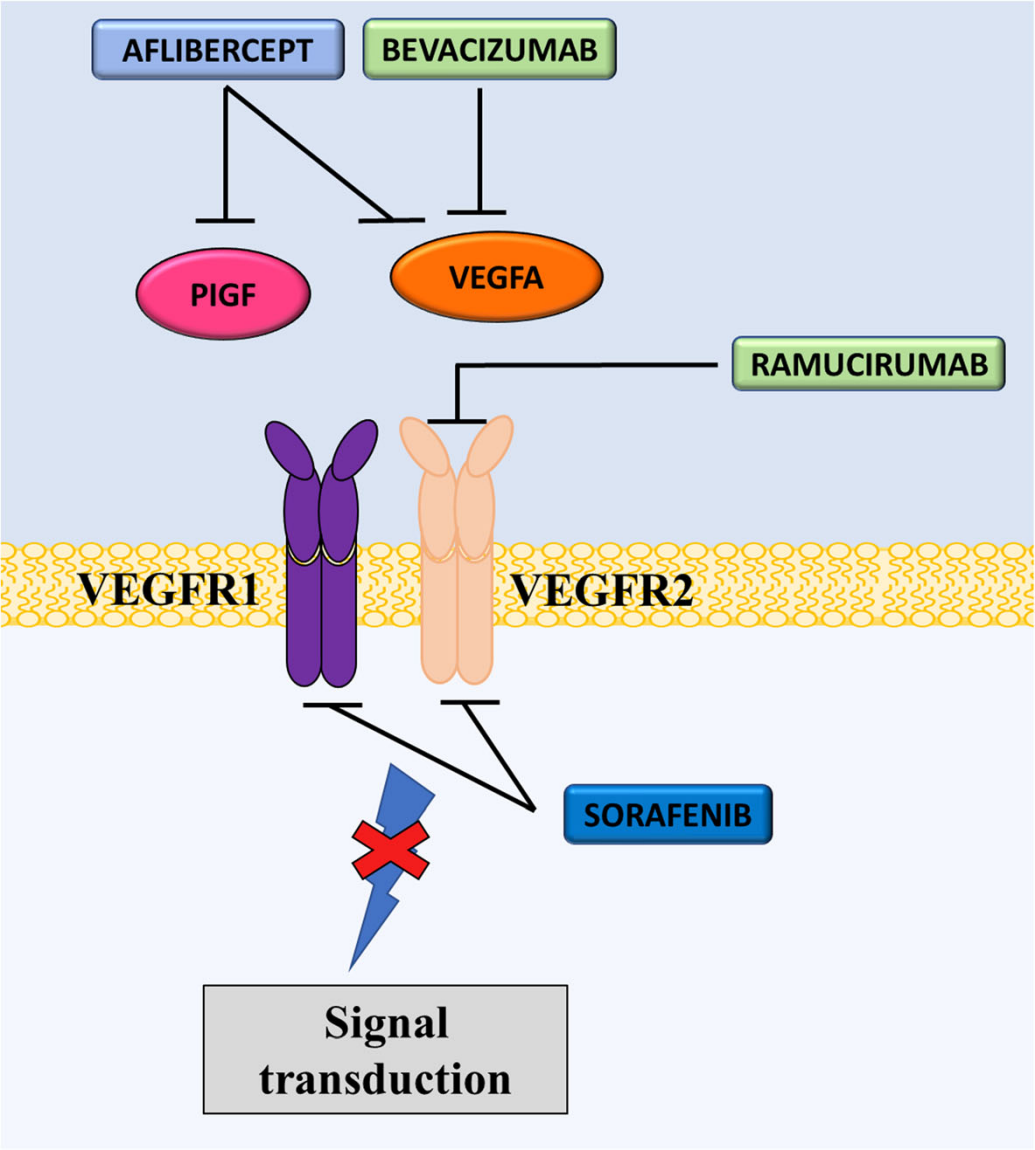
Mechanism of action of anti-VEGF/VEGFR drugs. Due to activation of VEGF signaling pathways in various cancers, several anti-cancer drugs have been developed to target the VEGF pathway. Aflibercept is a peptide-antibody directed at PIGF and VEGFA, while bevacizumab is a mAb specific for VEGFA. Ramucirumab is a mAb that targets the VEGFA receptor (VEGFR2). On the other hand, sorafenib is a tyrosine kinase inhibitor for VEGFR, primarily VEGFR2. Each of these drugs prevent oncogenic signaling by VEGF overexpression

Blockage of the VEGF/VEGF receptor (VEGFR) signaling pathways, through mAbs, ligand inhibitors and TKIs, has shown to be clinically beneficial in several cancers including, but not limited to, CRC, breast cancer and lung cancer [125, 136–138]. For example, sorafenib (Nexavar®, Bayer and Onyx Pharmaceuticals, Germany) is a multi-TKI for VEGFR1, VEGFR2, VEGFR3, platelet derived growth factor receptor (PDGFR), FMS-like tyrosine kinase 3 (Flt-3), c-Kit protein (c-Kit) and RET RTKs (Fig. 2) [139]. This agent has shown single agent efficacy against renal cell carcinoma (RCC) in the ‘TARGET’ Phase III trials [139]. Furthermore, oral sorafenib significantly prolonged median OS (542 vs. 436 days; *p < 0.05*) and PFS (167 vs. 84 days; *p < 0.000001*) in patients with hepatocellular carcinoma (HCC) compared with placebo (Table 3) [140]. Although the drug was associated with an increased number of side effects, such as hypertension, PFS was improved in clear-cell RCC patients whose first-line therapy had failed [139]. Accordingly, sorafenib was approved for the treatment of RCC and HCC. Furthermore, in 2013, sorafenib was FDA approved for the treatment of metastatic differentiated thyroid cancer [141]. FDA approval was based on the significant improvement in median PFS (329 vs. 175 days; *p < 0.0001*) observed in a Phase III double-blind placebo-controlled trial of 417 patients with differentiated thyroid carcinomas (NCT00984282; Table 3). However, patients experienced significant toxicities, including hand-foot skin reactions, diarrhea, and asthenia [142]. The mechanism behind sorafenib-induced toxicities is not clear and may involve disruptions of multiple signaling pathways in healthy organs, including VEGF, PDGF, RAF1, BRAF, KIT, and FLT3 [143–146].

**Table 3:** Landmark clinical trials in the development of VEGF inhibitors

Drug Name	Clinical Trial ID	Trial Name	Population	Comparator	Year	Sponsor	Phase	N	Median OS (months)	Median PFS (months)
VEGF inhibitors										
Sorafenib (Nexavar®)										
Sorafenib (400 mg BD)	NCT00073307	TARGET	Metastatic RCC	Placebo	2003–2006	Bayer	III	903	17.8 vs 15.2	5.5 vs 2.8
Sorafenib (400 mg BD)	NCT00984282		Thyroid cancer	Placebo	2009–2012	Bayer	III	417	52.7 vs 54.8%	10.8 vs 5.8
Bevacizumab (Avastin®)										
Bevacizumab (10 mg/kg/2w)	NCT00281697	RIBBON 2	Metastatic Breast Cancer	Placebo	2006–2009	Genentech	III	684	18.6 vs 17.8	7.2 vs 5.1
Bevacizumab (5 mg/kg/w)	NCT00528567	BEATRICE	Breast cancer (triple negative)	Standard adjuvant chemotherapy	2007–2012	Hoffmann-La Roche	III	2591	NR	NR
Bevacizumab (10 mg/kg/2w)	NCT00028990	E2100	Metastatic breast cancer	Paclitaxel	2001–2006	Eastern Cooperative Oncology Group	III	722	NR	11.8 vs 5.9
Bevacizumab (5 mg/kg/w)	NCT01169558		Metastatic CRC	Combination with Fluoropyrimidine-based Chemotherapy	2006–2009	Hoffmann-La Roche	III	162	21.6	11.0
Bevacizumab (15 mg/kg/3w)	NCT01239732		Ovarian cancer	Paclitaxel + Carboplatin	2010–2015	Hoffmann-La Roche	III	1021	NA	25.5
Bevacizumab (dose NR) + chemotherapy	NCT00565851	GOG-0213	Ovarian, Epithelial, Peritoneal, Fallopian Tube Cancer	Chemotherapy	2007–2019	National Cancer Institute	III	1038	42.2 vs 37.3	13.8 s 10.4
Bevacizumab (15 mg/kg/3w) + chemotherapy	NCT00434642	OCEANS	Ovarian cancer	Chemotherapy	2007–2013	Genentech	III	484	33.6 vs 32.9	12.4 vs 8.4
Bevacizumab (10 mg/kg/w) + IFNα2A	NCT00738530	AVOREN	RCC	IFNα2A	2004–2008	Hoffmann-La Roche	III	649	23.3 vs 21.3	10.2 vs 5.5
Bevacizumab (15 mg/kg/3w) + chemotherapy	NCT00803062	GOG-240	Cervical cancer	Chemotherapy	2008–2017	National Cancer Institute	III	452	17.5 vs 14.3	9.6 vs 6.7
Bevacizumab (10 mg/kg)	NCT00345163	BRAIN	Glioblastoma	Chemotherapy	2006–2007	Genentech	II	167	8.7 vs 9.2	50.3 vs 42.6%
Bevacizumab (10 mg/kg)	NCT01351415		NSCLC	Chemotherapy	2006–2014	Hoffmann-La Roche	III	485	11.9 vs 10.2	5.5 vs 4.0
Ramucirumab (Cryamza®)										
Ramucirumab (8 mg/kg/2w)	NCT00917384	REGARD	Metastatic gastric or gastroesophageal junction cancer	Placebo	2009–2015	Eli Lilly and Company	III	355	2.1 vs 1.3	5.2 vs 3.8
Aflibercept (EYLEA®)										
Aflibercept (4 mg/kg) + FOLFIRI	NCT00561470	VELOUR	CRC	FOLFIRI	2007–2012	Sanofi	III	1226	13.5 vs 12.1	6.9 vs 4.7
Aflibercept (4 mg/kg) + docetaxel	NCT00532155	VITAL	Metastatic NSLC	Docetaxel	2007–2011	Sanofi	III	913	10.1 vs 10.4	5.2 vs 4.1
Aflibercept (4 mg/kg) + gemcitabine	NCT00574275	VANILLA	Metastatic pancreatic cancer	Gemcitabine	2007–2010	Sanofi	III	546	7.8 vs 6.5	3.7 vs 3.7

Recent decades have seen the introduction of mAbs for the treatment of cancer [147]. Currently, there is one clinically approved mAb targeting VEGF used in oncology, which is known as bevacizumab (Avastin®, Genentech) (Fig. 2) [147]. Bevacizumab was developed in 1997 by the humanization of murine anti-VEGF mAb [126, 127]. The agent specifically binds to and neutralizes VEGFA, although its exact mechanisms of action are not fully elucidated [148].

Studies by Willis et al. (2004) demonstrated that VEGF blockade by bevacizumab resulted in a reduction of vascular volume, reduced tumor perfusion and reduced interstitial pressure [149]. Therefore, bevacizumab may result in the remodeling of tumor vasculature, reducing its density and increasing the organization and efficient network of vessels [131, 149]. It was proposed that this allows for more effective delivery of chemotherapy and, because of this, bevacizumab can be combined with chemotherapy to maximize clinical outcomes [131]. Furthermore, bevacizumab was shown to have apoptotic effects on tumor cells [150, 151]. As VEGF can provide survival signals to tumor cells, it is likely that VEGF blockade induces apoptosis [150]. Studies in lung carcinoma cells showed that the drug was able to induce apoptosis of the tumor cells by causing endoplasmic reticulum stress [151]. Findings in colon cancer cells also demonstrated the occurrence of hypoxia-induced apoptosis by bevacizumab [152].

A number of clinical trials have demonstrated that bevacizumab has activity against cancers of the breast [153], lung [154], colon [155], brain [156] and kidney [150, 155, 157]. In Phase I trials, the drug was well tolerated and did not exhibit dose-limiting toxicity [154, 158]. Numerous clinical trials demonstrated that the combination of bevacizumab with various chemotherapeutics, including paclitaxel or doxorubicin or fluorouracil and leucovorin, resulted in a statistically significant improvement in median OS and PFS in CRC, ovarian and lung cancer patients (Table 3) [157–160]. Following its success in clinical trials, bevacizumab is currently approved for the treatment of CRC (NCT01169558), glioblastoma (NCT00345163), ovarian cancer (GOG-0213, OCEANS, NCT01239732), renal cancer (AVOREN), breast cancer (E2100, BEATRICE) and cervical cancer (GOG-240). Therefore, bevacizumab is an important drug that has the potential to be useful over a wide variety of cancers due to the prevalence of VEGF overexpression in solid tumors [124].

The clinical effectiveness of bevacizumab led to the development of several other agents that target the VEGF pathway. For example, ramucirumab (Cyramza®, Eli Lilly) is a humanized mAb that acts as an antagonist to VEGFR2, thereby preventing the VEGF ligand binding and inhibiting downstream effects (Fig. 2) [161]. This receptor mediates the main angiogenic response after VEGF binding [162]. Some Phase II trials demonstrated that ramucirumab did not alter PFS [161]. However, there were some promising results when used in combination with chemotherapeutics, such as paclitaxel or docetaxel, and the agent is now approved for treatment of gastro-esophageal, CRC and lung cancer [162–164]. The pivotal ‘REGARD’ Phase III trial showed that monotherapy with ramucirumab significantly reduced the risk of disease progression by half (median PFS = 2.1 vs. 1.3; *p < 0.0001*) and improved median OS (5.2 vs. 3.8 months; *p < 0.05*) when compared with placebo (Table 3) [161]. Several other Phase III clinical trials are underway with promising results attesting to the clinical benefits of targeting the VEGF pathway. Further trials are required in order to determine toxicity profiles in combination with other chemotherapeutics [165].

Aflibercept, or VEGF-Trap, is a peptide-antibody that targets VEGFA, VEGFB and PIGF (Fig. 2) [166]. The drug can bind to and ‘trap’ these proteins, preventing them from causing downstream angiogenic effects [167]. So far, there have been 8 completed Phase III clinical trials using aflibercept for the treatment of cancer [168–175]. However, there are currently no FDA approvals for the use of aflibercept against cancer. The ‘VELOUR’ Phase III clinical trial in CRC showed that aflibercept, in combination with FOLFIRI, conferred a statistically significant benefit in patient median OS (13.5 vs. 12.1 months; *p < 0.01*) and median PFS (6.9 vs. 4.7 months; *p < 0.0001*) when compared with the chemotherapeutics alone (Table 3) [166]. Similarly, data from Phase III ‘VITAL’ trial showed that aflibercept in combination with docetaxel significantly improvement median PFS (5.2 vs. 4.1 months; *p < 0.01*) in metastatic NSCLC patients compared with docetaxel alone (Table 3). However, the Phase III ‘VANILLA’ clinical trials, examining the combination of aflibercept and gemcitabine in advanced pancreatic cancer, showed there was no significant improvement in median OS or PFS, compared with gemcitabine alone (Table 3) [176].

## Conclusion

Targeting the VEGF pathway has shown clinical importance in cancer therapy with the development of TKIs against VEGFR and, importantly, mAbs against VEGF. Along with the successes of bevacizumab, ramucirumab and aflibercept, it is important to note that these agents possess various limitations. For example, bevacizumab was withdrawn by the FDA for the treatment of metastatic breast cancer in 2011 because it was unable to show PFS in subsequent clinical trials [177]. Nevertheless, the VEGF signaling pathway remains an important target of cancer therapeutics. Further understanding the mechanisms of these drugs is essential to improving the treatment of cancer patients.

### Human Epidermal Growth Factor Receptor (HER2)

#### Background of targeted therapies to HER2

HER2 is a transmembrane tyrosine kinase receptor involved in cell growth, survival, adhesion, migration and differentiation [178]. HER2 is a member of the HER family that consists of HER1, 2, 3 and 4 [179]. HER2 is activated in response to homodimerization and heterodimerization with other EGFR proteins [180]. Activation results in the initiation of a number of signaling pathways involved in survival and proliferation such as the mitogen-activated protein kinase (MAPK) pathway, the phosphoinositide-3-kinase (PI3K/Akt) pathway and the protein kinase C (PKC) pathway [179]. HER2-overexpression has been documented in several human malignancies and is present in 20–30% of invasive breast cancers [181, 182]. HER2-overexpression can result in dimerization and constitutive activation of survival and proliferation signaling pathways [183]. Further evidence suggests that HER2 overexpression may result in disruptions to cell adhesion and loss of cell polarity [179]. Patients with HER2-overexpressing breast cancer have poorer responses to chemotherapeutics and hormonal therapies [184]. Considering this, studies have focused on targeting HER2 as a therapeutic approach.

#### Clinical development of HER2 inhibitors

**Fig. 3:**
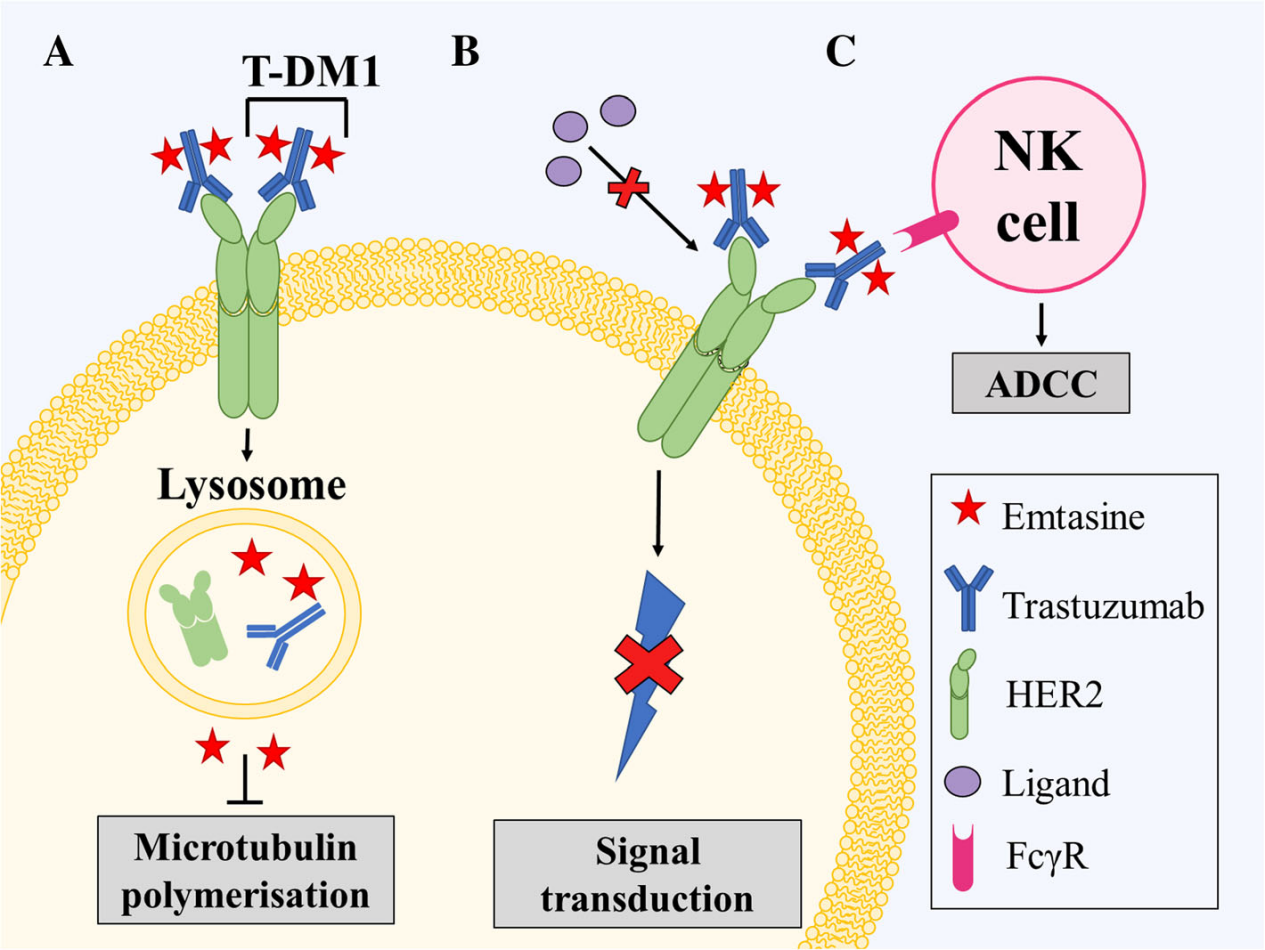
Mechanism of action of trastuzumab emtansine (T-DM1). T-DM1 binds via Fc receptors to the Human Epidermal Growth Factor Receptor 2 (HER2) on the cell membrane. This agent has three main mechanisms of action. **a** The T-DM1/HER2 complex is internalized by endosomes and subsequently degraded within lysosomes, releasing emtansine. Emtansine then binds to microtubules and inhibits polymerization. **b** T-DM1 also inhibits downstream signaling of HER2 by preventing ligand binding and **c** induces antibody-dependent cell-mediated cytotoxicity (ADCC) where natural killer (NK) cells bind to the immune complex (consisting of T-DM1 bound to surface-expressing HER2) through Fc gamma receptors (FcγR) and kill the tumor cell

One such strategy was the development of an antibody specific for HER2, namely, trastuzumab (Herceptin®, Genentech) [183]. The antigen binding portion of this antibody was first developed in mice and was then fused with human IgG to reduce immunogenicity in patients [185]. Trastuzumab was approved by the FDA in 1998 for treatment of HER2-overexpressing breast cancer [180]. The success of trastuzumab led to the development of further antibodies, such as pertuzumab (Omnitarg™, 2C4, Genentech), and the antibody-drug conjugate (ADC) trastuzumab-emtansine (T-DM1; Fig. 3).

Considering that trastuzumab is an antibody, it is likely that one mechanism of action of this agent may be the recruitment of immune cells and subsequent antibody-dependent cellular cytotoxicity (ADCC) [186]. This was demonstrated by Arnould et al. (2006) who used immunohistochemical analysis to assess the presence of immune cells in breast cancer tissue [186]. These studies showed that the addition of trastuzumab to chemotherapy resulted in an increase in natural killer cells, other immune cells and cytotoxic proteins (such as Granzyme B) in tumor infiltrates [186]. Moreover, this study showed that HER2 expression on tumor cells was unaffected by trastuzumab, which suggests that ADCC is a major contributor to the anti-cancer activity of the drug [186]. Further evidence for trastuzumab-mediated ADCC was demonstrated by Clynes et al. (2000) using mouse xenograft models [187]. These studies established that natural killer cells were able to target cells coated in trastuzumab bound to the over-expressed HER2 [187]. It is well characterized that HER2 activation results in the activation of the MAPK and the PI3K/Akt pathways, which, in turn, results in increased cell growth and proliferation [180]. Trastuzumab prevents this activation by binding to HER2 and inhibiting the dimerization of this latter protein [188]. Therefore, trastuzumab treatment prevents the constitutive activation of these pathways caused by overexpression of HER2 and thereby prevents growth and proliferation of cells [188].

Trastuzumab has undergone several clinical trials in which optimal doses, toxicity and patient outcomes were measured (Table 4) [180, 189, 190]. One such important clinical trial determined the effect of trastuzumab in combination with various chemotherapies (i.e., anthracycline, cyclophosphamide, doxorubicin and/or epirubicin) for patients with HER2-overexpressing breast cancer [182]. This Phase III clinical trial consisted of 469 patients with HER2-overexpressing metastatic breast cancer who had not previously received chemotherapy [182]. The results of this trial showed that combination therapy resulted in a 20% reduction in risk of death at 30 months [182]. In fact, time to disease progression increased from 4.6 months (chemotherapy alone group) to 7.4 months (combination therapy group; Table 4) [182]. Unfortunately, trastuzumab induced some cardiotoxic side effects whereby 63 patients out of 469 experienced symptomatic or asymptomatic cardiac dysfunction [182]. The highest proportion of patients with cardiotoxicity were those that also received anthracycline and cyclophosphamide, consequently, the authors cautioned the use of trastuzumab in patients that had previously received these agents [182].

**Table 4:** Landmark clinical trials in the development of HER2 inhibitors

Drug Name	Clinical Trial ID	Trial Name	Population	Comparator	Year	Sponsor	Phase	N	Median OS (months)	Median PFS (months)
HER2 inhibitors										
Trastuzumab (Herceptin®)										
Trastuzumab (4 mg/kg followed by 2 mg/kg) + doxorubicin + cyclophosphamide	NCT00004067		Breast cancer (HER2+)	Doxorubicin + cyclophosphamide + paclitaxel	2000–2020	NSABP Foundation Inc	3	42,130	NA	NA
Trastuzumab (8 mg/kg followed by 6 mg/kg) + chemotherapy	NCT01998906		Breast cancer (HER2+)	Chemotherapy	2002–2012	Hoffmann-La Roche	3	330	NA	NA
Trastuzumab (4 mg/kg followed by 2 mg/kg) + docetaxel	Marty et al. (2005)	M77001	Breast cancer (HER2+)	Docetaxel	2000–2005	Hoffmann-La Roche	2	186	31.2 vs 22.7	11.7 vs 6.1
Trastuzumab (4 mg/kg followed by 2 mg/kg) + lapatinib	NCT00320385		Breast cancer (HER2+)	Lapatinib	2005–2010	GlaxoSmithKline	3	296	51.6 vs 39 (weeks)	12 vs 8.1 (weeks)
Trastuzumab (8 mg/kg followed by 6 mg/kg) + fluorouracil + cisplatin + capecitabine	NCT01041404	ToGA Study	HER2+ advanced gastric cancer	Fluorouracil + Cisplatin + Capecitabine	2005–2010	Hoffmann-La Roche	3	584	11.1 vs 13.8	5.5 vs 6.7
Trastuzumab (4 mg/kg followed by 2 mg/kg) + chemotherapy	NCT00021255		Breast cancer (HER2+)	Chemotherapy	2001–2014	Sanofi	3	3222	78.9 vs 86	NA
Trastuzumab (2 mg/kg i.v. weekly, or 6 mg/kg i.v. every 3 weeks) + chemotherapy	NCT00448279	THOR	Breast cancer (HER2+)	Chemotherapy	2007–2010	Hoffmann-La Roche	3	58	19.1 vs 26.7	9.7 vs 9.4
T-DM1 (Trastuzumab Emtansine/ Kadcyla®)										
T-DM1 (3.6 mg/kg/3w)	NCT00829166	EMILIA	Breast cancer (HER2+)	Lapatinib + Capecitabine	2009–2015	Hoffmann-La Roche	III	991	30.9 vs 25.1	9.6 vs 6.4
T-DM1 (3.6 mg/kg/3w)	NCT01419197	TH3RESA	Breast cancer (HER2+)	Physician’s choice	2011–2015	Hoffmann-La Roche	III	602	22.7 vs 15.8	6.2 vs 3.3
Pertuzumab (Perjeta®)										
Pertuzumab (420 mg/3w) + trastuzumab + docetaxel	NCT00567190	CLEOPATRA	Breast cancer (HER2+)	Trastuzumab and Docetaxel	2008–2018	Hoffmann-La Roche	III	808	56.5 vs 40.8	18.7 vs 12.4
Pertuzumab (420 mg/3w) + trastuzumab + capecitabine	NCT01026142	PHEREXA	Breast cancer (HER2+)	Trastuzumab + capecitabine	2010–2017	Hoffmann-La Roche	III	452	37.2 vs 28.1	11.1 vs 9.0
Pertuzumab (420 mg/3w) + trastuzumab + chemotherapy	NCT01358877	APHINITY	Breast cancer (HER2+)	Trastuzumab + chemotherapy	2011–2016	Hoffmann-La Roche	III	4804	NR	8.7 vs 7.1%
Pertuzumab + T-DM1	NCT01120184	MARIANNE	Breast cancer (HER2+)	T-DM1 + Placebo	2010–2016	Hoffmann-La Roche	III	1095	51.8 vs 53.7	15.2 vs 14.1
Lapatinib (Tykerb®)										
Lapatinib (1250 mg/d) + capecitabine	NCT00078572		Metastatic breast cancer (HER2+)	Capecitabine	2004–2010	GSK	III	408	10.4 vs 8.0	8.4 vs 4.4
Lapatinib (1500 mg/d)	NCT00073528		Metastatic breast cancer	Letrozole	2003–2018	Novartis	III	1285	33.3 vs 32.3	8.1 vs 3.0
Lapatinib (1500 mg/d)	NCT00374322	TEACH	Early stage breast cancer	Placebo	2006–2013	GSK	III	3166	NR	NR

The current standard of care for HER2+ breast cancer patients begins with standard adjuvant treatment with chemotherapy and trastuzumab, which significantly improves survival [191]. In 2015, a clinical trial showed that HER2+ breast cancer patients that were not administered anti-HER2+ therapy had an ongoing risk of recurrence [191]. Trastuzumab has shown clinical importance, although its complete mechanisms of action remain elusive [184].

Despite the promise of trastuzumab, some patients experienced disease progression and other patients developed resistance to trastuzumab [192]. This led to the development of T-DM1 [193]. T-DM1 is an ADC that consists of the drug DM1 (a tubulin inhibitor) bound to trastuzumab [194]. ADCs are a novel class of anti-cancer drugs, which have demonstrated marked toxicity and specificity for solid tumors [192, 193]. Studies using T-DM1 demonstrated a double-punch mechanism, by which trastuzumab allowed selective delivery of DM1 to HER2-overexpressing cells while retaining its ability to induce ADCC and inhibition of HER2 signaling [193, 194]. T-DM1 is therefore able to bind to HER2-overexpressing cells and is internalized by the cell where the tubulin inhibitor is released (Fig. 3) [194]. T-DM1 was shown to be effective in HER2-overexpressing tumors in patients who had developed trastuzumab resistance [192]. Clinical trials of the drug have shown that T-DM1 has low toxicity and can be used in combination with lapatinib and nab-paclitaxel for significant anti-tumor activity and, is therefore, a promising drug candidate for HER2-overexpressing breast cancer (Table 4) [195]. In fact, the drug was approved for the treatment of HER2+ metastatic breast cancer after the pivotal Phase III ‘EMILIA’ trial demonstrated significant improvements to patient median PFS (9.6 vs. 6.4 months; *p < 0.0001*) and OS (30.9 vs. 25.1 months; *p < 0.001*) [196, 197]. Unfortunately, not all patients improved with T-DM1 with approximately 15% relapsing due to acquired resistance to the antibody [198]. Similar results were obtained in the ‘TH3RESA’ Phase III clinical trials. Therefore, development of additional HER2 directed antibodies were considered.

Pertuzumab is another humanized mAb against HER2 [199]. It binds to a different epitope of HER2 than trastuzumab that inhibits HER2 dimerization [199]. Pertuzumab was well tolerated in clinical trials and, although its anti-tumor activity was low when used as a monotherapy, it has shown promising effects when given in combination with trastuzumab (Table 4) [198]. For example, the clinical trial ‘APHINITY’ comparing the combination of pertuzumab, trastuzumab and docetaxel with the combination of placebo, trastuzumab and docetaxel showed significantly prolonged median PFS (8.7 vs. 7.1%; *p < 0.05*) and no increase in cardiotoxic events in the pertuzumab combination group [200]. Similarly, the ‘CLEOPATRA’, and ‘PHEREXA’ trials have shown improvements in median PFS (18.7 vs. 12.4; 11.1 vs. 9.0 months, respectively) and OS (56.5 vs. 40.8; 37.2 vs. 28.1 months) when pertuzumab was combined to trastuzumab and chemotherapy compared with trastuzumab and chemotherapy alone (Table 4). Following this, pertuzumab was FDA approved for the treatment of HER2+ early breast cancers at high risk of recurrence.

Considering breast cancer may develop resistance to trastuzumab [201], lapatinib (Tykerb®, GlaxoSmithKline) was developed as an alternative agent to block HER2 signaling pathways. Lapatinib inhibits the tyrosine kinases of HER2 and EGFR and is currently FDA approved for the treatment of breast cancer patients [202]. This agent prevents phosphorylation and activation of the receptors, resulting in inhibition of cell proliferation and induction of apoptosis in vitro [202]. Lapatinib is approved for the treatment of advanced, metastatic HER2+ breast cancer in combination with capecitabine when the tumor has progressed with standard treatment (including trastuzumab) [203]. The FDA approval was based on the Phase III clinical trials, NCT00078572 and NCT00073528. NCT00078572 showed that the median time to disease progression was 27.1 weeks on the combination of lapatinib and capecitabine vs. 18.6 weeks on capecitabine alone in women with advanced or metastatic HER2+ breast cancer whose disease had progressed following treatment with trastuzumab and other cancer therapies (Table 4) [204]. In the NCT00073528, double-blinded, placebo-controlled study, women with HR+ and HER2+ metastatic breast cancer (diagnosed post-menopause) treated with lapatinib and letrozole experienced a significant 5.1 month increase in median PFS compared to women treated with letrozole alone (*p < 0.05*, Table 4).

## Conclusion

HER2 overexpression is seen in a significant proportion of breast cancers and it confers poor survival. Several agents have been developed against HER2 to prevent the pathogenesis involved in this overexpression. Importantly, trastuzumab, T-DM1, pertuzumab and lapatinib have shown clinical importance in the treatment of HER2 overexpressing breast cancer and the application of these drugs have shown significant improvement in patient outcomes. Further investigations into the mechanisms of these drugs and the development of resistance will be crucial to optimize treatment strategies and combinations of HER2 inhibitors.

### Anaplastic lymphoma kinase (ALK)

#### Background of targeted therapies to ALK

The *ALK* gene encodes a RTK that is involved in neuronal development during embryogenesis before becoming dormant [205]. In general, ALK activates multiple signaling pathways, such as the PI3K-AKT, CRKL-C3G, MEKK2/3-MEK5-ERK5, JAK/STAT and MAPK pathways [206]. In cancer, translocations involving the *ALK* gene form nearly 30 different fusion oncogenes [205]. The protein products of these fusion oncogenes exhibit altered spatial and temporal regulation, deregulating multiple signaling pathways and driving tumorigenesis [206]. ALK alterations have been found in several cancers, such as anaplastic large cell lymphoma, NSCLC, inflammatory myofibroblastic tumor, diffuse large B-cell lymphomas, esophageal squamous cell carcinoma, renal medulla carcinoma, RCC, breast cancer, colon carcinoma, serous ovarian carcinoma, and anaplastic thyroid carcinoma [205]. Each fusion protein is associated with specific subtypes of cancer. For example, the most prevalent ALK mutation, the echinoderm microtubule-associated protein-like 4 (EML4)-ALK fusion, is found in approximately 3–13% of NSCLC patients [205, 207–209]. ALK has proved an attractive and clinically successful drug target. Of the 10 small-molecule ALK inhibitors undergoing clinical trials, 4 have gained FDA approval, to date [210].

**Fig. 4:**
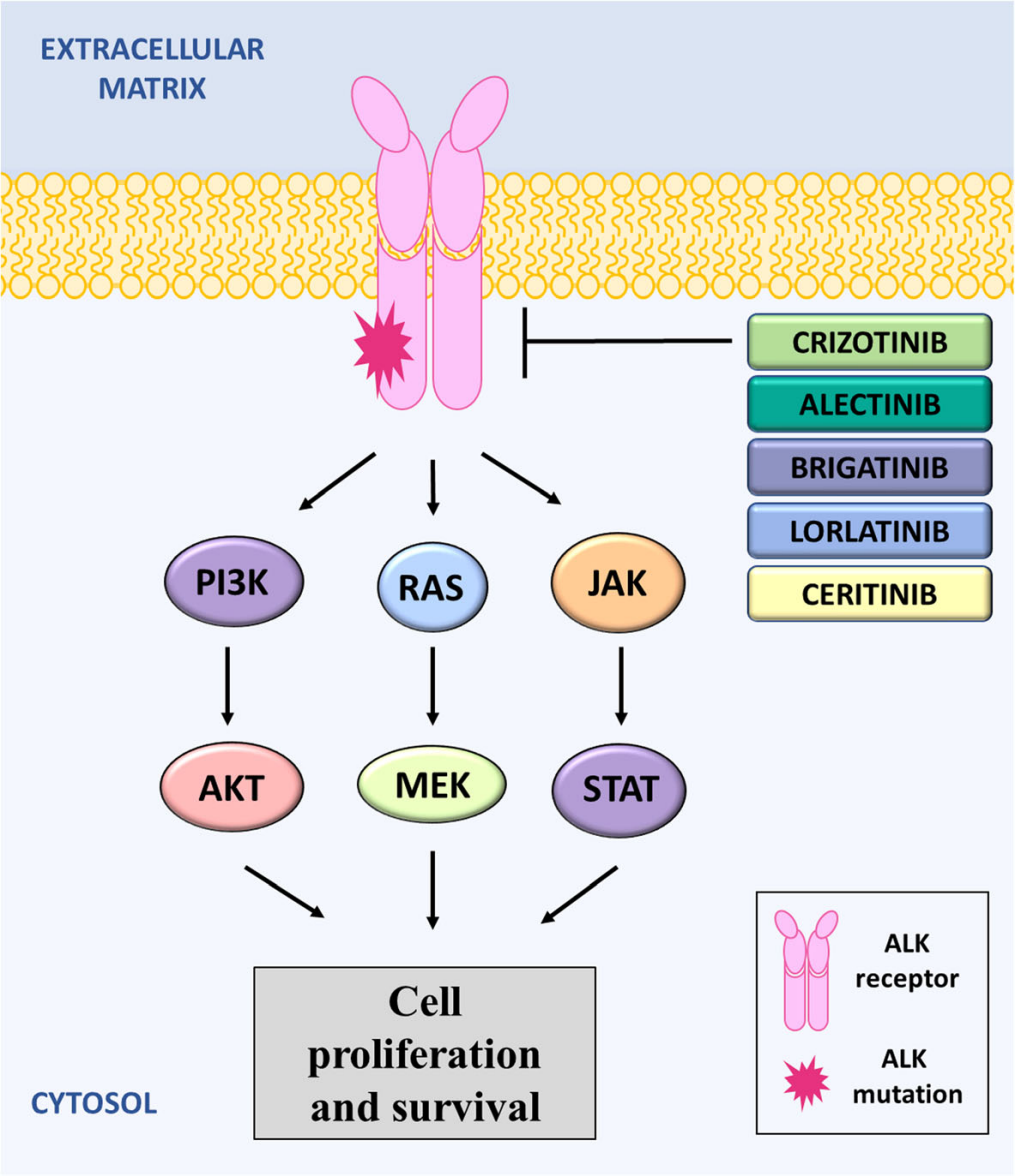
Mechanism of action of ALK inhibitors. ALK activates various signaling pathways involved in cell proliferation and survival, including the PI3K pathway, the RAS/MEK pathway and the JAK/STAT pathway. ALK inhibitors have similar mechanisms of action by binding to the ATP-binding site and blocking activation of ALK. Crizotinib was the first ALK inhibitor approved by the FDA but, unfortunately, resistance to Crizotinib commonly occurs due to mutations the *ALK* gene. Therefore, Ceritinib, Alectinib, Brigatinib and Lorlatinib were developed, and can be used for patients who are not responding to Crizotinib

All current FDA-approved ALK inhibitors exhibit a similar mechanism of action (Fig. 4). By binding to the ATP-binding site of ALK when it is in its active conformation, ALK inhibitors block increased activation of the tyrosine kinase induced by the formation of fusion oncogenes [211–214]. Inhibiting the activation of ALK thus inhibits downstream physiological signaling pathways that induce cell proliferation, cell survival and tumorigenesis.

#### Clinical development of ALK inhibitors

Three generations of ALK inhibitors have been developed and have revolutionized the treatment of advanced ALK-positive patients. These include: the first-generation ALK inhibitor, crizotinib (Xalkori®, formerly PF-02341066, Pfizer); the second-generation inhibitors, ceritinib (Zykadia®, formerly LDK378; Novartis), alectinib (Alcensa®, formerly RO5424802/CH5424802, Hoffmann-La Roche, Inc./Genentech, Inc.), and brigatinib (Alunbrig™, formerly AP26113, Takeda Pharmaceutical Company, Ltd); and the third-generation inhibitor, lorlatinib (PF-06463922; Pfizer; Fig. 4).

Crizotinib was the first ALK inhibitor to gain FDA approval in 2011, as a second-line treatment of *ALK-*positive NSCLC, following treatment failure with platinum-containing chemotherapy. This was due to the success of Phase I, ‘PROFILE 1001’ [215], and Phase II, ‘PROFILE 1005’ [216], trials’ which demonstrated ORRs of 60.8 and 59.8%, and median PFS of 9.7 and 8.1 months, respectively [215, 216]. Phase III results from the ‘PROFILE 1007’ trial confirmed significantly higher response rates and median PFS with crizotinib (65% and 7.7 months, respectively), compared to standard chemotherapy (20% and 3.0 months, respectively; Table 5) [217]. Furthermore, the ‘PROFILE 1014’ trial showed crizotinib to be superior, compared to standard first-line platinum/pemetrexed chemotherapy in patients with untreated, advanced, NSCLC; for which it is now an approved treatment [218]. Crizotinib is generally well-tolerated, with common adverse events including gastrointestinal upset, visual disturbances and hepatotoxicity [215–218]. However, case reports of significant adverse events include erythema multiforme, acute interstitial lung disease, renal polycytosis, and decreased glomerular filtration rate [219].

**Table 5:** Landmark clinical trials in the development of ALK inhibitors

Drug Name	Clinical Trial ID	Trial Name	Population	Comparator	Year	Sponsor	Phase	N	Median OS (months)	Median PFS (months)
ALK inhibitors										
1st Generation ALK-inhibitors										
Crizotinib (Xalkori®)										
Crizotinib (50–2000 mg/d)	NCT00585195	PROFILE 1001	Advanced cancer	Rifampin, Itraconazole	2006–2023	Pfizer	I	600	NR	9.7
Crizotinib (250 mg BD)	NCT00932451	PROFILE 1005	NSCLC	None	2010–2015	Pfizer	II	1069	21.8	8.1
Crizotinib (250 mg BD)	NCT0093283	PROFILE 1007	NSCLC	Permetrexed or docetaxel	NR	Pfizer	III	172	20.3 vs 22.8	7.7 vs 3.0
Crizotinib (250 mg BD)	NCT01154140	PROFILE 1014	Non-squamous lung cancer	Platinum + permetrexed	2011–2013	Pfizer	III	343	NR	10.9 vs 7.0
Ceritinib (Zykadia®)										
Ceritinib (750 mg/d)	NCT01283516	ASCEND-1	Tumors (ALK+)	None	2011–2013	Novartis	I	304	16.7	7.0
Ceritinib (750 mg/d)	NCT02336451	ASCEND-2	NSCLC	None	2015–2018	Novartis	II	160	NR	5.7
Ceritinib (750 mg/d)	NCT01685138	ASCEND-3	NSCLC	None	2008–2018	Novartis	II	125	NR	10.8
Ceritinib (750 mg/d)	NCT01828099	ASCEND-4	NSCLC	Chemotherapy	2013–2016	Novartis	III	375	NR	16.6 vs 8.1
Ceritinib (750 mg/d)	NCT01828112	ASCEND-5	NSCLC	Chemotherapy	2013–2017	Novartis	III	232	20.1 vs 18.1	5.4 vs 1.6
Ceritinib (750 mg/d)	NCT02299505	ASCEND-8	NSCLC	None	2015–2016	Novartis	I	318	NR	NR
Alectinib (Alcensa®)										
Alectinib (600 mg BD)	NCT01871805	NP28761	NSCLC	None	2013–2017	Hoffmann-La Roche	I/II	134	27.9	8.2
Alectinib (600 mg BD)	NCT01801111	NP28673	NSCLC	None	2013–2014	Hoffmann-La Roche	I/II	138	12.1	7.5
Alectinib (600 mg BD)	NCT02075840	ALEX	NSCLC	Crizotinib	2014–2017	Hoffmann-La Roche	III	303	NR	25.7 vs 10.4
Brigatinib (Alunbrig™)										
Brigatinib (90 mg/d)	NCT01449461		NSCLC	None	2011–2015	Ariad	I/II	137	NR	16.3
Brigatinib (90 mg/d)	NCT02094573		NSCLC	None	2014–2016	Ariad	II	222	46%	9.2
Brigatinib (90 mg/d)	NCT02737501	ALTA-L1	NSCLC	Crizotinib	2016–2020	Ariad	III	275	85 vs 86%	67 vs 43%
Lorlatinib										
Lorlatinib (10-200 mg/d)	NCT01970865	CROWN	NSCLC	None	2014–2017	Pfizer	II	367	22.3	5.3

Unfortunately, the majority of patients acquire resistance following crizotinib treatment within 1 to 2 years [220]. Commonly, patients that relapse following crizotinib present with CNS progression [221]. Secondary resistance has been attributed to point mutations in the *ALK* gene, gene amplification, and modification of downstream signaling pathways to bypass ALK inhibition [222–224]. Resistance to crizotinib has led to the development of more potent and selective ALK inhibitors, detailed below.

Ceritinib, which is approximately 20-times more potent than crizotinib, was the next ALK inhibitor to be granted accelerated FDA approval in 2014 [225]. Following a Phase I trial ‘ASCEND-1’ demonstrating an ORR of 60%, and a median PFS of 7.0 months, ceritinib was approved for treatment of relapsed or refractory *ALK*-positive NSCLC, following crizotinib treatment (Table 5) [226]. Importantly, ceritinib treatment resulted in a 56% response rate in patients who had previously been treated with crizotinib, indicating that ceritinib is active in patients with and without acquired resistance mutations [226]. Similar positive results were found in Phase II (ASCEND-2 [226, 227] and ASCEND-3 [228]) and Phase III trials (ASCEND-4 and ASCEND-5) (Table 5) [229, 230]. The results of ‘ASCEND-4’ led to approval of ceritinib as first-line therapy for patients with metastatic NSCLC, whose tumors are ALK+. Gastrointestinal side effects have hindered the use of ceritinib, although a recent trial ‘ASCEND-8’ found that reducing the dose and taking ceritinib with food could reduce adverse events (Table 5) [231].

Alectinib was developed as a more selective and potent ALK inhibitor, exhibiting a three-fold increase in ALK inhibition in vitro [232]. This agent initially received accelerated FDA approval in 2015 for treatment of patients with *ALK*+ metastatic NSCLC whose disease progressed on, or who were intolerant of, crizotinib. Phase I/II trials had demonstrated that alectinib was effective in patients who had previously been treated with an ALK inhibitor, and was effective against central nervous system metastases, unlike crizotinib [233, 234]. Following the results of the Phase III ‘ALEX’ trial, which demonstrated the superior efficacy and lower toxicity of alectinib, compared to crizotinib, this was upgraded to regular approval, in 2017, for treatment-naive patients with *ALK*+ metastatic NSCLC [235]. In the ‘ALEX’ trial, the 12-month event-free survival rate was 68.4% with alectinib, compared to 48.7% with crizotinib (Table 5) [235]. This may reflect the greatest advantage of alectinib treatment over crizotinib, in that the rate of CNS progression is significantly lower. Only 12% of patients treated with alectinib developed CNS progression, compared with 45% of those treated with crizotinib [235]. Additionally, grade 3–5 adverse events occurred in 41% of patients treated with alectinib, compared to 50% treated with crizotinib [235].

Brigatinib, like alectinib and ceritinib, was granted accelerated FDA approval, in 2017, for treatment of patients with *ALK*+ metastatic NSCLC, whose disease progressed on or who were intolerant of crizotinib. The results of the Phase II ‘ALTA’ trial showed an ORR of 54% (Table 5) [214]. This is similar to the ORR for alectinib and ceritinib, however the median PFS of brigatinib was far superior at 12.9 months, compared to 5.7–6.0 months for ceritinib and 8.1–8.9 months for alectinib [214, 230, 235]. Gastrointestinal side effects were common and relatively mild, although severe pulmonary toxicity was largely responsible for the 3.7% fatal event rate. The Phase III (ALTA-1 L) trial is ongoing and scheduled to end in 2020.

Lorlatinib, a third generation ALK-inhibitor, was designed to inhibit *ALK* resistant mutants and penetrate the blood brain barrier (BBB). Like other ALK inhibitors, lorlatinib was granted Breakthrough Therapy Designation from the FDA, in April 2017. This followed successful Phase I/II trials (NCT01970865) demonstrating a 66.4% ORR and 59.4% intracranial ORR, in patients who had previously been treated with ALK inhibitors [236]. In addition, 90% of patients who received lorlatinib as a first-line therapy had a confirmed ORR [236]. The Phase III ‘CROWN’ trial, comparing first-line crizotinib to first-line lorlatinib, is ongoing with an estimated completion date in 2023. Unlike other *ALK* inhibitors for which the main side effects were hepatotoxicity and gastrointestinal upset, common adverse effects of lorlatinib included hypercholesterolemia (72%), hypertriglyceridemia (39%), peripheral neuropathy (39%), and peripheral edema (39%) [236].

## Conclusions

Since the discovery of the *ALK* gene in patients with NSCLC, several ALK-targeted drugs have moved rapidly from the bench to the bedside, and many others are currently under investigation in clinical trials. This has led to important improvements in patient outcomes. However, the emergence of resistance to ALK-directed therapy has presented in the clinic and is now central to ongoing research.

### BRAF

#### *Background of targeted therapies to BRAF*

*BRAF* is a proto-oncogene that encodes the serine/threonine-protein kinase, BRAF (or B-Raf) [237–239]. BRAF is part of the fibrosarcoma kinase (RAF) family of kinases that are key signaling molecules, which form the intermediate between membrane-bound Ras GTPases and the MEK/ERK pathway [237–239]. ERK has been shown to regulate cell proliferation by acting at several levels to increase the activity of the cyclin D and Cdk4/6 complex, which allows cell-cycle progression from the G_1_ to S phase [240]. Therefore, BRAF plays an integral role in regulating cell proliferation in response to growth signals.

The Raf kinases have long been associated with cancer [241]. BRAF mutations have been extensively reported in numerous cancers, including melanomas (50–66%), papillary thyroid tumors (45–50%), CRCs (10%), prostate tumors (10%), and NSCLCs (3%) [238, 242–245]. Studies have reported a V600E hotspot mutation in malignant melanomas and CRCs which increases BRAF kinase activity [242, 246–248]. This mutation represents about 70–90% of all BRAF mutations [242, 249–251]. Moreover, activating mutations of the *BRAF* oncogene are reported in approximately 5–10% of all human malignancies, leading to constitutive activation of the MAPK pathway [242]. These BRAF mutant cancers have been associated with poor patient prognosis [252]. Consequently, agents have been developed to target these mutant cancers.

#### Clinical development of small-molecule BRAF tyrosine kinase inhibitors

**Fig. 5:**
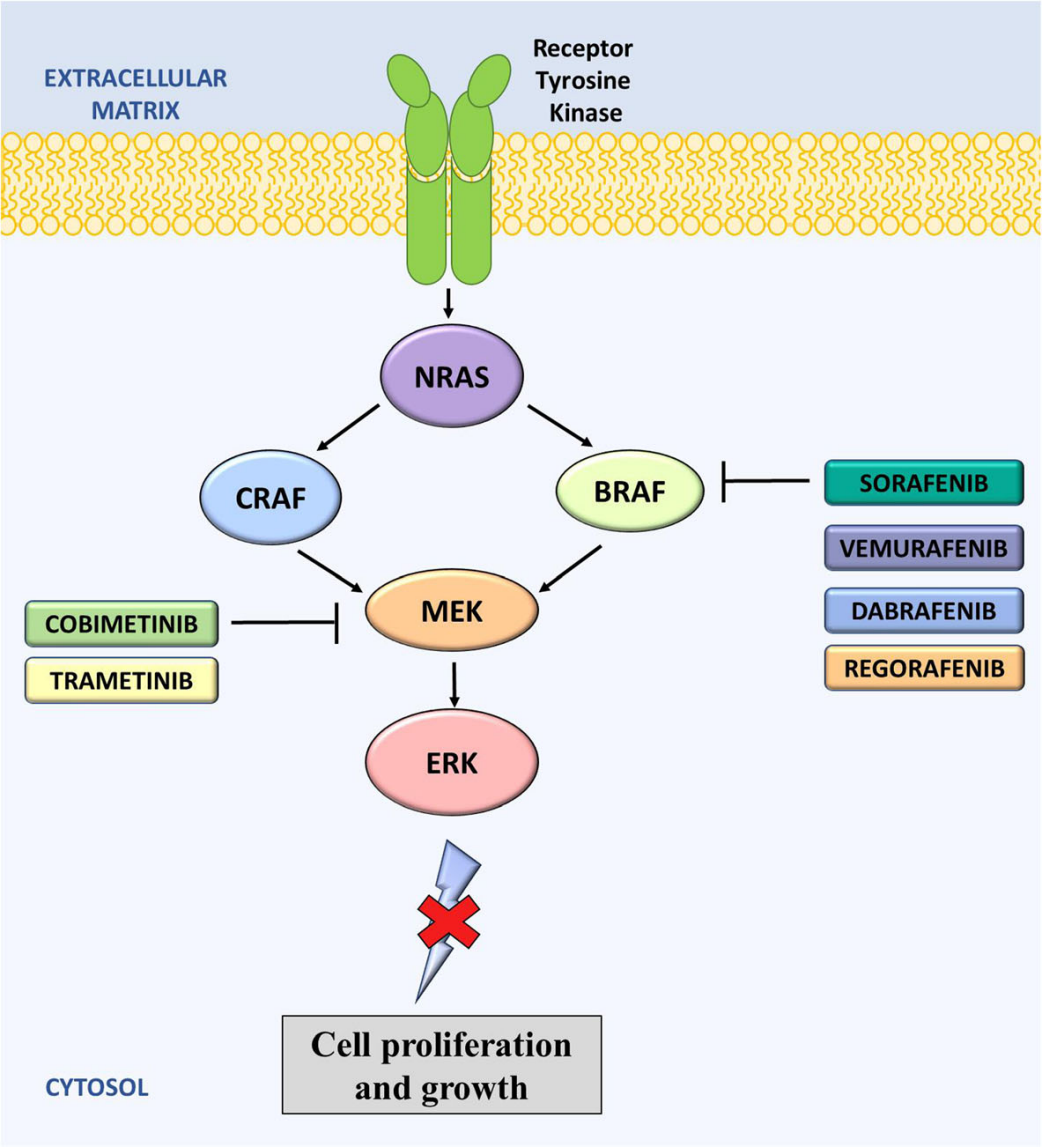
Mechanism of action of anti-BRAF drugs on the RAS signaling pathway. RAS activates both the CRAF and the BRAF pathways. Inhibitors for both BRAF and MEK are shown. These inhibitors act to prevent cell proliferation and growth of cancer cells. Sorafenib, vemurafenib, dabrafenib, cobimetinib, regorafenib, and trametinib are all FDA approved for the treatment of cancer

To date, all agents that have been developed to target BRAF are small molecule kinase inhibitors (Fig. 5). These can be divided into two types: type I inhibitors, which bind in an active conformation, and type II inhibitors, which bind to the protein kinase in an inactive conformation [253]. The type I agents are reportedly more specific inhibitors and show greater response rates when compared with the type II inhibitors [253].

Sorafenib, a type I, multi-target TKI, in addition to its anti-VEGF activity (see VEGF Section), also acts to inhibit BRAF by binding to the ATP binding site of the kinase domain of the inactive enzyme [254, 255]. Sorafenib was the first RAF inhibitor to enter clinical trials, which occurred prior to the discovery of BRAF mutations in cancer. Molecular characterization studies of NSCLC and HCC lesions has since revealed a BRAF exon 11 mutation (G469 V) that may be responsible in part for some of the observed sensitivity to sorafenib [256]. The results of this study highlighted a role for sorafenib in BRAF-mutated tumors. However, when sorafenib was studied in Phase II trials for the treatment of melanoma, no relationship between V600E BRAF status and disease stability was observed (Table 6) [257]. Following the success of numerous clinical trials, sorafenib is now FDA approved for the treatment of RCC, hepatocellular (SHARP) and thyroid cancers (NCT00984282). Interestingly, it remains unclear which RAF, if any, is the predominant therapeutic target of sorafenib. Efficacy in RCCs is likely due to inhibition of VEGFR2, and, although responses in HCC are correlated with ERK phosphorylation, responses are not correlated with RAS mutational status [258].

**Table 6:** Landmark clinical trials in the development of BRAF inhibitors

Drug Name	Clinical Trial ID	Trial Name	Population	Comparator	Year	Sponsor	Phase	N	Median OS (months)	Median PFS (months)
BRAF inhibitors										
Sorafenib (Nexavar®)										
Sorafenib (400 mg BD)	NCT00105443	SHARP	HCC	Placebo	2005–2008	Bayer	III	602	10.8 vs 8.0	5.5 vs 2.8
Sorafenib (800 mg)	NCT00984282		Thyroid	Placebo	2009–2017)	Bayer	III	417	52.7 vs 54.8%	10.8 vs 5.8
Sorafenib (400 mg BD)	NCT00119249		Melanoma		2005–2007	NCI	II	74	NR	NR
Vemurafenib (Zelboraf®)										
Vemurafenib (960 mg BD)	NCT01910181	BRIM	Metastatic melanoma	None	2013–2018	Hoffmann-La Roche	I	46	13.5	8.6
Vemurafenib (960 mg BD)	NCT00949702	BRIM2	Melanoma	None	2009–2014	Hoffmann-La Roche	II	132	NA	6.1
Vemurafenib (960 mg BD)	NCT01006980	BRIM3	Metastatic melanoma	Dacarbazine	2010–2015	Hoffmann-La Roche	III	675	13.6 vs 9.7	NR
Dabrafenib (Tafinlar®)										
Dabrafenib (150 mg BD)	NCT01153763	BREAK-2	Melanoma	None	2010–2016	GSK	II	92	3.0	1.4
Dabrafenib (150 mg BD)	NCT01227889	BREAK-3	Melanoma	Dacarbazine	2010–2014	GSK	III	251	20.0 vs 15.6	6.7 vs 2.9
Dabrafenib (150 mg BD) + trametinib	NCT01336634		NSCLC	Dabrafenib	2011–2015	Norvatis	II	174	18.2 vs 12.7	10.2 vs 5.5
Dabrafenib (150 mg BD)	NCT01723202		Thyroid	Trametinib	2012–2018	National Comprehensive Cancer Network	II	53	NR	NR
Regorafenib (Stivarga®)										
Regorafenib (160 mg/d)	NCT01103323	CORRECT	Colorectal cancer	Placebo + BSC	2010–2014	Bayer	III	760	6.4 vs 5.0	1.9 vs 1.7
Regorafenib (160 mg/d)	NCT01271712	GRID	GIST	Placebo	2011–2012	Bayer	III	199	2.7 vs 2.6	4.8 vs 0.9
Regorafenib (160 mg/d)	NCT01774344	RESORCE	HCC	Placebo	2013–2017	Bayer	III	573	10.6 vs 7.8	3.6 vs 1.5
Cobimetinib (Cotellic®)										
Cobimetinib (60 mg/d) + vemurafenib	NCT01689519	coBRIM	Melanoma	Vemurafenib + Placebo	2012–2015	Hoffmann-La Roche	III	495	22.3 vs 17.4	9.9 vs 6.2
Trametinib (Mekinist®)										
Trametinib (2 mg/d) + dabrafenib	NCT01682083	COMBI-AD	Melanoma	Placebo	2013–2017	Norvatis	III	870	NR	NR
Trametinib (2 mg/d) + dabrafenib	NCT02034110		Thyroid		2014–2020	Norvatis	II	100	80%	79%

Unfortunately, sorafenib has not only demonstrated minimal efficacy in BRAF-mutated melanomas but has had significant side effects [259]. Recently, two new type II BRAF inhibitors, vemurafenib (PLX4032/ Zelboraf**®,** Plexxikon and Genentech) and dabrafenib **(**GSK2118437/ Tafinlar®, GlaxoSmithKline), have achieved approval by the FDA for the treatment of metastatic and unresectable BRAF-mutated melanomas [241, 260].

Vemurafenib is a potent small-molecule inhibitor of BRAF V600E among other BRAF mutations [261–263]. The FDA approved vemurafenib for the treatment of patients with mutant-V600E BRAF metastatic melanomas in 2011. This was based on results from the Phase I, II and III clinical studies (‘BRIM1’, ‘BRIM2’ and ‘BRIM3’, respectively) in people with BRAF V600E mutation-positive, inoperable or metastatic melanomas (Table 6). In these studies, melanoma patients bearing mutant-V600E BRAF had partial or complete response rates to vemurafenib between 48 and 81% with the median PFS extending beyond 7 months. Unfortunately, approximately 24% of patients had detectable cutaneous squamous cell carcinomas (SCCs) and keratoacanthomas as a side effect of this treatment. In November 2017, the FDA also granted approval for the use of vemurafenib in Erdheim-Chester Disease, a rare type of histiocytic neoplasm, with BRAF V600 mutations.

Dabrafenib is also an extremely potent inhibitor of V600E-mutated BRAF, which has shown efficacy in melanoma and CRC both in vitro and in vivo [264, 265]. In addition to V600E, dabrafenib also has demonstrated activity against V600D+ and V600R+ cancers [266]. In Phase I and II clinical trials, dabrafenib demonstrated a 53–78% partial or complete response rate in melanoma patients bearing mutant V600E BRAF. In 2013, success of the Phase III clinical trial ‘BREAK-3’ led to the FDA approval of dabrafenib for the treatment of patients with mutant V600 BRAF metastatic melanomas (Table 6). However, dabrafenib has also had a number of serious side-effects reported, some of which can be life threatening, including, but not limited to, primary cutaneous malignancies, tumor promotion in BRAF wild-type melanomas, and serious febrile drug reactions. Following the success of the Phase II clinical trials, dabrafenib has been approved for the treatment of V600E mutant-BRAF NSCLC (NCT01336634) and BRAF+ anaplastic thyroid cancers (NCT01723202) [267].

Regorafenib (BAY 73–4506/ Stivarga®, Bayer) is another FDA approved type I BRAF inhibitor. However, it has not been specifically approved for use against BRAF-mutant cancers. Regorafenib is a multi-kinase inhibitor, which has been shown to inhibit both wild-type and mutant V600E BRAF in vitro [262, 268]. In early Phase I and II clinical trials, patients with advanced HCC or CRC showed 60–70% of patients maintained stable disease [269]. In 2012, following the success of Phase III clinical trials (CORRECT), regorafenib was FDA approved for the treatment of patients with metastatic CRC. Study results from this trial showed that patients treated with regorafenib plus best supportive care lived a median of 6.4 months compared to a median of 5 months in patients treated with placebo plus BSC (Table 6). The following year, the FDA approved regorafenib for the treatment of unresectable metastatic GI stromal tumors based on the ‘GRID’ Phase III trial. In this trial, patients receiving regorafenib had a significantly longer median PFS longer than patients given the placebo (4.8 vs. 0.9 months; *p < 0.000001*, Table 6). In 2017, following the results of the ‘RESORCE’ Phase III trial, regorafenib was also approved for use in patients with HCC who have previously been treated with sorafenib (Table 6). This was the first FDA-approved treatment for liver cancer in almost a decade. The most common grade 3–4 adverse reactions reported in these trials were hand-foot skin reactions, diarrhea, hypertension and fatigue [270].

A number of other small molecule inhibitors targeting BRAF have also been evaluated in vitro and are currently in clinical development for their anti-tumor activity against V600E mutant cancers [262]. These include encorafenib (LGX818), XL281 (BMS-908662), ARQ736, PLX4720, PLX3603 (RO5212054), SB-590885, GDC-0879 and RAF265 [271].

MEK kinase, which is potently activated by BRAF in the RAS/RAF/MEK/ERK pathway (Fig. 5), has also been explored as a target for new anti-cancer agents. To date, two agents, cobimetinib (Cotellic®, Exelixis and Genentech) and trametinib (Mekinist®, Noravatis), have gained FDA approved for clinical use. Cobimetinib was FDA approved in 2015 for the use in combination with vemurafenib for the treatment of advanced melanomas with BRAF V600E or V600K mutations. This was following the success of the Phase III clinical trial ‘coBRIM’, which showed a significantly longer median PFS (9.9 vs. 6.2 months; *p < 0.05)* and OS (22.3 vs. 17.4 months; *p < 0.01*) with a vemurafenib and cobimetinib combination, compared with vemurafenib alone (Table 6). Similarly, trametinib prolonged the survival of melanoma patients in the Phase III clinical trials ‘COMBI-AD’, and was FDA approved in 2018 for use in combination with dabrafenib for patients with melanomas with BRAF V600E or V600K mutations (Table 6). Trametinib has also been FDA approved for NSCLC and thyroid cancer (NCT01336634, NCT02034110; Table 6) [267].

## Conclusions

Unfortunately, while some of the anti-BRAF agents have shown promising anti-tumor activity in their clinical trials, many have been reported to have concerning toxic side effects, including the development of squamous cell carcinomas and basal cell carcinomas among others. Moreover, despite great initial responses, many trials have reported unsatisfactory median PFS, which may be in part attributed to the development of resistance through reactivation of the BRAF pathway or alternative pathways that allow for cell survival [272–274].

### Targeting T-cell immune checkpoints with CTLA-4 and PD-1 inhibitors

**Fig. 6:**
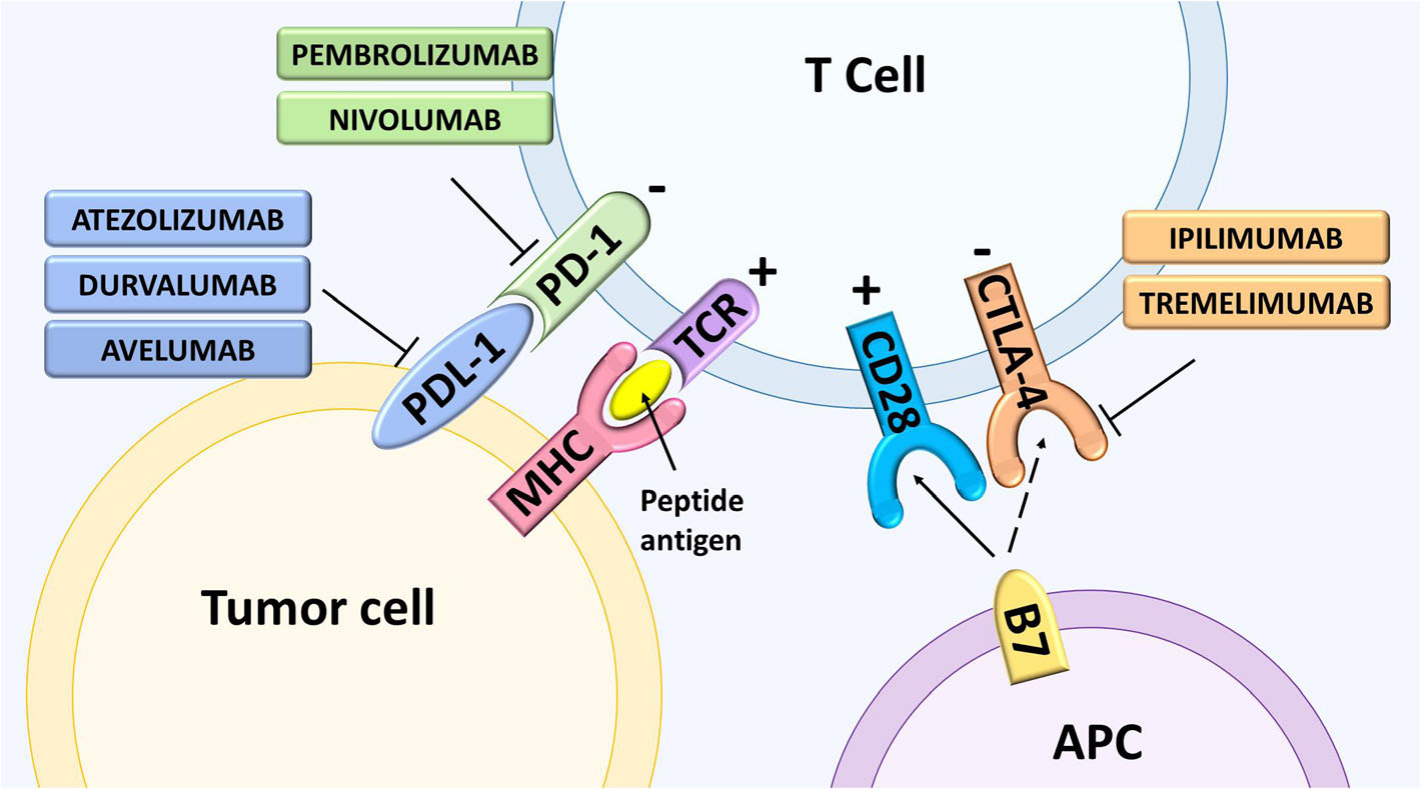
Mechanisms of action of immune checkpoint inhibitors. Two signals are required to initiate the activation of T cells. The first signal involves the binding of a MHC to a TCR on T-cells. The second signal arises with the binding of the APC B7 ligands, CD80 or CD86, to CD28 on T-cells. Cytotoxic T-lymphocyte antigen-4 (CTLA-4) competes with CD28 for the B7 ligands, which suppresses T-cell activity. Programmed cell-death protein 1 (PD-1) is also a negative regulator of T-cell activity that is able to bind to programmed cell-death 1 ligand 1 (PD-L1) on tumor cells, leading to T-cell ‘exhaustion’. Therefore, agents that act to block CTLA-4, PD-1 or PD-L1, are able to produce an anti-tumor response through immune activation. A number of these agents, including ipilimumab, tremelimumab, nivolumab, atezolizumab, durvalumab and avelumab, have been extensively studied in clinical trials for the treatment of cancer

The immune system relies on a dual signaling system for the appropriate activation of T-cells [275]. The first signal is obtained via antigen presentation to the T-cell receptor (TCR) and signal two is provided by the binding of CD28 on T-cells to one of two molecules, CD80 or CD86 (B7), on antigen-presenting cells (APCs), which promotes T-cell proliferation (Fig. 6) [275]. Immune checkpoints, and their ligands, are essential for central and peripheral tolerance. They act by counteracting the dual mechanism of signaling through the activation of co-stimulatory molecules [276]. Indeed, during immune activation, notably in chronic inflammation, T-cells upregulate a wide range of inhibitory receptors to limit their activity. These include: PD-1; CTLA-4; T-cell immunoglobulin and mucin-domain containing-3 (TIM-3); lymphocyte-activation gene 3 (LAG-3); and T-cell immunoreceptor with Ig and ITIM domains (TIGIT) [277–279]. This mechanism, referred to as ‘exhaustion’, appears to be responsible for limiting a pathological immune response during the persistent high antigenic load of infection [280]. It is now apparent that ‘exhausted’ T-cells also arise with the chronic antigen exposure occurring with cancer [281]. Gene profiling and phenotypical studies in mice and humans with cancer have shown that exhausted T-cells upregulate CTLA-4 and PD-1, which may aid in the survival of cancer cells [282, 283].

The inhibition of these surface molecules, resulting in increased activation of the immune system, has led to the development of a new range of immunotherapies. The most extensively studied of these negative regulators of immune T-cell function are CTLA-4 and PD-1 (Fig. 6) [284, 285]. Monoclonal antibodies to CTLA-4 and PD-1 are now in clinical use for melanoma and NSCLC, and they are currently undergoing further assessment for the treatment of other cancers.

### *C*ytotoxic T-lymphocyte-associated protein 4 *(CTLA-4)*

#### Background of targeted therapies to CTLA-4

CTLA-4 is a member of the CD28-B7 immunoglobulin superfamily, which acts as an immune checkpoint that downregulates immune responses [286]. It acts as an “off-switch” for T-cells and is an important part of the normal functioning of the immune system. Therefore, inhibition of CTLA-4 can shift this balance towards T-cell activation, resulting in destruction of the antigens expressed on tumor cells.

#### Clinical development of CTLA-4 inhibitors

The development of mAbs to CTLA-4 has gained widespread appeal because it is able to generate an anti-tumor T-cell response. Preclinical and clinical data has shown that the inhibition of CTLA-4 directly activates CD4+ and CD8+ effector T-cells [287, 288]. Anti–CTLA-4 mAb therapy has shown promise in several cancers, most notably in melanoma. Currently, only one agent in this class, ipilimumab (MDX-010; Yervoy®, Bristol-Myers Squibb), has received FDA approval for its anti-cancer activity. Tremelimumab (CP-675,206; AstraZeneca), another human IgG2 mAb to CTLA-4, has demonstrated some success in Phase I and II clinical trials for metastatic melanoma, but in 2008, it was terminated in Phase III trials due to treatment failure [289]. However, further analysis of survival curves within a year of treatment has shown a separation between the treatment and control groups [290]. Tremelimumab has since been assessed in clinical trials for the treatment of mesothelioma, melanoma, liver cancer, bladder cancer, NSCLC, pancreatic cancer, prostate cancer, renal cancer, urogenital cancer and head and neck cancers as well as in combination with PD-L1 inhibitors [291]. With the exception of mesothelioma, most of these trials have been met with limited success. In 2015, tremelimumab received an orphan drug designation by the FDA to treat mesothelioma, but it remains to receive FDA approval.

Ipilimumab was the first immune checkpoint inhibitor to be FDA approved for the treatment of patients with cancer. It is an anti-CTLA-4 mAb that has been demonstrated to upregulate T-cells, most notably CD4+ T-cells (Fig. 6) [292]. The Phase III clinical trial, ‘MDX010–020’, showed a median survival of 10 months in advanced melanoma patients treated with ipilimumab compared with 6 months for those treated with the experimental vaccine (gp100). In 2011, following the success of this Phase III clinical trial, ipilimumab was FDA approved for treatment of late stage melanomas (Table 7). This approval was a landmark event in the history of melanoma treatment, as it was the first ever therapy to demonstrate improved OS in a randomized Phase III trial for patients with metastatic melanoma [293]. However, due to the unusual and severe side effects arising with ipilimumab treatment, the FDA approval required a Risk Evaluation and Mitigation Strategy. Some of the severe and potentially fatal adverse effects, which occurred in 10–20% of participants, were reportedly due to the T-cell activation and proliferation effects [288, 294]. Most of these serious adverse effects were associated with gastro-intestinal tract disturbances, which occurred in up to 21% of patients, and fever, respiratory and urination problems. There have been questions raised as to the validity of the Phase III trials which led to FDA approval, as the control arm consisted of a vaccine as opposed to a placebo or standard treatment. Despite this, ipilimumab has since been approved for BRAF V600 wild-type melanomas, melanomas after surgery (NCT00636168), unresectable or metastatic melanomas (CHECKMATE-067/ NCT01696045), intermediate or poor-risk advanced RCCs (CHECKMATE-214), and metastatic CRC (CHECKMATE-142) (Table 7). In these clinical trials, there was a marked improvement in median OS and PFS compared with the control treatments (Table 7). Studies are also currently underway to assess the therapeutic effectiveness of combining ipilimumab with other immunotherapeutic agents, such as vaccines or other immunomodulatory antibodies, including nivolumab (BIOLUMA), bevacizumab (NCT00790010), and temozolomide (NCT01119508).

**Table 7:** Landmark clinical trials in the development of CTLA-4 and PD-1/PD-L1 inhibitors

CTLA-4 inhibitors										
Ipilimumab (Yervoy®)										
Drug Name	Clinical Trial ID	Trial Name	Population	Comparator	Year	Sponsor	Phase	N	Median OS (months)	Median PFS (months)
CTLA-4 inhibitors										
Ipilimumab (Yervoy®)										
Ipilimumab (3 mg/kg/3w)	NCT00094653	MDX010–020	Melanoma	Gp100 vaccine	2004–2011	Bristol-Myers Squibb	III	1783	10.0 vs 6.4	2.9 vs 2.8
Ipilimumab (10 mg/kg/3w)	NCT00636168		Melanoma	Placebo	2008–2013	Bristol-Myers Squibb	III	1211	93.5 vs 87.7	63.5 vs 56.1
Ipilimumab (3 mg/kg/3w)	NCT01696045		Melanoma	None	2012–2016	Bristol-Myers Squibb	II	14	18.2	2.6
Ipilimumab (1 mg/kg/3w) + nivolumab	NCT02231749	CHECKMATE-214	RCC	Sunitinib	2014–2017	Bristol-Myers Squibb	III	1390	NA vs 26.0	11.6 vs 8.4
Ipilimumab (1 mg/kg/3w)	NCT02060188	CHECKMATE-142	CRC	Chemotherapy	2014–2018	Bristol-Myers Squibb	II	340	NR	NR
Ipilimumab (1 mg/kg/ 6w) + nivolumab	NCT03083691	BIOLUMA	NSCLC, SCLC	Nivolumab	2017–2019	Bristol-Myers Squibb	II	106	NR	NR
Ipilimumab (10 mg/kg/3mo) + bevacizumab	NCT00790010		Melanoma	None	2009–2018	Bristol-Myers Squibb	I	46	NR	NR
Ipilimumab (10 mg/kg/3mo)	NCT01119508		Melanoma	None	2010–2016	Bristol-Myers Squibb	II	64	NR	NR
PD-1/PD-1 L inhibitors										
Pembrolizumab (Keytruda®)										
Pembrolizumab (2-10 mg/kg/3w)	NCT01295827	KEYNOTE-001	Melanoma, NSCLC	None	2011–2018	Merck Sharp & Dohme Corp.	I	1260	12.0	3.7
Pembrolizumab (2 mg/kg/3w)	NCT01704287	KEYNOTE-002	Melanoma	Chemotherapy	2012–2015	Merck Sharp & Dohme Corp.	II	540	13.4 vs 11.0	2.9 vs 2.8
Pembrolizumab (10 mg/kg/2w)	NCT01866319	KEYNOTE-006	Melanoma	Ipilimumab	2013–2015	Merck Sharp & Dohme Corp.	III	834	74.1 vs 58.2%	5.5 vs 2.8
Pembrolizumab (10 mg/kg/2w)	NCT01848834	KEYNOTE-012	Head and Neck SCC	None	2013–2016	Merck Sharp & Dohme Corp.	I	297	59%	23%
Pembrolizumab (200 mg/3w)	NCT02142738	KEYNOTE-024	NSCLC	BSC	2014–2016	Merck Sharp & Dohme Corp.	III	305	80.2 vs 72.4%	62.1 vs 50.3%
Pembrolizumab (200 mg/3w)	NCT02453594	KEYNOTE-087	Hodgkin Lymphoma	None	2015–2021	Merck Sharp & Dohme Corp.	II	211	97.5%	63.4%
Pembrolizumab (200 mg/3w) + chemotherapy	NCT02039674	KEYNOTE-021	NSCLC	Chemotherapy	2014–2016	Merck Sharp & Dohme Corp.	I/II	267	NR	13.0 vs 8.9
Pembrolizumab (200 mg/3w)	NCT02335424	KEYNOTE-052	Urothelial cancer	None	2015–2018	Merck Sharp & Dohme Corp.	II	374	67%	30%
Pembrolizumab (10 mg/kg/2w)	NCT01876511	KEYNOTE-016	CRC (MSI)	None	2013–2021	Merck Sharp & Dohme Corp.	II	171	76%	64%
Pembrolizumab (200 mg/3w)	NCT02460198	KEYNOTE-164	CRC	None	2015–2019	Merck Sharp & Dohme Corp.	II	124	NR	NR
Pembrolizumab (10 mg/kg/2w)	NCT02054806	KEYNOTE-028	Solid tumors	None	2014–2019	Merck Sharp & Dohme Corp.	I	477	62.6%	20.8%
Pembrolizumab (200 mg/3w)	NCT02628067	KEYNOTE-158	Solid tumors	None	2015–2023	Merck Sharp & Dohme Corp.	II	1350	NR	NR
Pembrolizumab (200 mg/3w)	NCT02335411	KEYNOTE-059	Gastric and gastroesophageal junction adenocarcinomas	None	2015–2019	Merck Sharp & Dohme Corp.	II	316	5.6	2.0
Pembrolizumab (200 mg/3w)	NCT02576990	KEYNOTE-170	Large B-cell lymphoma	None	2015–2019	Merck Sharp & Dohme Corp.	II	80	NR	NR
Pembrolizumab (200 mg/3w)	NCT02578680	KEYNOTE-189	NSCLC	Placebo	2016–2017	Merck Sharp & Dohme Corp.	III	646	69.2 vs 49.4%	8.8 vs 4.9
Nivolumab (Opdivo®)										
Nivolumab (3 mg/kg/2w)	NCT01721746	CHECKMATE-037	Melanoma	Chemotherapy	2012–2016	Bristol-Myers Squibb	III	631	15.7 vs 14.4	3.1 vs 3.7
Nivolumab (3 mg/kg/2w)	NCT01642004	CHECKMATE-017	NSCLC	Docetaxel	2012–2014	Bristol-Myers Squibb	III	352	9.2 vs 6.0	20.8 vs 6.4
Nivolumab (3 mg/kg/2w)	NCT01673867	CHECKMATE-057	NSCLC	Docetaxel	2012–2015	Bristol-Myers Squibb	III	792	12.2 vs 9.4	2.3 vs 4.2
Nivolumab (3 mg/kg/2w)	NCT01668784	CHECKMATE-025	RCC	Everolimus	2012–2015	Bristol-Myers Squibb	III	1068	25.0 vs 19.6	4.6 vs 4.4
Nivolumab (3 mg/kg/2w)	NCT02181738	CHECKMATE-205	Hodgkin Lymphoma	None	2014–2017	Bristol-Myers Squibb	II	338	98·7%	10.0
Nivolumab (3 mg/kg/2w)	NCT01592370	CHECKMATE-039	Hodgkin’s Lymphoma,	None	2012–2020	Bristol-Myers Squibb	I/II	375	NR	NR
Nivolumab (3 mg/kg/2w)	NCT02105636	CHECKMATE-141	Head and Neck SCC	Chemotherapy	2014–2015	Bristol-Myers Squibb	III	506	36.0 vs 16.6	NR
Nivolumab (3 mg/kg/2w)	NCT02387996	CHECKMATE-275	Advanced cancer	None	2015–2016	Bristol-Myers Squibb	II	386	8.7	2.0
Nivolumab (3 mg/kg/2w)	NCT02060188	CHECKMATE-142	CRC	None	2014–2018	Bristol-Myers Squibb	II	340	73%	14.3
Nivolumab (3 mg/kg/2w)	NCT01928394	CHECKMATE-032	Advanced solid tumors	None	2013–2018	Bristol-Myers Squibb	I/II	1150	9.7	16.2
Nivolumab (3 mg/kg/2w)	NCT01658878	CHECKMATE-040	HCC	None	2012–2019	Bristol-Myers Squibb	I/II	620	10.7	4.0
Nivolumab (1 mg/kg/3w) + ipilimumab (3 mg/kg/3w)	NCT01844505	CHECKMATE-067	Melanoma	Ipilimumab + placebo	2013–2016	Bristol-Myers Squibb	III	1296	63.8 vs 53.6%	6.9 vs 2.9
Atezolizumab (Tecentriq®)										
Atezolizumab (1200 mg/3w)	NCT02108652	IMVigor 210	Urothelial cancer	None	2014–2015	Hoffmann-La Roche	II	310	7.9	2.1
Atezolizumab (1200 mg/3w)	NCT01903993	POPLAR	NSCLC	Docetaxel	2013–2015	Hoffmann-La Roche	II	287	12.6 vs 9.7	2.7 vs 3.4
Atezolizumab (1200 mg/3w)	NCT02008227	OAK	NSCLC	Docetaxel	2014–2016	Hoffmann-La Roche	III	1225	13.8 vs 9.6	2.8 vs 4.0
Durvalumab (Imfinzi®)										
Durvalumab (10 mg/kg/2w)	NCT01693562	Study 1108	Advanced solid tumors	None	2012–2019	MedImmune LLC	I/II	1022	1.5	18.2
Durvalumab (10 mg/kg/2w)	NCT02516241	DANUBE	Urothelial cancer	None	2015–2019	AstraZeneca	III	1200	NR	NR
Durvalumab (10 mg/kg/2w)	NCT02125461	PACIFIC	NSCLC	Placebo	2014–2017	AstraZeneca	III	713	NR	16.8 vs 5.6
Avelumab (Bavencio®)										
Avelumab (10 mg/kg/2w)	NCT02155647	JAVELIN Merkel 200	Merkel Cell Carcinoma	None	2014–2019	EMD Serono	II	204	11.3	2.0
Avelumab (10 mg/kg/2w)	NCT01772004	JAVELIN Solid Tumor	Advanced solid tumors	None	2013–2018	EMD Serono	I	1758	13.7	2.7

### Programmed cell death protein 1 (PD-1) / Programmed death-ligand 1 (PD-L1)

#### Background of targeted therapies to PD-1/PD-L1

Since its initial discovery in the 1990s, the PD-1 receptor, which is found on T-cells, has been reported to negatively regulate T-cell-mediated immune responses by engaging its ligand, PD-L1, on cancer cells (Fig. 6) [295, 296]. This acts by inhibiting T-cell activation, differentiation and proliferation, leading to a state of immune tolerance [297]. This signaling pathway serves as a mechanism for tumors to evade an antigen-specific T-cell immunologic response [298, 299].

Consequently, the hypothesis was developed that PD-1/PD-L1 blockade may be an effective cancer immunotherapy. The first FDA approved anti-PD1 antibodies were nivolumab (Opdivo®, Bristol-Myers Squibb) and pembrolizumab (Keytruda®, Merck & Co.; Fig. 6). Since the approval of pembrolizumab for the treatment of advanced melanoma in 2014, the clinical development of PD-1 and PD-L1 inhibitors as anticancer agents has broadened. Presently, the FDA has approved several other PD-1/PD-L1 inhibitors, including atezolizumab (Tecentriq®, Roche), durvalumab (Imfinzi®, AstraZeneca), and avelumab (Bavencio®, Merck, Pfizer, Eli Lilly and Company) for the treatment of at least ten cancer types, including melanoma, NSCLC, head and neck squamous cell carcinoma, Hodgkin’s lymphoma, urothelial carcinoma, gastric or gastroesophageal junction cancer, cervical cancer, large B-cell lymphoma, Merkel cell carcinoma, and CRC.

#### Clinical development of PD-1/PD-1 L inhibitors

Pembrolizumab is a humanized monoclonal IgG4 antibody that is a PD-1 inhibitor [300]. In 2014, following the results of the Phase I ‘KEYNOTE-001’ and Phase 2 ‘KEYNOTE-002’ trials, pembrolizumab received FDA approval for the treatment of advanced or unresectable melanomas that are no longer responsive to other drugs (Table 7) [301, 302]. In the KEYNOTE-002 trial, median PFS (2.9 vs. 2.8 months; *p < 0.0001)* and OS (13.4 vs. 11.0 months) were greater for pembrolizumab treated patients, compared to chemotherapy. In half of the participants, who received 2 mg/kg, approximately/kg, approximately 24% had their tumors shrink [303]. This effect lasted 1.4–8.5 months and continued beyond this period in most patients [303]. Pembrolizumab was generally well tolerated in this population of patients. While drug-related adverse events occurred in 82% of patients, the most common being fatigue, pruritis and rash, only 5% had serious adverse events [302]. Adverse events that led to discontinuation, included pneumonitis, renal failure and pain.

In 2015, pembrolizumab received an expanded first-line indication to include previously untreated advanced melanomas regardless of their BRAF mutation status, following the results of the ‘KEYNOTE-006’ clinical trial (Table 7). One-year OS and ORR rates were significantly improved in patients receiving pembrolizumab compared to ipilimumab. The most common adverse effects were colitis and hepatitis. Pembrolizumab has also been FDA approved for ipilimumab-refractory melanomas based on the ‘KEYNOTE-002’ clinical trials (Table 7). Since 2015, the FDA has approved pembrolizumab for the treatment of advanced/metastatic NSCLC (KEYNOTE-001), recurrent/metastatic head and neck squamous cell carcinoma (KEYNOTE-012), high PD-1 expressing metastatic NSCLC (KEYNOTE-024), classical Hodgkin lymphoma (KEYNOTE-087), first-line metastatic non-squamous NSCLC irrespective of PD-L1 expression (KEYNOTE-021), locally advanced/metastatic urothelial carcinoma (KEYNOTE-052), unresectable or metastatic solid tumors with unresectable or metastatic microsatellite instability–high (MSI-H) or mismatch repair deficient (dMMR) solid tumors (KEYNOTE-016, − 164, − 012, − 028, and − 158), advanced/metastatic gastric or gastroesophageal junction cancers expressing PD-L1 (KEYNOTE-059), metastatic cervical cancers expressing PD-L1 (KEYNOTE-158), refractory or relapsed primary mediastinal large B-Cell lymphomas (PMBCL; KEYNOTE-170), and metastatic non-squamous NSCLCs with no EGFR or ALK mutations (KEYNOTE-189; Table 7) [304–309].

Nivolumab is also a fully human monoclonal IgG4 antibody to PD-1 [310, 311]. It was first granted accelerated approval as a new treatment for patients with unresectable or metastatic melanoma which were no longer responsive to other drugs. This was based on the ‘CHECKMATE-037’ trial of 272 patients with advanced melanoma (Table 7) [312]. Nivolumab led to a greater proportion of patients achieving an objective response and fewer toxic effects than with alternative available chemotherapy regimens. Results showed that 32% of participants receiving treatment had their tumors shrink, with the reduced tumor size persisting longer than 6 months in 1/3 of those patients [313]. The most common side effects were rash, itching, cough, upper respiratory tract infections, and edema [313, 314]. The most serious side effects were severe immune-mediated side effects involving the lung, colon, liver, kidneys and endocrine system [314, 315].

In March 2015, nivolumab was FDA approved for the treatment of metastatic squamous NSCLC with progression after platinum-based chemotherapy, following results of the ‘CHECKMATE-017’ trial (Table 7). In this randomized trial of 272 participants, patients who received nivolumab lived 3.2 months longer than those who received docetaxel. Later in 2015, nivolumab was also approved for the treatment of advanced non-squamous NSCLC, as patients treated with nivolumab in the ‘CHECKMATE-057’ trials lived an average of 12.2 months compared to 9.4 months in those treated with docetaxel [316]. Since then, nivolumab has been FDA approved for the treatment of advanced SCLC (CHECKMATE-032), classical Hodgkin lymphoma (CHECKMATE-205, CHECKMATE-039), advanced squamous cell carcinoma of the head and neck (CHECKMATE-141), urothelial carcinoma (CHECKMATE-275), HCC (CHECKMATE-040), MSI-H or dMMR metastatic CRC (CHECKMATE-142), and advanced RCC (CHECKMATE-025; Table 7) [317–321]. The results of CHECKMATE-025 mark the first time an immuno-oncology agent has demonstrated a survival advantage in advanced RCC, a patient group that currently has limited treatment options.

In 2016, nivolumab in combination with ipilimumab was FDA approved for the treatment of patients with BRAF V600 wild-type and BRAF V600+ unresectable or advanced melanomas [322]. This combination received accelerated approval based on median PFS in the Phase III ‘CHECKMATE-067’ clinical trials (Table 7). The results of this trial of 945 previously untreated patients demonstrated a significant improvement in median PFS in patients with advanced melanoma treated with the combination therapy and with nivolumab alone, compared with ipilimumab alone (*p < 0.0001 and p < 0.0001,* respectively) [323]. Therefore, these preliminary trials highlight the therapeutic potential of this type of combination approach for the treatment of cancer.

Atezolizumab is a new PD-L1 inhibitor, that was FDA approved in 2016, for the treatment of urothelial carcinomas following progression after platinum therapy or surgery [324]. While patients receiving atezolizumab experienced an anti-tumor response across the study, the greatest effect occurred in participants with PD-L1 expressing cancers [325, 326]. Therefore, the FDA also approved the Ventana PD-L1 (SP142) assay (Ventana Medical Systems, USA) for the detection of PD-L1 expression to determine the patients that are most likely to benefit from atezolizumab treatment. Approval of atezolizumab for patients with advanced urothelial carcinomas was determined in the ‘IMvigor 210’ clinical trial involving 310 patients with advanced urothelial carcinomas. In patients with positive PD-L1 expression, 26% experienced a tumor response, compared with 9.5% in those that were PD-L1 negative (Table 7) [327]. The most common side effects of treatment were fatigue, decreased appetite, nausea, urinary tract infection, pyrexia and constipation [328]. More severe immune-mediated side effects were also observed. Atezolizumab has since also been FDA approved for advanced urothelial cancer in patients who are not eligible for cisplatin therapy. Following the Phase II ‘POPLAR’ and Phase III ‘OAK’ studies, atezolizumab was also FDA approved in 2016 for the treatment of metastatic NSCLC (Table 7). In the ‘OAK’ study that enrolled patients with NSCLC, regardless of their PD-L1 status, median OS was 13.8 months in atezolizumab treated patients, which was 4.2 months longer than those treated with docetaxel chemotherapy.

Durvalumab is another anti-PD-L1 human mAb that is indicated for the treatment of patients with metastatic urothelial carcinomas and patients with unresectable NSCLC that have not progressed after chemoradiation. In 2017, the FDA accelerated approval of durvalumab for the treatment of advanced bladder cancer based on data from the Phase I/II clinical trial ‘Study 1108’ (Table 7). The ORR of this study was 26.3% in patients with highly PD-L1 expressing tumors, compared with 17.0% in all evaluable patients regardless of their PD-L1 status [329]. Additionally, 14.3% of all evaluable patients achieved partial response and 2.7% achieved complete response (Table 7). Currently, durvalumab is also under investigation in the Phase III ‘DANUBE’ trial as a first-line treatment in urothelial carcinoma as monotherapy and in combination with the CTLA-4 inhibitor, tremelimumab (Table 7) [330]. Early in 2018, durvalumab was also approved for the treatment of stage III unresectable NSCLC following the success of the ‘PACIFIC’ Phase III trials, which showed a median PFS for patients taking durvalumab of 16.8 months compared to 5.6 months for patients receiving the placebo (Table 7) [331].

Avelumab is also a PD-L1 blocking human monoclonal IgG1 antibody that is indicated for the treatment of patients with metastatic Merkel cell carcinoma (MCC) and urothelial carcinoma [332]. In 2017, the FDA approved durvalumab for the first-line treatment of metastatic MCC, a rare and aggressive skin cancer. Approval was based on data from the ‘JAVELIN Merkel 200’ trial, where 33% of patients had a complete or partial shrinkage of their tumors, which lasted for more than 6 months in 86% of responding patients and more than 12 months in 45% of responding patients (Table 7) [333]. In May of the same year, avelumab was also FDA approved for the treatment of patients with advanced urothelial carcinomas following platinum therapy. This approval was based on data from a 1758 patient Phase I trial ‘JAVELIN solid tumor’, which demonstrated a clinically meaningful ORR (33%, with 11% complete and 22% partial; Table 7) [334]. Serious adverse reactions were reported in 8% of patients. The most frequent of these were urosepsis, abdominal pain, musculoskeletal pain, creatinine increased/renal failure, dehydration, hematuria/urinary tract hemorrhage, intestinal obstruction, and pyrexia. Adverse reactions causing death occurred in one patient [335].

## Conclusion

The immune-checkpoint pathways, which have been shown to downregulate T-cell activation to maintain peripheral tolerance, are exploited by tumors to induce an immunosuppressive state that allows the tumors to evade the immune system. Consequently, immune-checkpoint inhibitors, CTLA-4, PD-1 and PD-L1, have emerged as both important cancer biomarkers and targets for immunotherapy.

As we have discussed above, the data that has become available over recent years from clinical trials, provides the proof-of-concept that blocking negative immune regulatory pathways can lead to marked tumor responses. Some of the more encouraging data is the long-lived tumor regression arising from CTLA-4-inhibiting mAbs in patients with advanced melanoma. Unfortunately, at this stage, there remain significant immune-mediated toxicities arising from these agents. However, it appears that most of these are manageable with corticosteroid treatment [336, 337]. Due to their mechanism of action, these agents may facilitate activation of potentially autoreactive T-cells, leading to inflammatory adverse-effects across a range of tissues, contributing to the immune-mediated side effects discussed above. Consequently, patients with a history of autoimmune disease or systemic immune suppression were excluded from clinical trials with PD-1 pathway inhibitors [338, 339]. An improved understanding on the mechanisms causing toxicity may allow for improved adjuvant treatments to reduce these adverse effects.

Interestingly, the improved efficacy of the simultaneous blockade of both CTLA-4 and PD-1 pathways over CTLA-4 or PD-1 inhibition alone, provides evidence of the separate roles of these checkpoints in regulating antitumor immune responses. Indeed, it has been reported that, while both CTLA-4 and PD-1 have similar negative effects on T-cell activity, the timing, location and signaling pathways differ [21]. In fact, the difference in distribution of the CTLA-4 and PD-1 ligands, which are found primarily in lymphoid tissue and in peripheral tissues, respectively, is central to the hypothesis that CTLA-4 acts early in tolerance induction and PD-1 acts late to maintain long-term tolerance. This suggests that combinatorial approaches may have superior survival outcomes compared to single-agent immunotherapy regimens. The therapeutic potential of combinatorial approaches is highlighted by the recent FDA approval of nivolumab plus ipilimumab for patients with advanced melanoma. Therefore, further trials are warranted to validate similar combination strategies for the treatment of other cancer types. Indeed, there are current dual-immune checkpoint inhibition with anti-PD-1/PD-L1 plus anti-CTLA-4 mAbs being evaluated for a wide range of tumors [340]. Furthermore, several ongoing clinical trials are investigating combination checkpoint inhibition in association with traditional treatment modalities, such as chemotherapy, surgery, and radiation, and with newer therapies, such as the modified herpes simplex virus, talimogene laherparepvec [341].

## Summary

The development of small-molecule inhibitors and monoclonal antibodies for the targeted treatment of cancer has been rapidly expanding in recent years, greatly facilitated by the passing of the FDA Safety Innovations Act by the United States Congress in 2012. This act allows for the use of surrogate clinical endpoints (such as a lab endpoints or radiographic images), which predict clinical benefit, rather than measures of clinical benefit (such as OS or PFS). This significantly accelerates the progression of drugs for cancers with unmet medical need from the bench to the bedside and has been utilised by many of the drugs discussed herein.

The specificity, lower toxicity, and immune system activating abilities of these agents have been very promising for the treatment of cancer. We have seen several of these drugs become standard of care for cancer treatment, including cetuximab, durvalumab and ceritinib. One of the more exciting recent developments has been the clinical approval of immune checkpoint inhibitors. These include the CTLA-4, PD-1, and PD-L1 inhibitors, which restore anti-tumor immune responses, leading to a longer survival in a significant proportion of treated patients. These also remain in active clinical development for multiple indications for oncology and have the potential to revolutionize future treatment options for many patients with advanced cancer.

Interestingly, this area of drug development highlights the importance of more personalized treatment. Identifying patients who are most likely to benefit from these selective mAbs is crucial to improving therapeutic outcomes. As we have seen, these agents principally are involved in targeting specific dysregulated protein expression. Therefore, there is evidence that monitoring variations in gene copy numbers, gene mutations, and protein expression could present as useful biomarkers for the selection of patients who are most likely respond to treatment. Indeed, this biomarker guided treatment selection is in routine practice in breast cancer, where a positive HER2 status is mandatory in selecting patients for treatment with anti-HER2 therapy.

One of the limitations of these targeted therapies, as with standard chemotherapies, has been the development of drug resistance. However, as we have seen with several of the drugs mentioned in this paper, the use of these therapies in combination with other targeted agents, immunotherapies or standard chemotherapies, can overcome this problem. It is possible that the dramatic tumor regressions induced by targeted therapies can be converted into durable responses by the concomitant use of immunotherapies, which induce host-tumor responses. Furthermore, despite the important advances made in targeting molecular drivers of cancer, some targets have eluded drug therapies thus far. A notable example is KRAS, which is highly expressed in many types of cancer [342]. Considering how difficult it has been to target, the National Institute of Health started the RAS initiative, aimed at specifically targeting KRAS mutations. While no specific KRAS targeted therapy is yet being trialed, there are currently 80 active trials on the ClinicalTrials.gov website utilizing many of the targeted or immune based therapies discussed herein, offering hope that a successful drug regimen may be discovered soon.

Over the next few decades, as we advance our understanding of immune system regulation, we can expect to see further optimization of antibody structures and the identification of new targets, leading to more effective treatment options. We can also expect that trials will demonstrate the efficacy of combining immunotherapies with targeted treatments, and this will offer further benefit to patients.

## Abbreviations

ADCC: Antibody-dependent cellular cytotoxicity
AKT: Protein kinase B
APC: Antigen presenting cells
ATP: Adenosine 5′ triphosphate
BSC: Best supportive care
CRC: Colorectal cancer
CTLA-4: Cytotoxic T-lymphocyte-associated protein 4
DFS: Disease free survival
EGFR: Epidermal growth factor receptor
FDA: Food and drug administration
FOLFIRI: Folinic acid, fluorouracil and irinotecan
HCC: Hepatocellular carcinoma
HER2: Human epidermal growth factor receptor-2
HER3: Human epidermal growth factor receptor-3
HER4: Human epidermal growth factor receptor-4
HIF-1α: Hypoxia inducible factor-1alpha
HSP: Heat-shock protein
KRAS: Kirsten rat sarcoma viral oncogene homolog
mAbs: Monoclonal antibodies
MPP: Metalloproteinases
MSI: Microsatellite unstable
NCI: National Cancer Institute
nRTK: Non-receptor tyrosine kinases
NR: Not reported
NSCLC: Non-small cell lung cancer
Obs: Observation
ORR: Objective response rate
OS: Overall survival
PD-1: Programmed cell death protein-1
PFS: Progression-free survival
RCC: Renal cell carcinoma
RTK: Receptor tyrosine kinases
TCR: T-cell receptor
TGFα: Transforming growth factor alpha
TKI: Tyrosine kinase inhibitors
TTP: Time to disease progression
VEGF: Vascular endothelial growth factor

## References

[1] Bray F, Ferlay J, Soerjomataram I, Siegel RL, Torre LA, Jemal A (2018). Global cancer statistics 2018: GLOBOCAN estimates of incidence and mortality worldwide for 36 cancers in 185 countries. CA Cancer J Clin..

[2] Baskar R, Lee KA, Yeo R, Yeoh K-W (2012). Cancer and radiation therapy: current advances and future directions. Int J Med Sci..

[3] Palumbo MO, Kavan P, Miller WH, Panasci L, Assouline S, Johnson N (2013). Systemic cancer therapy: achievements and challenges that lie ahead. Front Pharmacol..

[4] Schuck A, Konemann S, Heinen K, Rube CE, Hesselmann S, Reinartz G (2002). Microscopic residual disease is a risk factor in the primary treatment of breast cancer. Strahlentherapie und Onkologie : Organ der Deutschen Rontgengesellschaft..

[5] Jacobson LK, Johnson MB, Dedhia RD, Niknam-Bienia S, Wong AK (2017). Impaired wound healing after radiation therapy: a systematic review of pathogenesis and treatment. JPRAS Open..

[6] Formenti SC, Demaria S (2009). Systemic effects of local radiotherapy. Lancet Oncol..

[7] Malhotra V, Perry MC (2003). Classical chemotherapy: mechanisms, toxicities and the therapeutic window. Cancer Biol Ther..

[8] Hoelder S, Clarke PA, Workman P (2012). Discovery of small molecule cancer drugs: successes, challenges and opportunities. Mol Oncol..

[9] Hojjat-Farsangi M (2014). Small-molecule inhibitors of the receptor tyrosine kinases: promising tools for targeted Cancer therapies. Int J Mol Sci..

[10] Eck MJ, Manley PW (2009). The interplay of structural information and functional studies in kinase drug design: insights from BCR-Abl. Curr Opin Cell Biol..

[11] Iqbal N, Iqbal N (2014). Imatinib: a breakthrough of targeted therapy in Cancer. Chemother Res Pract..

[12] Lavanya V, Mohamed Adil AA, Ahmed N, Rishi AK, Jamal S (2014). Small molecule inhibitors as emerging cancer therapeutics. Integr Cancer Sci Therap..

[13] Lambert JM (2005). Drug-conjugated monoclonal antibodies for the treatment of cancer. Curr Opin Pharmacol..

[14] Argyriou AA, Kalofonos HP (2009). Recent advances relating to the clinical application of naked monoclonal antibodies in solid tumors. Mol Med..

[15] Jacobs SA (2007). (90)Yttrium ibritumomab tiuxetan in the treatment of non-Hodgkin’s lymphoma: current status and future prospects. Biologics..

[16] van de Donk NWCJ, Dhimolea E (2012). Brentuximab vedotin. mAbs.

[17] Baron JM, Boster BL, Barnett CM (2015). Ado-trastuzumab emtansine (T-DM1): a novel antibody-drug conjugate for the treatment of HER2-positive metastatic breast cancer. J Oncol Pharm Pract..

[18] Hoffman LM, Gore L (2014). Blinatumomab, a bi-specific anti-CD19/CD3 BiTE(®) antibody for the treatment of acute lymphoblastic leukemia: perspectives and current pediatric applications. Front Oncol..

[19] Chen X, Cai H (2016). Monoclonal antibodies for Cancer therapy approved by FDA. MOJ Immunol..

[20] Hanahan D, Weinberg RA (2011). Hallmarks of cancer: the next generation. Cell..

[21] Buchbinder EI, Desai A (2016). CTLA-4 and PD-1 pathways: similarities, differences, and implications of their inhibition. Am J Clin Oncol..

[22] Whiteside TL (2015). The role of regulatory T cells in cancer immunology. Immunotargets Ther..

[23] Schlessinger J (2000). Cell signaling by receptor tyrosine kinases. Cell..

[24] Carpenter G, King L, Cohen S (1978). Epidermal growth factor stimulates phosphorylation in membrane preparations in vitro. Nature..

[25] Lax I, Bellot F, Howk R, Ullrich A, Givol D, Schlessinger J (1989). Functional analysis of the ligand binding site of EGF-receptor utilizing chimeric chicken/human receptor molecules. EMBO J..

[26] Lemmon MA, Bu Z, Ladbury JE, Zhou M, Pinchasi D, Lax I (1997). Two EGF molecules contribute additively to stabilization of the EGFR dimer. EMBO J..

[27] Garrett TP, McKern NM, Lou M, Elleman TC, Adams TE, Lovrecz GO (2002). Crystal structure of a truncated epidermal growth factor receptor extracellular domain bound to transforming growth factor alpha. Cell..

[28] Markman B, Javier Ramos F, Capdevila J, Tabernero J (2010). EGFR and KRAS in colorectal cancer. Adv Clin Chem..

[29] Lee JC, Vivanco I, Beroukhim R, Huang JH, Feng WL, DeBiasi RM (2006). Epidermal growth factor receptor activation in glioblastoma through novel missense mutations in the extracellular domain. PLoS Med..

[30] Blume-Jensen P, Hunter T (2001). Oncogenic kinase signalling. Nature..

[31] Lui VW, Grandis JR (2002). EGFR-mediated cell cycle regulation. Anticancer Res..

[32] Huang PH, Xu AM, White FM (2009). Oncogenic EGFR signaling networks in glioma. Sci Signal.

[33] Midha A, Dearden S, McCormack R (2015). EGFR mutation incidence in non-small-cell lung cancer of adenocarcinoma histology: a systematic review and global map by ethnicity (mutMapII). Am J Cancer Res..

[34] Shigematsu H, Lin L, Takahashi T, Nomura M, Suzuki M, Wistuba II (2005). Clinical and biological features associated with epidermal growth factor receptor gene mutations in lung cancers. J Natl Cancer Inst..

[35] Sonobe M, Manabe T, Wada H, Tanaka F (2005). Mutations in the epidermal growth factor receptor gene are linked to smoking-independent, lung adenocarcinoma. Br J Cancer..

[36] Zhang X, Gureasko J, Shen K, Cole PA, Kuriyan J (2006). An allosteric mechanism for activation of the kinase domain of epidermal growth factor receptor. Cell..

[37] Fujino S, Enokibori T, Tezuka N, Asada Y, Inoue S, Kato H (1996). A comparison of epidermal growth factor receptor levels and other prognostic parameters in non-small cell lung cancer. Eur J Cancer.

[38] Cappuzzo F, Finocchiaro G, Rossi E, Janne PA, Carnaghi C, Calandri C (2008). EGFR FISH assay predicts for response to cetuximab in chemotherapy refractory colorectal cancer patients. Ann Oncol..

[39] Seshacharyulu P, Ponnusamy MP, Haridas D, Jain M, Ganti A, Batra SK (2012). Targeting the EGFR signaling pathway in cancer therapy. Expert Opin Ther Targets..

[40] Ciardiello F, Tortora G (2008). EGFR antagonists in cancer treatment. N Engl J Med..

[41] Normanno N, Bianco C, De Luca A, Maiello MR, Salomon DS (2003). Target-based agents against ErbB receptors and their ligands: a novel approach to cancer treatment. Endocr Relat Cancer..

[42] Scheffler M, Di Gion P, Doroshyenko O, Wolf J, Fuhr U (2011). Clinical pharmacokinetics of tyrosine kinase inhibitors: focus on 4-anilinoquinazolines. Clin Pharmacokinet..

[43] Pao W, Miller V, Zakowski M, Doherty J, Politi K, Sarkaria I (2004). EGF receptor gene mutations are common in lung cancers from “never smokers” and are associated with sensitivity of tumors to gefitinib and erlotinib. Proc Natl Acad Sci U S A..

[44] Lynch TJ, Bell DW, Sordella R, Gurubhagavatula S, Okimoto RA, Brannigan BW (2004). Activating mutations in the epidermal growth factor receptor underlying responsiveness of non-small-cell lung cancer to gefitinib. N Engl J Med..

[45] Paez JG, Janne PA, Lee JC, Tracy S, Greulich H, Gabriel S (2004). EGFR mutations in lung cancer: correlation with clinical response to gefitinib therapy. Science..

[46] Cohen MH, Williams GA, Sridhara R, Chen G, Pazdur R (2003). FDA drug approval summary: gefitinib (ZD1839) (Iressa) tablets. Oncologist..

[47] Kazandjian D, Blumenthal GM, Yuan W, He K, Keegan P, Pazdur R (2016). FDA approval of Gefitinib for the treatment of patients with metastatic EGFR mutation-positive non-small cell lung Cancer. Clin Cancer Res..

[48] Mok TS, Wu YL, Thongprasert S, Yang CH, Chu DT, Saijo N (2009). Gefitinib or carboplatin-paclitaxel in pulmonary adenocarcinoma. N Engl J Med..

[49] Zhao H, Fan Y, Ma S, Song X, Han B, Cheng Y (2015). Final overall survival results from a phase III, randomized, placebo-controlled, parallel-group study of gefitinib versus placebo as maintenance therapy in patients with locally advanced or metastatic non-small-cell lung cancer (INFORM; C-TONG 0804). J Thorac Oncol..

[50] Thatcher N, Chang A, Parikh P, Rodrigues Pereira J, Ciuleanu T, von Pawel J (2005). Gefitinib plus best supportive care in previously treated patients with refractory advanced non-small-cell lung cancer: results from a randomised, placebo-controlled, multicentre study (Iressa Survival Evaluation in Lung Cancer). Lancet..

[51] Hartmann JT, Haap M, Kopp HG, Lipp HP (2009). Tyrosine kinase inhibitors - a review on pharmacology, metabolism and side effects. Curr Drug Metab..

[52] Segovia-Mendoza M, González-González ME, Barrera D, Díaz L, García-Becerra R (2015). Efficacy and mechanism of action of the tyrosine kinase inhibitors gefitinib, lapatinib and neratinib in the treatment of HER2-positive breast cancer: preclinical and clinical evidence. Am J Cancer Res..

[53] Cappuzzo F, Finocchiaro G, Metro G, Bartolini S, Magrini E, Cancellieri A (2006). Clinical experience with gefitinib: an update. Crit Rev Oncol Hematol..

[54] Herbst RS, LoRusso PM, Purdom M, Ward D (2003). Dermatologic side effects associated with gefitinib therapy: clinical experience and management. Clin Lung Cancer..

[55] Johnson DH (2003). Gefitinib (Iressa) trials in non-small cell lung cancer. Lung Cancer..

[56] Stamos J, Sliwkowski MX, Eigenbrot C (2002). Structure of the epidermal growth factor receptor kinase domain alone and in complex with a 4-anilinoquinazoline inhibitor. J Biol Chem..

[57] Cohen MH, Johnson JR, Chen YF, Sridhara R, Pazdur R (2005). FDA drug approval summary: erlotinib (Tarceva) tablets. Oncologist..

[58] Shepherd FA, Rodrigues Pereira J, Ciuleanu T, Tan EH, Hirsh V, Thongprasert S (2005). Erlotinib in previously treated non-small-cell lung cancer. N Engl J Med..

[59] Cappuzzo F, Ciuleanu T, Stelmakh L, Cicenas S, Szczesna A, Juhasz E (2009). SATURN: A double-blind, randomized, phase III study of maintenance erlotinib versus placebo following nonprogression with first-line platinum-based chemotherapy in patients with advanced NSCLC. J Clin Oncol..

[60] Kiyohara Y, Yamazaki N, Kishi A (2013). Erlotinib-related skin toxicities: treatment strategies in patients with metastatic non-small cell lung cancer. J Am Acad Dermatol..

[61] Johnston SR, Leary A (2006). Lapatinib: a novel EGFR/HER2 tyrosine kinase inhibitor for cancer. Drugs Today (Barc)..

[62] Cameron D, Casey M, Oliva C, Newstat B, Imwalle B, Geyer CE (2010). Lapatinib plus Capecitabine in women with HER-2–positive advanced breast Cancer: final survival analysis of a Phase III randomized trial. Oncologist..

[63] Janne PA, Wang X, Socinski MA, Crawford J, Stinchcombe TE, Gu L (2012). Randomized phase II trial of erlotinib alone or with carboplatin and paclitaxel in patients who were never or light former smokers with advanced lung adenocarcinoma: CALGB 30406 trial. J Clin Oncol..

[64] Maemondo M, Inoue A, Kobayashi K, Sugawara S, Oizumi S, Isobe H (2010). Gefitinib or chemotherapy for non-small-cell lung cancer with mutated EGFR. N Engl J Med..

[65] Pao W, Miller VA, Politi KA, Riely GJ, Somwar R, Zakowski MF (2005). Acquired resistance of lung adenocarcinomas to gefitinib or erlotinib is associated with a second mutation in the EGFR kinase domain. PLoS Med..

[66] Toyooka S, Kiura K, Mitsudomi T (2005). EGFR mutation and response of lung cancer to gefitinib. N Engl J Med.

[67] Li D, Ambrogio L, Shimamura T, Kubo S, Takahashi M, Chirieac LR (2008). BIBW2992, an irreversible EGFR/HER2 inhibitor highly effective in preclinical lung cancer models. Oncogene..

[68] Wissner A, Overbeek E, Reich MF, Floyd MB, Johnson BD, Mamuya N (2003). Synthesis and structure-activity relationships of 6,7-disubstituted 4-anilinoquinoline-3-carbonitriles. The design of an orally active, irreversible inhibitor of the tyrosine kinase activity of the epidermal growth factor receptor (EGFR) and the human epidermal growth factor receptor-2 (HER-2). J Med Chem..

[69] Smaill JB, Showalter HD, Zhou H, Bridges AJ, McNamara DJ, Fry DW (2001). Tyrosine kinase inhibitors. 18. 6-substituted 4-anilinoquinazolines and 4-anilinopyrido[3,4-d]pyrimidines as soluble, irreversible inhibitors of the epidermal growth factor receptor. J Med Chem..

[70] Tsou HR, Overbeek-Klumpers EG, Hallett WA, Reich MF, Floyd MB, Johnson BD (2005). Optimization of 6,7-disubstituted-4-(arylamino)quinoline-3-carbonitriles as orally active, irreversible inhibitors of human epidermal growth factor receptor-2 kinase activity. J Med Chem..

[71] Engelman JA, Zejnullahu K, Gale CM, Lifshits E, Gonzales AJ, Shimamura T (2007). PF00299804, an irreversible pan-ERBB inhibitor, is effective in lung cancer models with EGFR and ERBB2 mutations that are resistant to gefitinib. Cancer Res..

[72] Fry DW (2003). Mechanism of action of erbB tyrosine kinase inhibitors. Exp Cell Res..

[73] Garuti L, Roberti M, Bottegoni G (2011). Irreversible protein kinase inhibitors. Curr Med Chem..

[74] Wissner A, Fraser HL, Ingalls CL, Dushin RG, Floyd MB, Cheung K (2007). Dual irreversible kinase inhibitors: quinazoline-based inhibitors incorporating two independent reactive centers with each targeting different cysteine residues in the kinase domains of EGFR and VEGFR-2. Bioorg Med Chem..

[75] Morabito A, Piccirillo MC, Falasconi F, De Feo G, Del Giudice A, Bryce J (2009). Vandetanib (ZD6474), a dual inhibitor of vascular endothelial growth factor receptor (VEGFR) and epidermal growth factor receptor (EGFR) tyrosine kinases: current status and future directions. Oncologist..

[76] Feldinger K, Kong A (2015). Profile of neratinib and its potential in the treatment of breast cancer. Breast Cancer..

[77] Gonzales AJ, Hook KE, Althaus IW, Ellis PA, Trachet E, Delaney AM (2008). Antitumor activity and pharmacokinetic properties of PF-00299804, a second-generation irreversible pan-erbB receptor tyrosine kinase inhibitor. Mol Cancer Ther..

[78] Rabindran SK, Discafani CM, Rosfjord EC, Baxter M, Floyd MB, Golas J (2004). Antitumor activity of HKI-272, an orally active, irreversible inhibitor of the HER-2 tyrosine kinase. Cancer Res..

[79] Wedge SR, Ogilvie DJ, Dukes M, Kendrew J, Chester R, Jackson JA (2002). ZD6474 inhibits vascular endothelial growth factor signaling, angiogenesis, and tumor growth following oral administration. Cancer Res..

[80] Torrance CJ, Jackson PE, Montgomery E, Kinzler KW, Vogelstein B, Wissner A (2000). Combinatorial chemoprevention of intestinal neoplasia. Nat Med..

[81] Smaill JB, Rewcastle GW, Loo JA, Greis KD, Chan OH, Reyner EL (2000). Tyrosine kinase inhibitors. 17. Irreversible inhibitors of the epidermal growth factor receptor: 4-(phenylamino)quinazoline- and 4-(phenylamino)pyrido[3,2-d]pyrimidine-6-acrylamides bearing additional solubilizing functions. J Med Chem..

[82] Solca F, Dahl G, Zoephel A, Bader G, Sanderson M, Klein C (2012). Target binding properties and cellular activity of afatinib (BIBW 2992), an irreversible ErbB family blocker. J Pharmacol Exp Ther..

[83] Smith S, Keul M, Engel J, Basu D, Eppmann S, Rauh D (2017). Characterization of covalent-reversible EGFR inhibitors. ACS Omega..

[84] Nelson V, Ziehr J, Agulnik M, Johnson M (2013). Afatinib: emerging next-generation tyrosine kinase inhibitor for NSCLC. OncoTargets Therapy..

[85] Giaccone G, Wang Y (2011). Strategies for overcoming resistance to EGFR family tyrosine kinase inhibitors. Cancer Treat Rev..

[86] Ninomiya T, Takigawa N, Ichihara E, Ochi N, Murakami T, Honda Y (2013). Afatinib prolongs survival compared with gefitinib in an epidermal growth factor receptor-driven lung cancer model. Mol Cancer Ther..

[87] Sequist LV, Yang JC, Yamamoto N, O'Byrne K, Hirsh V, Mok T (2013). Phase III study of afatinib or cisplatin plus pemetrexed in patients with metastatic lung adenocarcinoma with EGFR mutations. J Clin Oncol..

[88] Geater SL, Zhou C, Hu C-P, Feng JF, Lu S, Huang Y (2013). LUX-Lung 6: Patient-reported outcomes (PROs) from a randomized open-label, phase III study in first-line advanced NSCLC patients (pts) harboring epidermal growth factor receptor (EGFR) mutations. J Clin Oncol.

[89] Wu YL, Zhou C, Hu CP, Feng J, Lu S, Huang Y (2014). Afatinib versus cisplatin plus gemcitabine for first-line treatment of Asian patients with advanced non-small-cell lung cancer harbouring EGFR mutations (LUX-lung 6): an open-label, randomised phase 3 trial. Lancet Oncol..

[90] Yang JC, Wu YL, Schuler M, Sebastian M, Popat S, Yamamoto N (2015). Afatinib versus cisplatin-based chemotherapy for EGFR mutation-positive lung adenocarcinoma (LUX-lung 3 and LUX-lung 6): analysis of overall survival data from two randomised, phase 3 trials. Lancet Oncol..

[91] Lin NU, Winer EP, Wheatley D, Carey LA, Houston S, Mendelson D (2012). A phase II study of afatinib (BIBW 2992), an irreversible ErbB family blocker, in patients with HER2-positive metastatic breast cancer progressing after trastuzumab. Breast Cancer Res Treat..

[92] Kalous O, Conklin D, Desai AJ, O'Brien NA, Ginther C, Anderson L (2012). Dacomitinib (PF-00299804), an irreversible pan-HER inhibitor, inhibits proliferation of HER2-amplified breast cancer cell lines resistant to trastuzumab and lapatinib. Mol Cancer Ther..

[93] Wu YL, Cheng Y, Zhou X, Lee KH, Nakagawa K, Niho S (2017). Dacomitinib versus gefitinib as first-line treatment for patients with EGFR-mutation-positive non-small-cell lung cancer (ARCHER 1050): a randomised, open-label, phase 3 trial. Lancet Oncol..

[94] Thornton K, Kim G, Maher VE, Chattopadhyay S, Tang S, Moon YJ (2012). Vandetanib for the treatment of symptomatic or progressive medullary thyroid cancer in patients with unresectable locally advanced or metastatic disease: U.S. Food and Drug Administration drug approval summary. Clin Cancer Res..

[95] Kim ES, Herbst RS, Wistuba II, Lee JJ, Blumenschein GR, Tsao A (2011). The BATTLE trial: personalizing therapy for lung cancer. Cancer Discov..

[96] Natale RB, Thongprasert S, Greco FA, Thomas M, Tsai CM, Sunpaweravong P (2011). Phase III trial of vandetanib compared with erlotinib in patients with previously treated advanced non-small-cell lung cancer. J Clin Oncol..

[97] Yu HA, Riely GJ (2013). Second generation epidermal growth factor receptor tyrosine kinase inhibitors in lung cancers. J Natl Compr Canc Netw..

[98] Chan A, Delaloge S, Holmes FA, Moy B, Iwata H, Harvey VJ (2016). Neratinib after trastuzumab-based adjuvant therapy in patients with HER2-positive breast cancer (ExteNET): a multicentre, randomised, double-blind, placebo-controlled, phase 3 trial. Lancet Oncol..

[99] Gandhi L, Bahleda R, Tolaney SM, Kwak EL, Cleary JM, Pandya SS (2014). Phase I study of Neratinib in combination with Temsirolimus in patients with human epidermal growth factor receptor 2–dependent and other solid tumors. J Clin Oncol..

[100] Modjtahedi H, Cho BC, Michel MC, Solca F (2014). A comprehensive review of the preclinical efficacy profile of the ErbB family blocker afatinib in cancer. Naunyn Schmiedeberg’s Arch Pharmacol..

[101] Butterworth S, Finlay MRV, Ward RA, Kadambar VK, Chandrashekar RC, Murugan A (2013). 2 - (2, 4, 5 - substituted -anilino) pyrimidine derivatives as egfr modulators useful for treating cancer.

[102] Kobayashi S, Boggon TJ, Dayaram T, Janne PA, Kocher O, Meyerson M (2005). EGFR mutation and resistance of non-small-cell lung cancer to gefitinib. N Engl J Med..

[103] Tang ZH, Lu JJ (2018). Osimertinib resistance in non-small cell lung cancer: mechanisms and therapeutic strategies. Cancer Lett..

[104] Walter AO, Sjin RT, Haringsma HJ, Ohashi K, Sun J, Lee K (2013). Discovery of a mutant-selective covalent inhibitor of EGFR that overcomes T790M-mediated resistance in NSCLC. Cancer Discov..

[105] Sequist LV, Soria JC, Goldman JW, Wakelee HA, Gadgeel SM, Varga A (2015). Rociletinib in EGFR-mutated non-small-cell lung cancer. N Engl J Med..

[106] Kim ES (2016). Olmutinib: first global approval. Drugs..

[107] Hotz B, Keilholz U, Fusi A, Buhr HJ, Hotz HG (2012). In vitro and in vivo antitumor activity of cetuximab in human gastric cancer cell lines in relation to epidermal growth factor receptor (EGFR) expression and mutational phenotype. Gastric Cancer..

[108] Chung CH, Mirakhur B, Chan E, Le Q-T, Berlin J, Morse M (2008). Cetuximab-induced anaphylaxis and IgE specific for galactose-α-1,3-galactose. N Engl J Med..

[109] Freeman DJ, Bush T, Ogbagabriel S, Belmontes B, Juan T, Plewa C (2009). Activity of panitumumab alone or with chemotherapy in non-small cell lung carcinoma cell lines expressing mutant epidermal growth factor receptor. Mol Cancer Ther..

[110] Messersmith WA, Hidalgo M (2007). Panitumumab, a monoclonal anti epidermal growth factor receptor antibody in colorectal cancer: another one or the one?. Clin Cancer Res..

[111] Liu M, Zhang H, Jimenez X, Ludwig D, Witte L, Bohlen P (2004). Identification and characterization of a fully human antibody directed against epidermal growth factor receptor for cancer therapy. Cancer Res..

[112] Genova C, Hirsch FR (2016). Clinical potential of necitumumab in non-small cell lung carcinoma. OncoTargets Ther..

[113] Martinelli E, De Palma R, Orditura M, De Vita F, Ciardiello F (2009). Anti-epidermal growth factor receptor monoclonal antibodies in cancer therapy. Clin Exp Immunol..

[114] Patel D, Lahiji A, Patel S, Franklin M, Jimenez X, Hicklin DJ (2007). Monoclonal antibody cetuximab binds to and down-regulates constitutively activated epidermal growth factor receptor vIII on the cell surface. Anticancer Res..

[115] Cohen MH, Chen H, Shord S, Fuchs C, He K, Zhao H (2013). Approval summary: Cetuximab in combination with cisplatin or carboplatin and 5-fluorouracil for the first-line treatment of patients with recurrent locoregional or metastatic squamous cell head and neck cancer. Oncologist..

[116] Karapetis CS, Khambata-Ford S, Jonker DJ, O'Callaghan CJ, Tu D, Tebbutt NC (2008). K-ras mutations and benefit from cetuximab in advanced colorectal cancer. N Engl J Med..

[117] Di Nicolantonio F, Martini M, Molinari F, Sartore-Bianchi A, Arena S, Saletti P (2008). Wild-type BRAF is required for response to panitumumab or cetuximab in metastatic colorectal cancer. J Clin Oncol..

[118] Amado RG, Wolf M, Peeters M, Van Cutsem E, Siena S, Freeman DJ (2008). Wild-type KRAS is required for Panitumumab efficacy in patients with metastatic colorectal Cancer. J Clin Oncol..

[119] Fitzgerald TL, Lertpiriyapong K, Cocco L, Martelli AM, Libra M, Candido S (2015). Roles of EGFR and KRAS and their downstream signaling pathways in pancreatic cancer and pancreatic cancer stem cells. Adv Biol Regul..

[120] Lievre A, Bachet JB, Le Corre D, Boige V, Landi B, Emile JF (2006). KRAS mutation status is predictive of response to cetuximab therapy in colorectal cancer. Cancer Res..

[121] Cohenuram M, Saif MW (2007). Panitumumab the first fully human monoclonal antibody: from the bench to the clinic. Anti-Cancer Drugs..

[122] Brinkmeyer JK, Moore DC (2018). Necitumumab for the treatment of squamous cell non-small cell lung cancer. J Oncol Pharm Pract..

[123] Paz-Ares L, Mezger J, Ciuleanu TE, Fischer JR, von Pawel J, Provencio M (2015). Necitumumab plus pemetrexed and cisplatin as first-line therapy in patients with stage IV non-squamous non-small-cell lung cancer (INSPIRE): an open-label, randomised, controlled phase 3 study. Lancet Oncol..

[124] Niu G, Chen X (2010). Vascular endothelial growth factor as an anti-angiogenic target for Cancer therapy. Curr Drug Targets..

[125] Yu Y, Lee P, Ke Y, Zhang Y, Yu Q, Lee J (2010). A humanized anti-VEGF rabbit monoclonal antibody inhibits angiogenesis and blocks tumor growth in xenograft models. PLoS One..

[126] Ferrara N, Hillan KJ, Novotny W (2005). Bevacizumab (Avastin), a humanized anti-VEGF monoclonal antibody for cancer therapy. Biochem Biophys Res Commun..

[127] Presta LG, Chen H, Connor SJ, Chisholm V, Meng YG, Krummen L (1997). Humanization of an anti-vascular endothelial growth factor monoclonal antibody for the therapy of solid tumors and other disorders. Cancer Res..

[128] Ellis LM, Hicklin DJ (2008). VEGF-targeted therapy: mechanisms of anti-tumour activity. Nat Rev Cancer..

[129] Zhang F, Tang Z, Hou X, Lennartsson J, Li Y, Koch AW (2009). VEGF-B is dispensable for blood vessel growth but critical for their survival, and VEGF-B targeting inhibits pathological angiogenesis. Proc Natl Acad Sci..

[130] Ferrara N, Gerber H-P, LeCouter J (2003). The biology of VEGF and its receptors. Nat Med..

[131] Carmeliet P (2005). VEGF as a Key Mediator of Angiogenesis in Cancer. Oncology..

[132] Battinelli EM, Markens BA, Kulenthirarajan RA, Machlus KR, Flaumenhaft R, Italiano JE (2014). Anticoagulation inhibits tumor cell–mediated release of platelet angiogenic proteins and diminishes platelet angiogenic response. Blood..

[133] Rak J, Mitsuhashi Y, Bayko L, Filmus J, Shirasawa S, Sasazuki T (1995). Mutant <em>ras</em> oncogenes upregulate VEGF/VPF expression: implications for induction and inhibition of tumor angiogenesis. Cancer Res..

[134] Hanson J, Gorman J, Reese J, Fraizer G (2007). Regulation of vascular endothelial growth factor, VEGF, gene promoter by the tumor suppressor, WT1. Front Biosci..

[135] Tian T, Nan K-J, Wang S-H, Liang X, Lu C-X, Guo H (2010). PTEN regulates angiogenesis and VEGF expression through phosphatase-dependent and -independent mechanisms in HepG2 cells. Carcinogenesis..

[136] Hurwitz H, Saini S (2006). Bevacizumab in the treatment of metastatic colorectal Cancer: safety profile and Management of Adverse Events. Semin Oncol..

[137] Morabito A, De Maio E, Di Maio M, Normanno N, Perrone F (2006). Tyrosine kinase inhibitors of vascular endothelial growth factor receptors in clinical trials: current status and future directions. Oncologist..

[138] Kamba T, McDonald DM (2007). Mechanisms of adverse effects of anti-VEGF therapy for cancer. Br J Cancer..

[139] Escudier B, Eisen T, Stadler WM, Szczylik C, Oudard S, Siebels M (2007). Sorafenib in advanced clear-cell renal-cell carcinoma. N Engl J Med..

[140] Llovet JM, Ricci S, Mazzaferro V, Hilgard P, Gane E, Blanc JF (2008). Sorafenib in advanced hepatocellular carcinoma. N Engl J Med..

[141] Brose MS, Nutting CM, Jarzab B, Elisei R, Siena S, Bastholt L (2014). Sorafenib in radioactive iodine-refractory, locally advanced or metastatic differentiated thyroid cancer: a randomised, double-blind, phase 3 trial. Lancet..

[142] Boudou-Rouquette P, Ropert S, Mir O, Coriat R, Billemont B, Tod M (2012). Variability of Sorafenib toxicity and exposure over time: a pharmacokinetic/Pharmacodynamic analysis. Oncologist..

[143] Chu D, Lacouture ME, Fillos T, Wu S (2008). Risk of hand-foot skin reaction with sorafenib: a systematic review and meta-analysis. Acta Oncol..

[144] Lacouture ME, Wu S, Robert C, Atkins MB, Kong HH, Guitart J (2008). Evolving strategies for the management of hand-foot skin reaction associated with the multitargeted kinase inhibitors sorafenib and sunitinib. Oncologist..

[145] Lipworth AD, Robert C, Zhu AX (2009). Hand-foot syndrome (hand-foot skin reaction, palmar-plantar erythrodysesthesia): focus on sorafenib and sunitinib. Oncology..

[146] Gong L, Giacomini MM, Giacomini C, Maitland ML, Altman RB, Klein TE (2017). PharmGKB summary: Sorafenib pathways. Pharmacogenet Genomics..

[147] Weiner LM, Dhodapkar MV, Ferrone S (2009). Monoclonal Antibodies for Cancer Immunotherapy. Lancet..

[148] Heist RS, Duda DG, Sahani DV, Ancukiewicz M, Fidias P, Sequist LV (2015). Improved tumor vascularization after anti-VEGF therapy with carboplatin and nab-paclitaxel associates with survival in lung cancer. Proc Natl Acad Sci..

[149] Willett CG, Boucher Y, di Tomaso E, Duda DG, Munn LL, Tong RT (2004). Direct evidence that the VEGF-specific antibody bevacizumab has antivascular effects in human rectal cancer. Nat Med..

[150] Ellis LM (2006). Mechanisms of action of bevacizumab as a component of therapy for metastatic colorectal Cancer. Semin Oncol..

[151] Wang L-L, Hu R-C, Dai A-G, Tan S-X (2015). Bevacizumab induces A549 cell apoptosis through the mechanism of endoplasmic reticulum stress in vitro. Int J Clin Exp Pathol..

[152] Selvakumaran M, Yao KS, Feldman MD, O’Dwyer PJ (2008). Antitumor effect of the angiogenesis inhibitor bevacizumab is dependent on susceptibility of tumors to hypoxia-induced apoptosis. Biochem Pharmacol..

[153] Saltz LB, Clarke S, Diaz-Rubio E, Scheithauer W, Figer A, Wong R (2008). Bevacizumab in combination with oxaliplatin-based chemotherapy as first-line therapy in metastatic colorectal cancer: a randomized phase III study. J Clin Oncol..

[154] Johnson DH, Fehrenbacher L, Novotny WF, Herbst RS, Nemunaitis JJ, Jablons DM (2004). Randomized Phase II trial comparing bevacizumab plus carboplatin and paclitaxel with carboplatin and paclitaxel alone in previously untreated locally advanced or metastatic non-small-cell lung Cancer. J Clin Oncol..

[155] Wheler JJ, Janku F, Falchook GS, Jackson TL, Fu S, Naing A (2014). Phase I study of anti-VEGF monoclonal antibody bevacizumab and histone deacetylase inhibitor valproic acid in patients with advanced cancers. Cancer Chemother Pharmacol..

[156] Kreisl TN, Kim L, Moore K, Duic P, Royce C, Stroud I (2009). Phase II trial of single-agent bevacizumab followed by bevacizumab plus irinotecan at tumor progression in recurrent glioblastoma. J Clin Oncol..

[157] Escudier B, Pluzanska A, Koralewski P, Ravaud A, Bracarda S, Szczylik C (2007). Bevacizumab plus interferon alfa-2a for treatment of metastatic renal cell carcinoma: a randomised, double-blind phase III trial. Lancet..

[158] Fuh KC, Secord AA, Bevis KS, Huh W, ElNaggar A, Blansit K (2015). Comparison of bevacizumab alone or with chemotherapy in recurrent ovarian cancer patients. Gynecol Oncol..

[159] Cohen MH, Gootenberg J, Keegan P, Pazdur R (2007). FDA drug approval summary: bevacizumab (Avastin) plus carboplatin and paclitaxel as first-line treatment of advanced/metastatic recurrent nonsquamous non-small cell lung cancer. Oncologist..

[160] Kabbinavar FF, Schulz J, McCleod M, Patel T, Hamm JT, Hecht JR (2005). Addition of bevacizumab to bolus fluorouracil and leucovorin in first-line metastatic colorectal cancer: results of a randomized phase II trial. J Clin Oncol..

[161] Fuchs CS, Tomasek J, Yong CJ, Dumitru F, Passalacqua R, Goswami C (2014). Ramucirumab monotherapy for previously treated advanced gastric or gastro-oesophageal junction adenocarcinoma (REGARD): an international, randomised, multicentre, placebo-controlled, phase 3 trial. Lancet..

[162] Wilke H, Muro K, Van Cutsem E, Oh S-C, Bodoky G, Shimada Y (2014). Ramucirumab plus paclitaxel versus placebo plus paclitaxel in patients with previously treated advanced gastric or gastro-oesophageal junction adenocarcinoma (RAINBOW): a double-blind, randomised phase 3 trial. Lancet Oncol..

[163] Garon EB, Ciuleanu T-E, Arrieta O, Prabhash K, Syrigos KN, Goksel T (2014). Ramucirumab plus docetaxel versus placebo plus docetaxel for second-line treatment of stage IV non-small-cell lung cancer after disease progression on platinum-based therapy (REVEL): a multicentre, double-blind, randomised phase 3 trial. Lancet..

[164] Vennepureddy A, Singh P, Rastogi R, Atallah JP, Terjanian T (2017). Evolution of ramucirumab in the treatment of cancer - a review of literature. J Oncol Pharm Pract..

[165] Clarke JM, Hurwitz HI (2013). Targeted inhibition of VEGF receptor 2: an update on ramucirumab. Expert Opin Biol Ther..

[166] Van Cutsem E, Tabernero J, Lakomy R, Prenen H, Prausová J, Macarulla T (2012). Addition of Aflibercept to fluorouracil, Leucovorin, and irinotecan improves survival in a Phase III randomized trial in patients with metastatic colorectal Cancer previously treated with an Oxaliplatin-based regimen. J Clin Oncol..

[167] Cook KM, Figg WD (2010). Angiogenesis inhibitors – current strategies and future prospects. CA Cancer J Clin..

[168] Tang PA, Moore MJ (2013). Aflibercept in the treatment of patients with metastatic colorectal cancer: latest findings and interpretations. Ther Adv Gastroenterol..

[169] Bibeau F, Goldman-Levy G, Artru P, Desrame J, Lledo G, Mithieux F (2016). P-156Pathologic response of liver metastases from colorectal cancer after chemotherapy and aflibercept: initial report of 23 cases from 9 patients. Ann Oncol.

[170] Tannock IF, Fizazi K, Ivanov S, Karlsson CT, Fléchon A, Skoneczna I (2013). Aflibercept versus placebo in combination with docetaxel and prednisone for treatment of men with metastatic castration-resistant prostate cancer (VENICE): a phase 3, double-blind randomised trial. Lancet Oncol..

[171] Gaya A, Tse V (2012). A preclinical and clinical review of aflibercept for the management of cancer. Cancer Treat Rev..

[172] Haller JA, Boyer DS, Heier JS, Brown DM, Clark L, RVEGF V (2011). Trap-Eye In CRVO: Primary Endpoint Results Of The Phase 3 COPERNICUS Study. Invest Ophthalmol Vis Sci..

[173] Li L, Ma BBY (2014). Colorectal cancer in Chinese patients: current and emerging treatment options. OncoTargets Ther..

[174] Bordonaro R, Sobrero AF, Frassineti L, Ciuffreda L, Aprile G, Thomas AL (2014). Ziv-aflibercept in combination with FOLFIRI for second-line treatment of patients with metastatic colorectal cancer (mCRC): Interim safety data from the global aflibercept safety and quality-of-life program (ASQoP and AFEQT studies) in patients ≥65. J Clin Oncol.

[175] Gotlieb WH, Amant F, Advani S, Goswami C, Hirte H, Provencher D (2012). Intravenous aflibercept for treatment of recurrent symptomatic malignant ascites in patients with advanced ovarian cancer: a phase 2, randomised, double-blind, placebo-controlled study. Lancet Oncol..

[176] Rougier P, Riess H, Manges R, Karasek P, Humblet Y, Barone C (2013). Randomised, placebo-controlled, double-blind, parallel-group phase III study evaluating aflibercept in patients receiving first-line treatment with gemcitabine for metastatic pancreatic cancer. Eur J Cancer..

[177] Sasich LD, Sukkari SR (2012). The US FDAs withdrawal of the breast cancer indication for Avastin (bevacizumab). Saudi Pharmaceutical J..

[178] Yarden Y (2001). The EGFR family and its ligands in human cancer. Eur J Cancer..

[179] Moasser MM (2007). The oncogene HER2; its signaling and transforming functions and its role in human cancer pathogenesis. Oncogene..

[180] Vu T, Claret FX (2012). Trastuzumab: updated mechanisms of action and resistance in breast Cancer. Front Oncol..

[181] Slamon DJ, Godolphin W, Jones LA, Holt JA, Wong SG, Keith DE (1989). Studies of the HER-2/neu proto-oncogene in human breast and ovarian cancer. Science..

[182] Slamon DJ, Leyland-Jones B, Shak S, Fuchs H, Paton V, Bajamonde A (2001). Use of chemotherapy plus a monoclonal antibody against HER2 for metastatic breast Cancer that overexpresses HER2. N Engl J Med..

[183] Hudis CA (2007). Trastuzumab — mechanism of action and use in clinical practice. N Engl J Med..

[184] Zazo S, González-Alonso P, Martín-Aparicio E, Chamizo C, Cristóbal I, Arpí O (2016). Generation, characterization, and maintenance of trastuzumab-resistant HER2+ breast cancer cell lines. Am J Cancer Res..

[185] Carter P, Presta L, Gorman CM, Ridgway JB, Henner D, Wong WL (1992). Humanization of an anti-p185HER2 antibody for human cancer therapy. Proc Natl Acad Sci..

[186] Arnould L, Gelly M, Penault-Llorca F, Benoit L, Bonnetain F, Migeon C (2006). Trastuzumab-based treatment of HER2-positive breast cancer: an antibody-dependent cellular cytotoxicity mechanism?. Br J Cancer..

[187] Clynes RA, Towers TL, Presta LG, Ravetch JV (2000). Inhibitory fc receptors modulate in vivo cytoxicity against tumor targets. Nat Med..

[188] Junttila TT, Akita RW, Parsons K, Fields C, Lewis Phillips GD, Friedman LS (2009). Ligand-independent HER2/HER3/PI3K complex is disrupted by Trastuzumab and is effectively inhibited by the PI3K inhibitor GDC-0941. Cancer Cell..

[189] Baselga J (2001). Phase I and II clinical trials of trastuzumab. Ann Oncol.

[190] Vogel CL, Cobleigh MA, Tripathy D, Gutheil JC, Harris LN, Fehrenbacher L (2002). Efficacy and safety of trastuzumab as a single agent in first-line treatment of HER2-overexpressing metastatic breast cancer. J Clin Oncol..

[191] Strasser-Weippl K, Horick N, Smith IE, O’Shaughnessy J, Ejlertsen B, Boyle F (2015). Long-term hazard of recurrence in HER2+ breast cancer patients untreated with anti-HER2 therapy. Breast Cancer Res..

[192] Zhang H, Wang Y, Wu Y, Jiang X, Tao Y, Yao Y (2017). Therapeutic potential of an anti-HER2 single chain antibody-DM1 conjugates for the treatment of HER2-positive cancer. Signal Transduct Target Ther..

[193] Junttila TT, Li G, Parsons K, Phillips GL, Sliwkowski MX (2011). Trastuzumab-DM1 (T-DM1) retains all the mechanisms of action of trastuzumab and efficiently inhibits growth of lapatinib insensitive breast cancer. Breast Cancer Res Treat..

[194] Krop I, Winer EP (2014). Trastuzumab emtansine: a novel antibody-drug conjugate for HER2-positive breast cancer. Clin Cancer Res..

[195] Patel TA, Ensor J, Creamer S, Rodriguez AA, Niravath PA, Darcourt JG (2018). Care 001: Multicenter randomized open label phase II trial of neoadjuvant trastuzumabemtansine (T-DM1) in combination with lapatinib and nab-paclitaxel compared with paclitaxel, trastuzumab and pertuzumab in HER 2 neu over-expressed breast cancer patients (TEAL study). J Clin Oncol.

[196] Verma S, Miles D, Gianni L, Krop IE, Welslau M, Baselga J (2012). Trastuzumab emtansine for HER2-positive advanced breast cancer. N Engl J Med..

[197] Dillon RL, Chooniedass S, Premsukh A, Adams GP, Entwistle J, MacDonald GC (2016). Trastuzumab-deBouganin conjugate overcomes multiple mechanisms of T-DM1 drug resistance. J Immunother..

[198] Capelan M, Pugliano L, De Azambuja E, Bozovic I, Saini KS, Sotiriou C (2013). Pertuzumab: new hope for patients with HER2-positive breast cancer. Ann Oncol..

[199] Scheuer W, Friess T, Burtscher H, Bossenmaier B, Endl J, Hasmann M (2009). Strongly enhanced antitumor activity of Trastuzumab and Pertuzumab combination treatment on HER2-positive human xenograft tumor models. Cancer Res..

[200] Baselga J, Cortes J, Kim SB, Im SA, Hegg R, Im YH (2012). Pertuzumab plus trastuzumab plus docetaxel for metastatic breast cancer. N Engl J Med..

[201] Geyer CE, Forster J, Lindquist D, Chan S, Romieu CG, Pienkowski T (2006). Lapatinib plus capecitabine for HER2-positive advanced breast cancer. N Engl J Med..

[202] D’Amato V, Raimondo L, Formisano L, Giuliano M, De Placido S, Rosa R (2015). Mechanisms of lapatinib resistance in HER2-driven breast cancer. Cancer Treat Rev..

[203] Medina PJ, Goodin S (2008). Lapatinib: a dual inhibitor of human epidermal growth factor receptor tyrosine kinases. Clin Ther..

[204] Cameron D, Casey M, Press M, Lindquist D, Pienkowski T, Romieu CG (2008). A phase III randomized comparison of lapatinib plus capecitabine versus capecitabine alone in women with advanced breast cancer that has progressed on trastuzumab: updated efficacy and biomarker analyses. Breast Cancer Res Treat..

[205] Hallberg B, Palmer RH (2016). The role of the ALK receptor in cancer biology. Ann Oncol.

[206] Hallberg B, Palmer RH (2013). Mechanistic insight into ALK receptor tyrosine kinase in human cancer biology. Nat Rev Cancer..

[207] Chia PL, Mitchell P, Dobrovic A, John T (2014). Prevalence and natural history of ALK positive non-small-cell lung cancer and the clinical impact of targeted therapy with ALK inhibitors. Clin Epidemiol..

[208] Soda M, Choi YL, Enomoto M, Takada S, Yamashita Y, Ishikawa S (2007). Identification of the transforming EML4-ALK fusion gene in non-small-cell lung cancer. Nature..

[209] Shaw AT, Yeap BY, Mino-Kenudson M, Digumarthy SR, Costa DB, Heist RS (2009). Clinical features and outcome of patients with non-small-cell lung cancer who harbor EML4-ALK. J Clin Oncol..

[210] Alshareef Abdulraheem (2017). Novel Molecular Challenges in Targeting Anaplastic Lymphoma Kinase in ALK-Expressing Human Cancers. Cancers.

[211] Kodama T, Tsukaguchi T, Yoshida M, Kondoh O, Sakamoto H (2014). Selective ALK inhibitor alectinib with potent antitumor activity in models of crizotinib resistance. Cancer Lett..

[212] Cui JJ, Tran-Dube M, Shen H, Nambu M, Kung PP, Pairish M (2011). Structure based drug design of crizotinib (PF-02341066), a potent and selective dual inhibitor of mesenchymal-epithelial transition factor (c-MET) kinase and anaplastic lymphoma kinase (ALK). J Med Chem..

[213] Friboulet L, Li N, Katayama R, Lee CC, Gainor JF, Crystal AS (2014). The ALK inhibitor ceritinib overcomes crizotinib resistance in non-small cell lung cancer. Cancer Discov..

[214] Zhang S, Anjum R, Squillace R, Nadworny S, Zhou T, Keats J (2016). The potent ALK inhibitor Brigatinib (AP26113) overcomes mechanisms of resistance to first- and second-generation ALK inhibitors in preclinical models. Clin Cancer Res..

[215] Camidge DR, Bang YJ, Kwak EL, Iafrate AJ, Varella-Garcia M, Fox SB (2012). Activity and safety of crizotinib in patients with ALK-positive non-small-cell lung cancer: updated results from a phase 1 study. Lancet Oncol..

[216] Kim D-W, Ahn M-J, Shi Y, Pas TMD, Yang P-C, Riely GJ (2012). Results of a global phase II study with crizotinib in advanced ALK-positive non-small cell lung cancer (NSCLC). J Clin Oncol.

[217] Shaw AT, Kim DW, Nakagawa K, Seto T, Crino L, Ahn MJ (2013). Crizotinib versus chemotherapy in advanced ALK-positive lung cancer. N Engl J Med..

[218] Solomon BJ, Mok T, Kim DW, Wu YL, Nakagawa K, Mekhail T (2014). First-line crizotinib versus chemotherapy in ALK-positive lung cancer. N Engl J Med..

[219] Wu J, Savooji J, Liu D (2016). Second- and third-generation ALK inhibitors for non-small cell lung cancer. J Hematol Oncol..

[220] Shaw AT, Engelman JA (2014). Ceritinib in ALK-rearranged non-small-cell lung cancer. N Engl J Med..

[221] Costa DB, Shaw AT, Ou SH, Solomon BJ, Riely GJ, Ahn MJ (2015). Clinical experience with Crizotinib in patients with advanced ALK-rearranged non-small-cell lung Cancer and brain metastases. J Clin Oncol..

[222] Heuckmann JM, Holzel M, Sos ML, Heynck S, Balke-Want H, Koker M (2011). ALK mutations conferring differential resistance to structurally diverse ALK inhibitors. Clin Cancer Res..

[223] Toyokawa G, Seto T (2015). Updated evidence on the mechanisms of resistance to ALK inhibitors and strategies to overcome such resistance: clinical and preclinical data. Oncol Res Treat..

[224] Choi YL, Soda M, Yamashita Y, Ueno T, Takashima J, Nakajima T (2010). EML4-ALK mutations in lung cancer that confer resistance to ALK inhibitors. N Engl J Med..

[225] Soria JC, Tan DSW, Chiari R, Wu YL, Paz-Ares L, Wolf J (2017). First-line ceritinib versus platinum-based chemotherapy in advanced ALK-rearranged non-small-cell lung cancer (ASCEND-4): a randomised, open-label, phase 3 study. Lancet..

[226] Kim DW, Mehra R, Tan DS, Felip E, Chow LQ, Camidge DR (2016). Activity and safety of ceritinib in patients with ALK-rearranged non-small-cell lung cancer (ASCEND-1): updated results from the multicentre, open-label, phase 1 trial. Lancet Oncol..

[227] Crinò L, Ahn M-J, De Marinis F, Groen HJM, Wakelee H, Hida T (2016). Multicenter Phase II study of whole-body and intracranial activity with Ceritinib in patients with ALK-rearranged non–small-cell lung Cancer previously treated with chemotherapy and Crizotinib: results from ASCEND-2. J Clin Oncol..

[228] Felip E, Orlov S, Park K, Yu C-J, Tsai C-M, Nishio M (2015). ASCEND-3: A single-arm, open-label, multicenter phase II study of ceritinib in ALKi-naïve adult patients (pts) with ALK-rearranged (ALK+) non-small cell lung cancer (NSCLC). J Clin Oncol.

[229] Soria J-C, Tan DSW, Chiari R, Wu Y-L, Paz-Ares L, Wolf J (2017). First-line ceritinib versus platinum-based chemotherapy in advanced <em>ALK</em>−rearranged non-small-cell lung cancer (ASCEND-4): a randomised, open-label, phase 3 study. Lancet..

[230] Shaw AT, Kim TM, Crino L, Gridelli C, Kiura K, Liu G (2017). Ceritinib versus chemotherapy in patients with ALK-rearranged non-small-cell lung cancer previously given chemotherapy and crizotinib (ASCEND-5): a randomised, controlled, open-label, phase 3 trial. Lancet Oncol..

[231] Cho BC, Kim DW, Bearz A, Laurie SA, McKeage M, Borra G (2017). ASCEND-8: a randomized Phase 1 study of Ceritinib, 450 mg or 600 mg, taken with a low-fat meal versus 750 mg in fasted state in patients with anaplastic lymphoma kinase (ALK)-rearranged metastatic non-small cell lung Cancer (NSCLC). J Thorac Oncol..

[232] Sakamoto H, Tsukaguchi T, Hiroshima S, Kodama T, Kobayashi T, Fukami TA (2011). CH5424802, a selective ALK inhibitor capable of blocking the resistant gatekeeper mutant. Cancer Cell..

[233] Seto T, Kiura K, Nishio M, Nakagawa K, Maemondo M, Inoue A (2013). CH5424802 (RO5424802) for patients with ALK-rearranged advanced non-small-cell lung cancer (AF-001JP study): a single-arm, open-label, phase 1-2 study. Lancet Oncol..

[234] Gadgeel SM, Gandhi L, Riely GJ, Chiappori AA, West HL, Azada MC (2014). Safety and activity of alectinib against systemic disease and brain metastases in patients with crizotinib-resistant ALK-rearranged non-small-cell lung cancer (AF-002JG): results from the dose-finding portion of a phase 1/2 study. Lancet Oncol..

[235] Peters S, Camidge DR, Shaw AT, Gadgeel S, Ahn JS, Kim DW (2017). Alectinib versus Crizotinib in untreated ALK-positive non-small-cell lung Cancer. N Engl J Med..

[236] Shaw AT, Felip E, Bauer TM, Besse B, Navarro A, Postel-Vinay S (2017). Lorlatinib in non-small-cell lung cancer with ALK or ROS1 rearrangement: an international, multicentre, open-label, single-arm first-in-man phase 1 trial. Lancet Oncol..

[237] Moelling K, Heimann B, Beimling P, Rapp UR, Sander T (1984). Serine- and threonine-specific protein kinase activities of purified gag-mil and gag-raf proteins. Nature..

[238] Rahman MA, Salajegheh A, Smith RA, Lam AK (2013). B-Raf mutation: a key player in molecular biology of cancer. Exp Mol Pathol..

[239] Zebisch A, Troppmair J (2006). Back to the roots: the remarkable RAF oncogene story. Cell Mol Life Sci..

[240] Chambard JC, Lefloch R, Pouyssegur J, Lenormand P (2007). ERK implication in cell cycle regulation. Biochim Biophys Acta..

[241] Holderfield M, Deuker MM, McCormick F, McMahon M (2014). Targeting RAF kinases for cancer therapy: BRAF-mutated melanoma and beyond. Nat Rev Cancer..

[242] Davies H, Bignell GR, Cox C, Stephens P, Edkins S, Clegg S (2002). Mutations of the BRAF gene in human cancer. Nature..

[243] Kainthla R, Kim KB, Falchook GS (2014). Dabrafenib for treatment of BRAF-mutant melanoma. Pharmgenomic Pers Med..

[244] Pritchard AL, Hayward NK (2013). Molecular pathways: mitogen-activated protein kinase pathway mutations and drug resistance. Clin Cancer Res..

[245] Pakneshan S, Salajegheh A, Smith RA, Lam AK (2013). Clinicopathological relevance of BRAF mutations in human cancer. Pathology..

[246] Wan PT, Garnett MJ, Roe SM, Lee S, Niculescu-Duvaz D, Good VM (2004). Mechanism of activation of the RAF-ERK signaling pathway by oncogenic mutations of B-RAF. Cell..

[247] Tan YH, Liu Y, Eu KW, Ang PW, Li WQ, Salto-Tellez M (2008). Detection of BRAF V600E mutation by pyrosequencing. Pathology..

[248] Ikenoue T, Hikiba Y, Kanai F, Tanaka Y, Imamura J, Imamura T (2003). Functional analysis of mutations within the kinase activation segment of B-Raf in human colorectal tumors. Cancer Res..

[249] Roskoski R (2010). RAF protein-serine/threonine kinases: structure and regulation. Biochem Biophys Res Commun..

[250] Wellbrock C, Karasarides M, Marais R (2004). The RAF proteins take Centre stage. Nat Rev Mol Cell Biol..

[251] Menzies AM, Haydu LE, Visintin L, Carlino MS, Howle JR, Thompson JF (2012). Distinguishing clinicopathologic features of patients with V600E and V600K BRAF-mutant metastatic melanoma. Clin Cancer Res..

[252] Li Y, Li W (2017). BRAF mutation is associated with poor clinicopathological outcomes in colorectal cancer: a meta-analysis. Saudi J Gastroenterol..

[253] Rahman MA, Salajegheh A, Smith RA, Lam AK (2014). BRAF inhibitors: from the laboratory to clinical trials. Crit Rev Oncol Hematol..

[254] Levy JB, Pauloski N, Braun D, Derome M, Jordan J, Shi H (2006). Analysis of transcription and protein expression changes in the 786-O human renal cell carcinoma tumor xenograft model in response to treatment with the multi-kinase inhibitor sorafenib (BAY 43-9006). Cancer Res..

[255] Carlomagno F, Anaganti S, Guida T, Salvatore G, Troncone G, Wilhelm SM (2006). BAY 43-9006 inhibition of oncogenic RET mutants. J Natl Cancer Inst..

[256] Casadei Gardini A, Chiadini E, Faloppi L, Marisi G, Delmonte A, Scartozzi M (2016). Efficacy of sorafenib in BRAF-mutated non-small-cell lung cancer (NSCLC) and no response in synchronous BRAF wild type-hepatocellular carcinoma: a case report. BMC Cancer..

[257] Eisen T, Ahmad T, Flaherty KT, Gore M, Kaye S, Marais R (2006). Sorafenib in advanced melanoma: a Phase II randomised discontinuation trial analysis. Br J Cancer..

[258] Abou-Alfa GK, Schwartz L, Ricci S, Amadori D, Santoro A, Figer A (2006). Phase II study of sorafenib in patients with advanced hepatocellular carcinoma. J Clin Oncol..

[259] Pécuchet N, Lebbe C, Mir O, Billemont B, Blanchet B, Franck N (2012). Sorafenib in advanced melanoma: a critical role for pharmacokinetics?. Br J Cancer..

[260] Morris V, Kopetz S (2013). BRAF inhibitors in clinical oncology. F1000Prime Rep..

[261] Bollag G, Hirth P, Tsai J, Zhang J, Ibrahim PN, Cho H (2010). Clinical efficacy of a RAF inhibitor needs broad target blockade in BRAF-mutant melanoma. Nature..

[262] Zambon A, Niculescu-Duvaz I, Niculescu-Duvaz D, Marais R, Springer CJ (2012). Small molecule inhibitors of BRAF in clinical trials. Bioorg Med Chem Lett..

[263] Yang H, Higgins B, Kolinsky K, Packman K, Go Z, Iyer R (2010). RG7204 (PLX4032), a selective BRAFV600E inhibitor, displays potent antitumor activity in preclinical melanoma models. Cancer Res..

[264] Laquerre S, Arnone M, Moss K, Yang J, Fisher K, Kane-Carson LS (2009). Abstract B88: A selective Raf kinase inhibitor induces cell death and tumor regression of human cancer cell lines encoding B-Raf<sup>V600E</sup> mutation. Mol Cancer Ther.

[265] Gibney GT, Zager JS (2013). Clinical development of dabrafenib in BRAF mutant melanoma and other malignancies. Expert Opin Drug Metab Toxicol..

[266] Gentilcore G, Madonna G, Mozzillo N, Ribas A, Cossu A, Palmieri G (2013). Effect of dabrafenib on melanoma cell lines harbouring the BRAF(V600D/R) mutations. BMC Cancer..

[267] Subbiah V, Kreitman RJ, Wainberg ZA, Cho JY, Schellens JHM, Soria JC (2017). Dabrafenib and Trametinib treatment in patients with locally advanced or metastatic BRAF V600–mutant anaplastic thyroid Cancer. J Clin Oncol..

[268] Wilhelm SM, Dumas J, Adnane L, Lynch M, Carter CA, Schutz G (2011). Regorafenib (BAY 73-4506): a new oral multikinase inhibitor of angiogenic, stromal and oncogenic receptor tyrosine kinases with potent preclinical antitumor activity. Int J Cancer..

[269] Personeni N, Pressiani T, Santoro A, Rimassa L (2018). Regorafenib in hepatocellular carcinoma: latest evidence and clinical implications. Drugs Context..

[270] Krishnamoorthy SK, Relias V, Sebastian S, Jayaraman V, Saif MW (2015). Management of regorafenib-related toxicities: a review. Ther Adv Gastroenterol..

[271] Huang T, Karsy M, Zhuge J, Zhong M, Liu D (2013). B-Raf and the inhibitors: from bench to bedside. J Hematol Oncol..

[272] Alcala AM, Flaherty KT (2012). BRAF inhibitors for the treatment of metastatic melanoma: clinical trials and mechanisms of resistance. Clin Cancer Res..

[273] Trunzer K, Pavlick AC, Schuchter L, Gonzalez R, McArthur GA, Hutson TE (2013). Pharmacodynamic effects and mechanisms of resistance to vemurafenib in patients with metastatic melanoma. J Clin Oncol..

[274] Corcoran RB, Settleman J, Engelman JA (2011). Potential therapeutic strategies to overcome acquired resistance to BRAF or MEK inhibitors in BRAF mutant cancers. Oncotarget..

[275] Smith-Garvin JE, Koretzky GA, Jordan MS (2009). T cell activation. Annu Rev Immunol..

[276] Pardoll DM (2012). The blockade of immune checkpoints in cancer immunotherapy. Nat Rev Cancer..

[277] Parry RV, Chemnitz JM, Frauwirth KA, Lanfranco AR, Braunstein I, Kobayashi SV (2005). CTLA-4 and PD-1 receptors inhibit T-cell activation by distinct mechanisms. Mol Cell Biol..

[278] Anderson AC, Joller N, Kuchroo VK (2016). Lag-3, Tim-3, and TIGIT: co-inhibitory receptors with specialized functions in immune regulation. Immunity..

[279] Blackburn SD, Shin H, Haining WN, Zou T, Workman CJ, Polley A (2009). Coregulation of CD8+ T cell exhaustion by multiple inhibitory receptors during chronic viral infection. Nat Immunol..

[280] Seidel JA, Otsuka A, Kabashima K (2018). Anti-PD-1 and anti-CTLA-4 therapies in Cancer: mechanisms of action, efficacy, and limitations. Front Oncol..

[281] Zarour HM (2016). Reversing T-cell dysfunction and exhaustion in Cancer. Clin Cancer Res..

[282] Day CL, Kaufmann DE, Kiepiela P, Brown JA, Moodley ES, Reddy S (2006). PD-1 expression on HIV-specific T cells is associated with T-cell exhaustion and disease progression. Nature..

[283] Baitsch L, Baumgaertner P, Devevre E, Raghav SK, Legat A, Barba L (2011). Exhaustion of tumor-specific CD8(+) T cells in metastases from melanoma patients. J Clin Invest..

[284] Leach DR, Krummel MF, Allison JP (1996). Enhancement of antitumor immunity by CTLA-4 blockade. Science..

[285] Hirano F, Kaneko K, Tamura H, Dong H, Wang S, Ichikawa M (2005). Blockade of B7-H1 and PD-1 by monoclonal antibodies potentiates cancer therapeutic immunity. Cancer Res..

[286] Greenwald RJ, Freeman GJ, Sharpe AH (2005). The B7 family revisited. Annu Rev Immunol..

[287] Chan DV, Gibson HM, Aufiero BM, Wilson AJ, Hafner MS, Mi QS (2014). Differential CTLA-4 expression in human CD4+ versus CD8+ T cells is associated with increased NFAT1 and inhibition of CD4+ proliferation. Genes Immun..

[288] Hodi FS, Mihm MC, Soiffer RJ, Haluska FG, Butler M, Seiden MV (2003). Biologic activity of cytotoxic T lymphocyte-associated antigen 4 antibody blockade in previously vaccinated metastatic melanoma and ovarian carcinoma patients. Proc Natl Acad Sci U S A..

[289] Ribas A, Hanson DC, Noe DA, Millham R, Guyot DJ, Bernstein SH (2007). Tremelimumab (CP-675,206), a cytotoxic T lymphocyte associated antigen 4 blocking monoclonal antibody in clinical development for patients with cancer. Oncologist..

[290] Harris SJ, Brown J, Lopez J, Yap TA (2016). Immuno-oncology combinations: raising the tail of the survival curve. Cancer Biol Med..

[291] Tremelimumab (2010). Drugs RD..

[292] Martens A, Wistuba-Hamprecht K, Yuan J, Postow MA, Wong P, Capone M (2016). Increases in absolute lymphocytes and circulating CD4+ and CD8+ T cells are associated with positive clinical outcome of melanoma patients treated with Ipilimumab. Clin Cancer Res..

[293] Hodi FS, O'Day SJ, McDermott DF, Weber RW, Sosman JA, Haanen JB (2010). Improved survival with ipilimumab in patients with metastatic melanoma. N Engl J Med..

[294] Peggs KS, Quezada SA, Korman AJ, Allison JP (2006). Principles and use of anti-CTLA4 antibody in human cancer immunotherapy. Curr Opin Immunol..

[295] Dong H, Zhu G, Tamada K, Chen L (1999). B7-H1, a third member of the B7 family, co-stimulates T-cell proliferation and interleukin-10 secretion. Nat Med..

[296] Freeman GJ, Long AJ, Iwai Y, Bourque K, Chernova T, Nishimura H (2000). Engagement of the PD-1 immunoinhibitory receptor by a novel B7 family member leads to negative regulation of lymphocyte activation. J Exp Med..

[297] Viricel C, Ahmed M, Barakat K (2015). Human PD-1 binds differently to its human ligands: a comprehensive modeling study. J Mol Graph Modell..

[298] Iwai Y, Ishida M, Tanaka Y, Okazaki T, Honjo T, Minato N (2002). Involvement of PD-L1 on tumor cells in the escape from host immune system and tumor immunotherapy by PD-L1 blockade. Proc Natl Acad Sci U S A..

[299] Dong H, Strome SE, Salomao DR, Tamura H, Hirano F, Flies DB (2002). Tumor-associated B7-H1 promotes T-cell apoptosis: a potential mechanism of immune evasion. Nat Med..

[300] Tan S, Zhang CW, Gao GF (2016). Seeing is believing: anti-PD-1/PD-L1 monoclonal antibodies in action for checkpoint blockade tumor immunotherapy. Signal Transduct Target Ther..

[301] Garon EB, Rizvi NA, Hui R, Leighl N, Balmanoukian AS, Eder JP (2015). Pembrolizumab for the treatment of non-small-cell lung cancer. N Engl J Med..

[302] Robert C, Ribas A, Wolchok JD, Hodi FS, Hamid O, Kefford R (2014). Anti-programmed-death-receptor-1 treatment with pembrolizumab in ipilimumab-refractory advanced melanoma: a randomised dose-comparison cohort of a phase 1 trial. Lancet..

[303] Chuk MK, Chang JT, Theoret MR, Sampene E, He K, Weis SL (2017). FDA approval summary: accelerated approval of Pembrolizumab for second-line treatment of metastatic melanoma. Clin Cancer Res..

[304] Balar AV, Castellano D, O'Donnell PH, Grivas P, Vuky J, Powles T (2017). First-line pembrolizumab in cisplatin-ineligible patients with locally advanced and unresectable or metastatic urothelial cancer (KEYNOTE-052): a multicentre, single-arm, phase 2 study. Lancet Oncol..

[305] Alley EW, Lopez J, Santoro A, Morosky A, Saraf S, Piperdi B (2017). Clinical safety and activity of pembrolizumab in patients with malignant pleural mesothelioma (KEYNOTE-028): preliminary results from a non-randomised, open-label, phase 1b trial. Lancet Oncol..

[306] Le DT, Durham JN, Smith KN, Wang H, Bartlett BR, Aulakh LK (2017). Mismatch repair deficiency predicts response of solid tumors to PD-1 blockade. Science..

[307] Fuchs CS, Doi T, Jang RW, Muro K, Satoh T, Machado M (2018). Safety and efficacy of Pembrolizumab monotherapy in patients with previously treated advanced gastric and gastroesophageal junction Cancer: Phase 2 clinical KEYNOTE-059 trial. JAMA Oncology..

[308] Chow LQM, Haddad R, Gupta S, Mahipal A, Mehra R, Tahara M (2016). Antitumor activity of Pembrolizumab in biomarker-unselected patients with recurrent and/or metastatic head and neck squamous cell carcinoma: results from the Phase Ib KEYNOTE-012 expansion cohort. J Clin Oncol..

[309] Gandhi L, Rodriguez-Abreu D, Gadgeel S, Esteban E, Felip E, De Angelis F (2018). Pembrolizumab plus chemotherapy in metastatic non-small-cell lung Cancer. N Engl J Med..

[310] Brahmer JR, Drake CG, Wollner I, Powderly JD, Picus J, Sharfman WH (2010). Phase I study of single-agent anti-programmed death-1 (MDX-1106) in refractory solid tumors: safety, clinical activity, pharmacodynamics, and immunologic correlates. J Clin Oncol..

[311] Guo L, Zhang H, Chen B (2017). Nivolumab as programmed Death-1 (PD-1) inhibitor for targeted immunotherapy in tumor. J Cancer..

[312] Weber JS, D'Angelo SP, Minor D, Hodi FS, Gutzmer R, Neyns B (2015). Nivolumab versus chemotherapy in patients with advanced melanoma who progressed after anti-CTLA-4 treatment (CheckMate 037): a randomised, controlled, open-label, phase 3 trial. Lancet Oncol..

[313] Hazarika M, Chuk MK, Theoret MR, Mushti S, He K, Weis SL (2017). U.S. FDA approval summary: Nivolumab for treatment of Unresectable or metastatic melanoma following progression on Ipilimumab. Clin Cancer Res..

[314] Hasan Ali O, Diem S, Markert E, Jochum W, Kerl K, French LE (2016). Characterization of nivolumab-associated skin reactions in patients with metastatic non-small cell lung cancer. Oncoimmunology..

[315] Kroschinsky F, Stölzel F, von Bonin S, Beutel G, Kochanek M, Kiehl M (2017). New drugs, new toxicities: severe side effects of modern targeted and immunotherapy of cancer and their management. Crit Care..

[316] Borghaei H, Paz-Ares L, Horn L, Spigel DR, Steins M, Ready NE (2015). Nivolumab versus docetaxel in advanced nonsquamous non-small-cell lung Cancer. N Engl J Med..

[317] Overman MJ, McDermott R, Leach JL, Lonardi S, Lenz HJ, Morse MA (2017). Nivolumab in patients with metastatic DNA mismatch repair-deficient or microsatellite instability-high colorectal cancer (CheckMate 142): an open-label, multicentre, phase 2 study. Lancet Oncol..

[318] Ansell SM, Lesokhin AM, Borrello I, Halwani A, Scott EC, Gutierrez M (2015). PD-1 blockade with nivolumab in relapsed or refractory Hodgkin's lymphoma. N Engl J Med..

[319] Younes A, Santoro A, Shipp M, Zinzani PL, Timmerman JM, Ansell S (2016). Nivolumab for classical Hodgkin's lymphoma after failure of both autologous stem-cell transplantation and brentuximab vedotin: a multicentre, multicohort, single-arm phase 2 trial. Lancet Oncol..

[320] El-Khoueiry AB, Sangro B, Yau T, Crocenzi TS, Kudo M, Hsu C (2017). Nivolumab in patients with advanced hepatocellular carcinoma (CheckMate 040): an open-label, non-comparative, phase 1/2 dose escalation and expansion trial. Lancet..

[321] Sharma P, Callahan MK, Bono P, Kim J, Spiliopoulou P, Calvo E (2016). Nivolumab monotherapy in recurrent metastatic urothelial carcinoma (CheckMate 032): a multicentre, open-label, two-stage, multi-arm, phase 1/2 trial. Lancet Oncol..

[322] Larkin J, Hodi FS, Wolchok JD (2015). Combined Nivolumab and Ipilimumab or monotherapy in untreated melanoma. N Engl J Med..

[323] Selby MJ, Engelhardt JJ, Johnston RJ, Lu LS, Han M, Thudium K (2016). Correction: preclinical development of Ipilimumab and Nivolumab combination immunotherapy: mouse tumor models, in vitro functional studies, and Cynomolgus macaque toxicology. PLoS One..

[324] Krishnamurthy A, Jimeno A (2017). Atezolizumab: A novel PD-L1 inhibitor in cancer therapy with a focus in bladder and non-small cell lung cancers. Drugs Today..

[325] Rosenberg JE, Hoffman-Censits J, Powles T, van der Heijden MS, Balar AV, Necchi A (2016). Atezolizumab in patients with locally advanced and metastatic urothelial carcinoma who have progressed following treatment with platinum-based chemotherapy: a single-arm, multicentre, phase 2 trial. Lancet..

[326] Ryu R, Ward KE (2018). Atezolizumab for the First-Line Treatment of Non-small Cell Lung Cancer (NSCLC): Current Status and Future Prospects. Front Oncol..

[327] Rosenberg JE, Hoffman-Censits J, Powles T, van der Heijden MS, Balar AV, Necchi A (2016). Atezolizumab in patients with locally advanced and metastatic urothelial carcinoma who have progressed following treatment with platinum-based chemotherapy: a single arm, phase 2 trial. Lancet..

[328] Ning Y-M, Suzman D, Maher VE, Zhang L, Tang S, Ricks T (2017). FDA approval summary: Atezolizumab for the treatment of patients with progressive advanced urothelial carcinoma after platinum-containing chemotherapy. Oncologist..

[329] Syed YY (2017). Durvalumab: first global approval. Drugs..

[330] Powles T, Galsky MD, Castellano D, Van Der Heijden MS, Petrylak DP, Armstrong J (2016). A phase 3 study of first-line durvalumab (MEDI4736) ± tremelimumab versus standard of care (SoC) chemotherapy (CT) in patients (pts) with unresectable Stage IV urothelial bladder cancer (UBC): DANUBE. J Clin Oncol..

[331] Antonia SJ, Villegas A, Daniel D, Vicente D, Murakami S, Hui R (2017). Durvalumab after Chemoradiotherapy in stage III non-small-cell lung Cancer. N Engl J Med..

[332] Juliá EP, Amante A, Pampena MB, Mordoh J, Levy EM (2018). Avelumab, an IgG1 anti-PD-L1 immune checkpoint inhibitor, Triggers NK Cell-Mediated Cytotoxicity and Cytokine Production Against Triple Negative Breast Cancer Cells. Front Immunol..

[333] Kaufman HL, Russell J, Hamid O, Bhatia S, Terheyden P, D'Angelo SP (2016). Avelumab in patients with chemotherapy-refractory metastatic Merkel cell carcinoma: a multicentre, single-group, open-label, phase 2 trial. Lancet Oncol..

[334] Apolo AB, Infante JR, Balmanoukian A, Patel MR, Wang D, Kelly K (2017). Avelumab, an anti–programmed death-ligand 1 antibody, in patients with refractory metastatic urothelial carcinoma: results from a multicenter, Phase Ib study. J Clin Oncol..

[335] Patel MR, Ellerton J, Infante JR, Agrawal M, Gordon M, Aljumaily R (2018). Avelumab in metastatic urothelial carcinoma after platinum failure (JAVELIN solid tumor): pooled results from two expansion cohorts of an open-label, phase 1 trial. Lancet Oncol..

[336] Bertrand A, Kostine M, Barnetche T, Truchetet M-E, Schaeverbeke T (2015). Immune related adverse events associated with anti-CTLA-4 antibodies: systematic review and meta-analysis. BMC Med..

[337] Giles AJ, Hutchinson MND, Sonnemann HM, Jung J, Fecci PE, Ratnam NM (2018). Dexamethasone-induced immunosuppression: mechanisms and implications for immunotherapy. J Immunother Cancer..

[338] Medina PJ, Adams VR (2016). PD-1 pathway inhibitors: Immuno-oncology agents for restoring antitumor immune responses. Pharmacotherapy..

[339] Leonardi GC, Gainor JF, Altan M, Kravets S, Dahlberg SE, Gedmintas L (2018). Safety of programmed Death-1 pathway inhibitors among patients with non-small-cell lung Cancer and preexisting autoimmune disorders. J Clin Oncol..

[340] Chae YK, Arya A, Iams W, Cruz MR, Chandra S, Choi J (2018). Current landscape and future of dual anti-CTLA4 and PD-1/PD-L1 blockade immunotherapy in cancer; lessons learned from clinical trials with melanoma and non-small cell lung cancer (NSCLC). J Immunother Cancer..

[341] Jhawar SR, Thandoni A, Bommareddy PK, Hassan S, Kohlhapp FJ, Goyal S (2017). Oncolytic viruses—natural and genetically engineered Cancer immunotherapies. Front Oncol..

[342] McCormick F (2015). KRAS as a therapeutic target. Clin Cancer Res..

